# Comparison of quantitative trait loci methods: Total expression and allelic imbalance method in brain RNA-seq

**DOI:** 10.1371/journal.pone.0217765

**Published:** 2019-06-17

**Authors:** Jesper R. Gådin, Alfonso Buil, Carlo Colantuoni, Andrew E. Jaffe, Jacob Nielsen, Joo-Heon Shin, Thomas M. Hyde, Joel E. Kleinman, The BrainSeq Consortium, Niels Plath, Per Eriksson, Søren Brunak, Michael Didriksen, Daniel R. Weinberger, Lasse Folkersen

**Affiliations:** 1 Cardiovascular Medicine Unit, Center for Molecular Medicine, Department of Medicine, Karolinska Institutet, Stockholm, Karolinska University Hospital, Solna, Sweden; 2 Roskilde Hospital, Roskilde, Denmark; 3 Lieber Institute for Brain Development, Baltimore, United States of America; 4 Lundbeck A/S, Valby, Denmark; 5 Center for Protein Research, University of Copenhagen, Copenhagen, Denmark; Harvard Medical School, UNITED STATES

## Abstract

**Background:**

Of the 108 Schizophrenia (SZ) risk-loci discovered through genome-wide association studies (GWAS), 96 are not altering the sequence of any protein. Evidence linking non-coding risk-SNPs and genes may be established using expression quantitative trait loci (eQTL). However, other approaches such allelic expression quantitative trait loci (aeQTL) also may be of use.

**Methods:**

We applied both the eQTL and aeQTL analysis to a biobank of deeply sequenced RNA from 680 dorso-lateral pre-frontal cortex (DLPFC) samples. For each of 340 genes proximal to the SZ risk-SNPs, we asked how much SNP-genotype affected total expression (eQTL), as well as how much the expression ratio between the two alleles differed from 1:1 as a consequence of the risk-SNP genotype (aeQTL).

**Results:**

We analyzed overlap with comparable eQTL-findings: 16 of the 30 risk-SNPs known to have gene-level eQTL also had gene-level aeQTL effects. 6 of 21 risk-SNPs with known splice-eQTL had exon-aeQTL effects. 12 novel potential risk genes were identified with the aeQTL approach, while 55 tested SNP-pairs were found as eQTL but not aeQTL. Of the tested 108 loci we could find at least one gene to be associated with 21 of the risk-SNPs using gene-level aeQTL, and with an additional 18 risk-SNPs using exon-level aeQTL.

**Conclusion:**

Our results suggest that the aeQTL strategy complements the eQTL approach to susceptibility gene identification.

## Background

The large-scale efforts of genome-wide association studies (GWASs) have in recent years discovered hundreds of single-nucleotide polymorphisms (SNPs) associated with common complex disease. The high reproducibility and well-supported causal link to disease means that these risk SNPs represent precious knowledge towards advances in drug development. However, so far, only few examples of actual implementation as drug candidates exist. We believe that the main reason for this is the lack of a precise understanding of how risk SNPs affect genes and, ultimately, druggable targets. Linking risk SNPs with risk genes is of major importance, and currently, this has been of only limited success.

**Fig 1:**
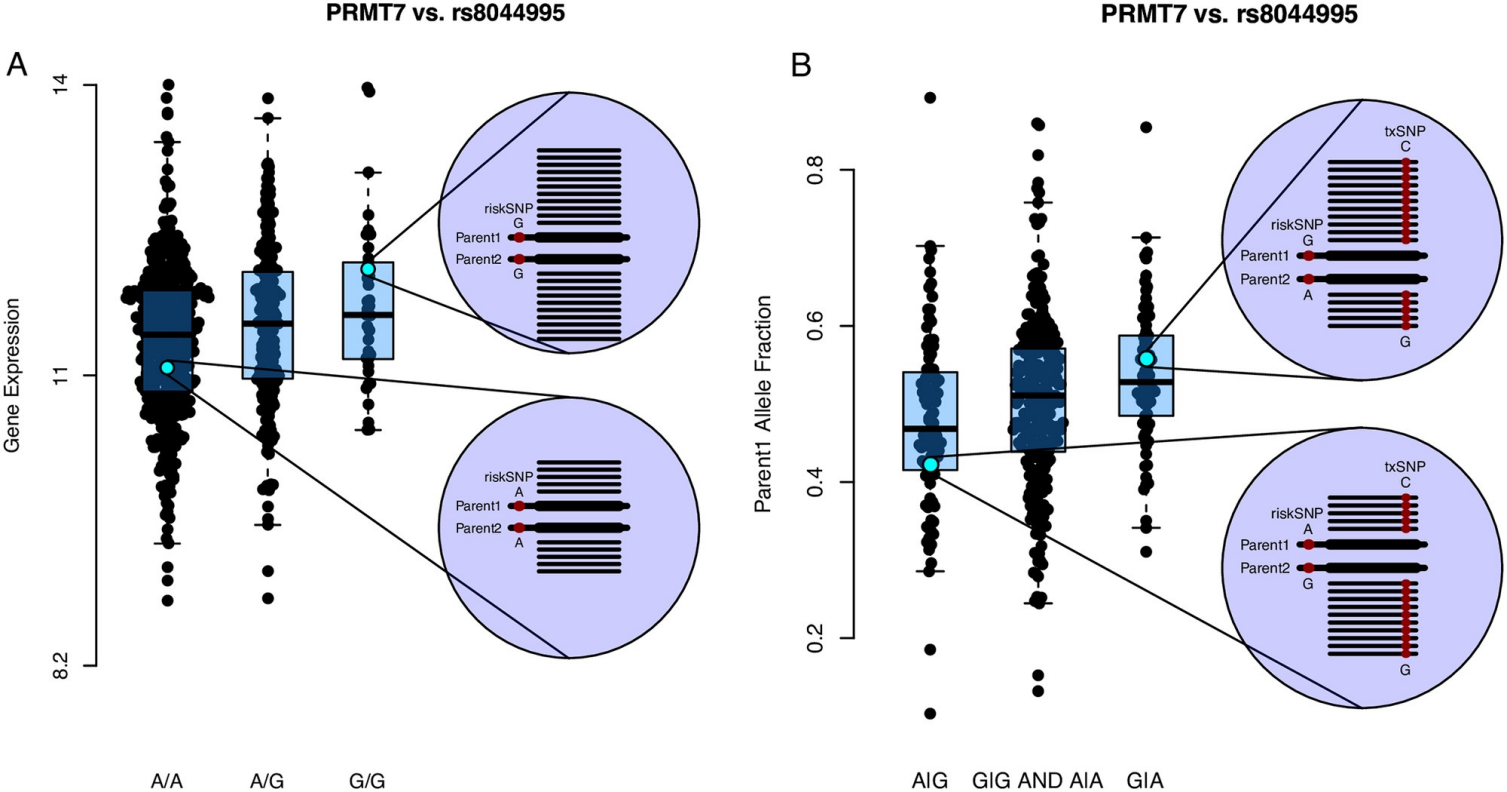
Comparison of eQTL and aeQTL for *PRMT7* and the *rs8044995* risk-SNP. **(A)** In a standard eQTL analysis the X-axis (input variable) is the genotype of the risk-SNP and the Y-axis (response variable) is the expression of the gene. **(B)** aeQTL analysis of the same risk-snp/gene pair. The Y-axis shows the fraction of reads coming from the Parent 1 chromosome in the PRMT7 gene. Only samples with a heterozygous txSNP are included, and in samples with more than one heterozygous txSNP the average fraction is used. The X-axis shows the risk-SNP group: samples that are heterozygote with an A-allele on the Parent 1 chromosome are in group 1, samples that are homozygote (A/A or G/G) are in group 2, and samples with a G-allele on the Parent 1 chromosome are in group 3. The linear regression of these values constitutes the basis of the aeQTL model. The supplementary S2 File provides plots for all gene-SNP pairs that are listed in Table 1.

One reason for this is that the vast majority of risk SNPs are non-coding and thus without an immediately obvious culprit gene. Typically, only 10% of GWAS-identified risk SNPs affect the protein sequence directly or through high-linkage disequilibrium (LD) proxies [1]. In the search for gene identification, most risk SNPs have been analyzed using eQTL methods, which often cases can link a non-transcribed SNP to the expression of a proximal gene [2] [3]. The eQTL method has so far identified a functional association for 30–50% of risk SNPs in brain [2, 4, 5]. In the case of the Schizophrenia (SZ) GWAS original report, 16% of the findings were shown to have brain eQTL effects at the gene level [1], but further studies are increasing this number [6, 7]. The current state of the art analysis of GWAS SNPs and all expression features in the transcriptome suggests that over 50% of the GWAS SNPs are associated with transcription [7]. Understanding the mechanisms and mediator-genes of these risk-SNPs are of primary importance for translation of the genetic findings. For this reason, we set out to compare allelic expression quantitative trait loci (aeQTL) that serves the same purpose as an eQTL but is based on the allele fraction within each individual, instead of the total expression from both alleles. We use the term aeQTL to designate imbalances in expression due to stoichiometric variation of a specific genomic variation. We combined a computational phasing of genotypes with deep RNA-seq analysis to identify genes with strong imbalance as correlating with proximal heterozygous GWAS-identified risk-SNPs. For investigation of neighboring genes, the use of haplotypes can give us reproducible phase estimates even when LD is low (Figure 1 in S1 File). The concept is illustrated in Fig 1: In aeQTL, only samples where the gene has heterozygous transcribed SNPs (txSNPs) can have an allele fraction calculated. When heterozygous, the txSNPs function as reporters of allelic transcription from the risk chromosome copy. In this analysis we have used txSNPs within the exons of each gene investigated, and the allele fraction was set relative to the parent 1 phase, based on phasing results. Therefore, if there is a link with a risk-allele that increases gene expression, the allele fraction should attain one of three levels: i) >0.5 if the txSNP and the risk SNP are in one phase; ii) Close to 0.5 if the risk-SNP is homozygote, iii) <0.5 if the txSNP and the risk-SNP are in the opposite phase [8]. Thus, the allelic imbalance is a more subtle autosomal version of the well-known X-chromosome inactivation process [9].

We here present the results of comparing eQTLs and aeQTLs in a deeply sequenced biobank of brain samples, with the goal of illustrating how allelic imbalance genetics may serve as a possible complement.

## Methods

### Subjects and biopsies

A total of 782 individuals were included in the study. The Post-mortem human brain tissue was obtained by autopsy with informed consent from the legal next of kin (protocol 90-M-0142 approved by the NIMH/NIH Institutional Review Board), in accordance with the declaration of Helsinki. Brain samples were obtained from the dorsolateral pre-frontal cortex (DLPFC) (n = 680). Additionally, from the same individuals, DNA was isolated from cerebellar samples for the purpose of genome-wide genotyping and a set of hippocampal formation tissue (n = 421) was used for replication. The age of the individuals ranged from birth to 97 years of age, although a complementary analysis including pre-natal samples are found in Tables S5 and S6 in S1 File. The ethnicity was primarily Afro-American and Caucasian. The mean post-mortem interval was 33.4±20.1 minutes and the mean RIN-value was 8.1±0.9. Further details are described elsewhere [7].

### Genotyping and RNA sequencing

For genotyping, the Illumina Human1M-Duo v3.0, illumina 650K and omniX+ microarrays were used. Data was merged using Plink (1.90beta3). The Shapeit2 software (v2.r790) [10] was used for pre-phasing, and impute2 (version 2.3.2) for imputation and phasing, using the 1000genomes phase III reference panel.

**Fig 2:**
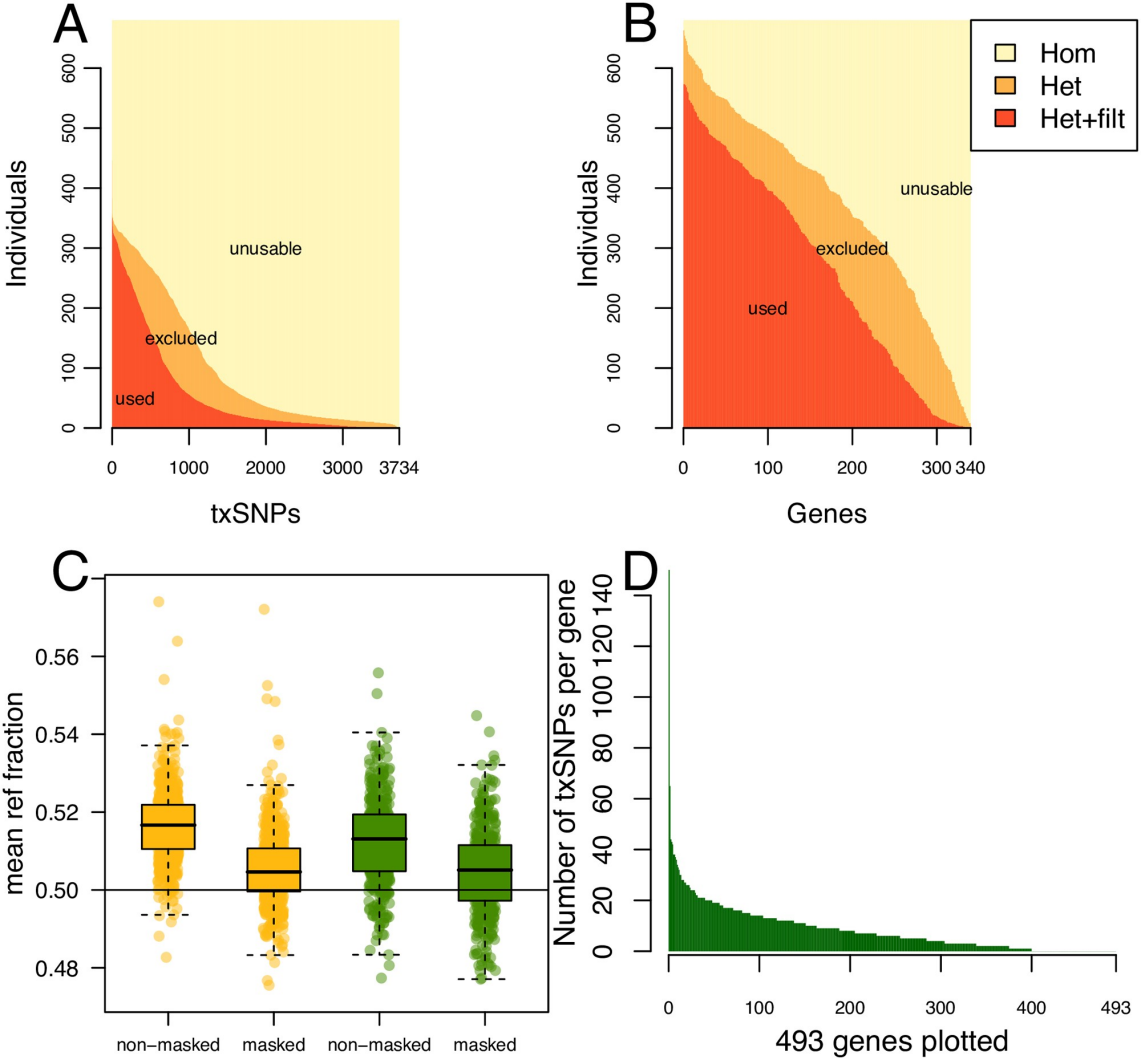
Filtration and mapping bias considerations in analysis pipeline. **(A)** Shows the least amount of individuals (Y-axis) for a given amount of txSNPs (X-axis) at different condition levels, namely *Het*, i.e. count of heterozygous SNPs, *Het+filt*, i.e. the count of heterozygous SNPs that pass QC filtration, and *Hom*, i.e. the remaining SNPs, which are homozygous. **(B)** Similar, but instead with an X-axis showing gene-count for which at least one txSNP fulfills the condition level. In other words, the number analyzable individuals for a given amount of the genes of interest. **(C)** The fraction of reference-allele containing reads measured over all txSNPs, dependent on using a masked or a non-masked genome. The horizontal line at 0.5 indicates the expected fraction under the assumption of no mapping bias. Yellow indicates DLPFC tissue, green indicates Hippocampus tissue. **(D)** Number of txSNPs in the genes of interest with at least one heterozygote sample among the 782 analyzed individuals.

For the RNA-sequencing of DLPFC and Hippocampus, the library was prepared either by depletion of rRNAs by RiboZero or by a polyA+ extraction of all fragments containing a poly-A tail. Two samples per lane were used producing an average of 129.8 million paired-end reads per sample (Figure 2 and Table S1 in S1 File. Further data access is available at the Lieber Institute’s online data browser (http://eqtl.brainseq.org).

### Data pre-processing

STAR software (2.4.2a) was used to align the RNA-sequencing data to the human genome (assembly GRCh37). This was done both in a non-masked and masked version, where the masked reference genome additionally used the ambiguity nucleotide character N for all SNPs in the UCSC track snps142Common, (assembly GRCh37/hg19). Additional re-alignments with masking of RNA-seq-called SNPs were performed. Samtools mpileup (version 0.1.19) was used to discover the variant-positions in the RNA-seq data (*samtools mpileup—BCF—skip-indels—positions*), and bcftools was used to loosely pre-filter these (*bcftools filter—exclude ‘MIN(QUAL)<10 | MIN(DP)<10 | AC<10’*). To reduce multiple testing burden the analysis was restricted to include genes only within 200kb from the 108 SZ risk loci, this was motivated by the complex phasing reproducibility pattern shown in Figure 1 in S1 File. The AllelicImbalance package was then used to import relevant read counts at each of the discovered txSNPs [11]. The mapped reads were summarized as read counts for each allele type at each txSNP, and their phase information inferred from shapeit2 was added, which made it possible to calculate the phase specific fraction for parent 1. These *AllelicImbalance* data-objects were considered as the pre-processed basis of all further aeQTL analysis.

### Filtering, defining of regions and pairing of risk-SNPs

On the pre-processed AllelicImbalance objects we further set a threshold of at least 10 reads from each allele for a txSNP and restricted the analysis to exons including the 5’ and 3’ UTR. We also required that at least 10% of the total expression came from the less expressed allele, to hinder sequencing errors creating false allelic imbalance. All genes or txSNPs having at least 5 usable individuals in at least two genotype groups were used in the regression model. The amount of remaining usable individuals for the analysis can be seen in Fig 2A and 2B.

Further, txSNP-regions were defined according to the UCSC RefSeq gene annotation database (version 12-11-2015) in a way that each txSNP-region corresponded to one gene. The only exception was overlapping genes, which were included in the same txSNP-region. Using the phase information, we then summarized all txSNPs in the region to get a single value for each sample.

### Analyzing aeQTL through the use of phasing information

Our method for assessing aeQTLs was inspired by previous work by Almlöf et al, in which expression microarrays were used to obtain allele-specific expression information, and then combined with computationally phased genotypes to link the allele-specific expression with non-transcribed SNPs [8]. LD information is correlated, but not outperforming phasing using the 1000Genomes reference panel as illustrated in Figure 1 in S1 File. Two main parts were of importance: Firstly, as with all allelic imbalance based methods, this required the presence of a heterozygous SNP to discern between alleles. To maximize the number of testable genes, summarization of data from *all* txSNPs in a gene (txSNP-region) was therefore used. Secondly, since all genotypes were computationally phased, the relevant allelic imbalance fractions were always calculated in the parent-1-allele direction. Taken together this prompts the *fraction*_*P1*_ variable, which is defined as the number of parent-1 reads divided by reads from both chromosome copies, over all txSNPs in a gene (txSNP-region). Since the non-transcribed risk-SNP is also phased, the association between risk-SNP and the allelic imbalance it creates can be investigated using the linear regression model:

$${\text{fraction}}_{\text{p1}}∼\text{risk}-\text{SNP}-\text{group}+\text{covariates}$$

The risk-SNP-group value is enumerated as 0, 1 or 2; being either heterozygote of one phase, e.g. A|G, homozygote, e.g. A|A and G|G, or heterozygote in opposing phase, e.g. G|A (as illustrated in Fig 1A, and further detailed in Figure 1 in S1 File). Except when explicitly noted otherwise, we always included the covariates age and sex as well as the first three principal components of the genotyping data and the first ten principal components of the RNA-seq data. To correct for multiple testing we used a FDR 5% threshold, calculated over each risk-SNPs—gene pair (n = 371). For the splicing analysis, we used a separate threshold calculated over each risk-SNPs–txSNPs pair (n = 4032).

### Comparable eQTL analysis

The eQTL P-values used for comparison are the Lieber- BrainSeq consortium P-values obtainable from this website: http://eqtl.brainseq.org/. They are calculated from the same set of human brains, using the same covariates as for the aeQTL analysis, i.e. age, sex, three first genotype principal components and first ten expression principal components. Additionally, we linked comparable measurements from the Lieber BrainSeq consortium’s significant eQTL splice evidence for any corresponding aeQTL gene-level evidence.

## Results

### Pre-processing motivates masked alignment in aeQTL analysis

RNA sequence data from brain tissue for all genes proximal to the known SZ risk-SNPs were scanned for presence of heterozygous transcribed SNPs (txSNPs). In the 493 proximal genes, defined as all genes within 200 kb of the 108 risk loci, a total of 3734 heterozygous txSNPs were found in at least one of the analyzed individuals, illustrated in Fig 2D. With phasing, these heterozygous txSNPs could function as reporters of transcription from the risk chromosome copy. One set of filters were applied across all txSNPs: this included minimum read-quality, and minimum read-count. Another was applied on basis of each sample: filtration on the minimum expression level and on minimum percent of fraction for each allele. Altogether this left 340 of the 493 proximal genes amendable to analysis using the aeQTL approach (Fig 2A and 2B). Because of proximity of some risk-SNPs, the 128 risk-SNPs and the 340 genes, created 371 testable pairs of a total of 576 possible SNP-gene pairs in the region.

**Fig 3:**
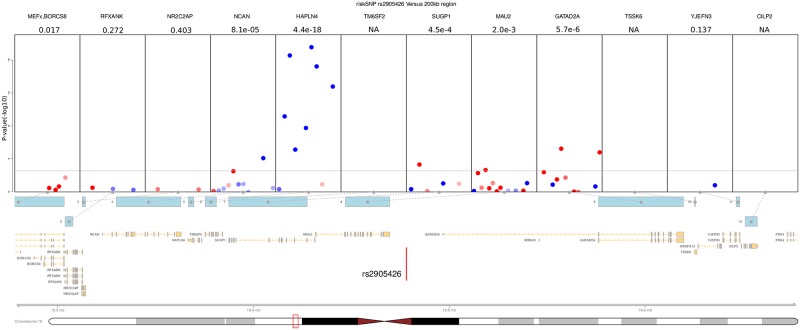
Plot of aeQTL effects in the genes surrounding the *rs2905426* risk-SNP. Each box in the annotationTrack shows one gene. Each dot shows one analyzable txSNP. The X-position of the dot indicates location in gene, and the Y-position indicates the aeQTL significance levels as–log10(P). The horizontal line indicates txSNP level significance at FDR5% (P = 0.0030). The color of each dot indicates effect-direction, red corresponds to increased fraction of ref-allele containing reads. The intensity of the color corresponds to the number of samples analyzable for the given txSNP, with 100 or more samples shown as fully saturated color. The GeneRegionTrack indicates the location of all the genes of interest surround this risk-SNP. The risk-SNP is indicated as a vertical red line. The top track contains the name of the gene as well as the P-value of the strongest associated txSNP.

**Fig 4:**
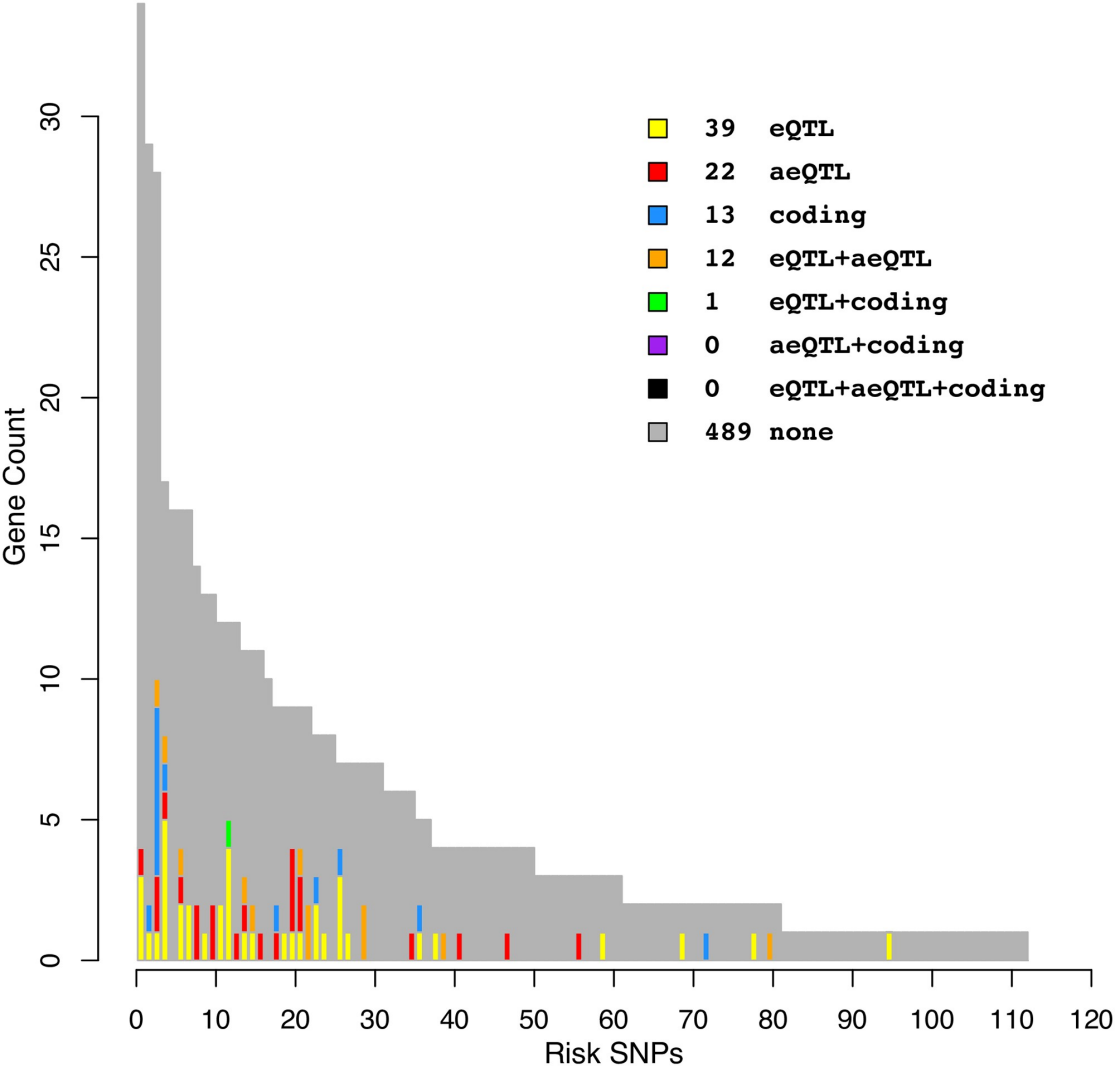
Overview comparing gene-level eQTL and aeQTL findings for the SZ risk-SNPs under analysis. Each bar corresponds to a single risk-SNP and the height of the bar shows the number of genes within 200kb. The color code indicates which of these genes have mechanistic evidence, either from previous or current work. Mechanistic evidence is here defined as either missense SNPs in proxy with the GWAS risk SNPs, gene-level eQTL effects, or aeQTL effects. Note that all 124 risk-SNPs in the 108 loci are shown, including 13 risk-SNPs with no genes within 200kb.

A potential problem in aeQTL analysis is the propensity for reference genome (ref-allele) allele containing reads to be favored over the alternative allele (alt-allele) during the alignment procedure, thereby introducing what is known as a mapping bias. Realignment of all raw reads was therefore performed using a reference genome that was masked at each of the 3734 txSNPs, referred to as the masked alignment. An aeQTL analysis was performed using both alignments, discovering 42 and 34 aeQTLs in the unmasked and masked alignments respectively (Figure 3 in S1 File). However, calculation of overall reference allele fraction in both the masked and unmasked data set showed that the reference allele was particularly favored when using an un-masked reference genome (Mean 0.515±0.14). Using a masked reference genome during the alignment shrinks the effect (Mean 0.505±0.14), Fig 2C, Table S2 in S1 File, which is consistent with results by others [12]. This finding justified the exclusive use of the masked alignment for all subsequent analyses. An additional investigation of mapping bias for the significant aeQTLs showed no significant effect on the result. Generated QQ-plots and plots for inspection of heteroscedasticity at the level of each significant aeQTL confirmed the usability of a linear regression model, Figs 1 to 52 in S2 File.

### Analysis of QTLs in brain tissue reveals novel causal risk genes for schizophrenia

Of the 340 proximal genes analyzed, we found evidence of risk-SNP mediated gene expression modulation for 30 genes, located proximal to 21 SZ risk-SNPs. This difference was a consequence of 9 risk-SNPs having aeQTL effects with more than one gene (see Fig 3), a concept which is in line with previous eQTL findings [13]. We found that 4 SNPs and 12 gene-SNP pairs did not previously have evidence of missense proxies or brain eQTL (bold text, Table 1). Fig 4 shows an overview of previously found gene-level brain eQTLs [1, 6, 7] and coding evidence [1] proximal to the 108 SZ risk loci.

**Table 1:** Overview of aeQTL findings in DLPFC tissue.

SNP	Pos	Gene	Dist (Kb)	Dist rank	Ref/Alt	Direction aeQTL/eQTL	aeQTL P	eQTL P (7)	Additional gene-SNP evidence	tx-SNPs	Reads	Individuals per aeQTL group	Protein	Tissue
**rs11210892**	chr1:44	**PTPRF**	12	1	**G**/A	down/-	1.40e-06	0.93	-	17	1500	129/287/121	Protein Tyrosine Phosphatase	DLPFC
rs140505938	chr1:150	VPS45	-8	1	**C**/T	up/down	0.0018	0.00249	Js (P = 1.9e-16)	5	87	19/74/21	Vacuolar Protein Sorting 45 Homolog	DLPFC
		**ANP32e**	-160	8	**C**/T	down/-	7.20e-10	0.337		8	670	49/311/54	LANP-L	DLPFC
rs6670165	chr1:177	BRINP2	29	1	C/**T**	down/-	7.00e-13	0.0989	Js (P = 2.2e-8)	4	1600	76/250/88	BRINP2	DLPFC
rs7523273	chr1:208	CD46	8	1	**A**/G	up/up	1.00e-15	1.1e-33	Js (P = 9.2e-38)	9	250	137/109/118	CD46	DLPFC
rs6434928	chr2:198	HSPD1	-47	3	**G**/A	down/-	0.00018	0.3	Js (P = 3.3-14)	6	260	136/186/119	Chaperonin 60	DLPFC
		HSPD1	-47	3	**G**/A	down/-	1.50e-05	0.76		6	370	70/147/68	-	HIPPO
		**HSPe1-MOB4**	-60	4	**G**/A	up/-	5.10e-06	0.358	-	9	51	121/128/113	Chap. 10, Mps One Binder Kinase Activator-Like 3	DLPFC
rs2535627	chr3:53	**SPCS1**	103	6	**T**/C	down/-	0.00000003	0.142	-	3	230	116/119/98	Signal Peptidase Complex Subunit 1	DLPFC
		NeK4	40	4	**T**/C	up/up	1.30e-05	0.0000015	Js (P = 1.3e-8)	9	44	134/153/128	NIMA Related Kinase 4	DLPFC
rs9841616 (chr3_180594593_I)	chr3:181	**DNAJC19**	-107	2	**D**/I	up/-	0.0014	0.926	-	2	54	113/17/124	Mito. Inner membr. translocase subunit TIM14	DLPFC
rs10043984	chr5:138	FAM53C	27	2	C/**T**	down/-	0.00046	0.432	Js (P = 5.6e-06)	9	99	94/239/82	FAM53C	DLPFC
rs111896713 (chr5_140143664_I)	chr5:140	ZMAT2	58	2	D/**I**	down/up	5.60e-20	0.000000197	Js (P = 2.2e-8)	2	250	155/32/129	Zinc finger matrin-type protein 2	DLPFC
		ZMAT2	58	2	D/**I**	down/down	2.20e-08	0.048		2	180	94/30/73	Zinc finger matrin-type protein 2	HIPPO
		IK	102	7	D/**I**	down/up	0.0005	0.012	-	5	150	91/118/70	Cytokine IK	DLPFC
		NDUFA2	116	8	D/**I**	down/down	4.20e-11	0.00596	Rg (P = 2.2e-5)	2	76	129/77/96	Ubiquinone Oxidoreductase Sub-u. A2	DLPFC
**rs1339227**	chr6:73	**RIMS1**	43	1	**C**/T	down/-	0.0013	0.128	-	21	440	120/338/103	Reg. Synaptic Membr. exocytosis 1	DLPFC
rs11191419	chr10:105	AS3MT-BORCS7	-2	1	**T**/A	up/up	6.40e-20	1.88e-25	Rg (P = 2.6e-5), Js (P = 2.0e-59)	20	130	140/132/143	Arsenite Methyltransferase	DLPFC
		AS3MT-BORCS7	-2	1	**T**/A	up/up	4.90e-08	5.10e-14		20	99	84/55/84	Arsenite Methyltransferase	HIPPO
rs55833108	chr10:105	AS3MT-BORCS7	80	2	G/**T**	down/-	0.0022	0.0684	-	20	130	38/336/41	Arsenite Methyltransferase	DLPFC
rs10791097	chr11:131	SNX19	-28	1	**T**/G	up/up	9.50e-08	2.3e-10	Js (P = 3.2e-29))	44	710	146/293/134	Sorting Nexin 19	DLPFC
		SNX19	-28	1	T/G	up/up	1.70e-06	0.024		44	1000	93/196/86	Sorting Nexin 19	HIPPO
**rs12826178**	chr12:58	**LRP1**	16	4	**G**/T	down/-	3.20e-05	0.383	-	32	130	24/413/23	LDL Receptor Related Protein 1	DLPFC
rs12887734	chr14:104	APOPT1	0	1	G/**T**	down/down	5.40e-44	1.12e-10	Js (P = 3.0e-22)	8	6300	119/57/109	Apoptogenic 1	DLPFC
		**CKB**	59	4	G/**T**	down/-	2.20e-07	0.617	-	3	9200	66/249/65	Creatine Kinase B	DLPFC
		**PPP1R13B**	-153	8	G/**T**	down/down	0.0006	0.0166	-	9	40	38/107/37	Phosphatase 1 Regulatory Sub-u. 13B	DLPFC
**rs56205728**	chr15:41	**BUB1B-PAK6**	0	1	G/**A**	down/down	0.0014	0.049	-	38	1200	112/329/98	Mitotic checkpoint ser/ Ser/thr-protein kinase	DLPFC
rs4702	chr15:91	FURIN	0	1	**G**/A	down/down	2.70e-11	0.00175	Fg (P = 1.0e-6)	10	64	133/207/135	Membrane Associated Receptor Prot.	DLPFC
rs12691307	chr16:30	FAM57B	-96	12	**A**/G	up/up	1.70e-07	0.00815	Js (P = 5.1e-6))	4	310	125/157/140	FP1188	DLPFC
rs8044995	chr16:68	PRMT7	-156	7	G/**A**	down/down	0.00081	1.96e-05	Rg (P = 2.2e-5), Js (P = 1.0e-10))	9	530	82/265/95	Protein Arginine Methyltransferase 7	DLPFC
		**LCAT-SLC12A4**	191	9	G/**A**	down/-	0.00039	0.358	-	11	18	53/98/66	Lecithin-Cholesterol Acyltransferase, Solute Carrier Family 12 Member 4	DLPFC
rs8082590	chr17:18	TOM1L2	186	8	**G**/A	down/down	6.70e-12	0.00161	Js (P = 3.8e-05))	24	390	133/139/146	Target Of Membrane Trafficking Prot.	DLPFC
rs2905426	chr19:19	GATAD2A	-98	4	**G**/T	down/down	9.70e-05	1.82e-10	Js (P = 1.1e-8)	15	82	127/147/133	GATA Zinc Finger Domain Contain. 2A	DLPFC
		**HAPLN4**	109	5	**G**/T	up/-	6.70e-19	0.979	-	12	240	100/153/102	Brain Link Protein 2	DLPFC

All pairs of risk-SNPs and genes at 200 kb range were investigated and are included in the table if they have a FDR5% significant aeQTL effect (P < 0.0025). The risk-SNPs are highlighted in bold when no other functional evidence exists for that loci, i.e. no coding proxy (1), no gene-level brain eQTL (1,6,7) and no splice-level brain eQTL (7). Similarly, the genesymbol is highlighted in bold when no other functional evidence exists for that gene-SNP pair (same criteria). Pos: position. Dist: distance in kb. Dist rank: rank of gene by proximity to risk-SNP. Ref/Alt: reference and alternative allele. Bold indicates the SZ-risk increasing allele. Dir: gene expression direction induced by risk allele, indicated both for eQTL and aeQTL, when significant. txSNP: count of transcribed SNPs in gene with at least one heterozygote sample. Reads: The mean read coverage of the individuals for the txSNPs with highest coverage. N group: count of samples with at least one heterozygous txSNP stratified by risk-SNP groups (as defined in Fig 1A). Further gene-SNP evidence: Js = Jaffe-splice-QTL, Fg = Fromer-gene-eQTL (CommondMind), Rc = Ripke-Coding, Rg = Ripke-gene-eQTL. Tissue: DLPFC, dorso-lateral pre-frontal cortex. HIPPO: comparative analysis in hippocampus data.

The main goal of this analysis was to introduce the novel concept of aeQTL risk-SNP analysis and show how it adds to the eQTL method by increasing new QTL discoveries. Of the 34 significant aeQTLs in this analysis, 30 unique SNP-gene pairs were discovered by aeQTL at FDR5%, 10 SNP-gene pairs had FDR5%-significant direction-consistent evidence from both eQTL and aeQTL, and 6 additional FDR5%-significant aeQTL associations had nominal eQTL significance (P<0.05) in the same direction. These counts are further detailed in Table 1 in the column “direction aeQTL/eQTL”, wherein pairwise significance and direction are indicated for every SNP-gene pair that had FDR5% significant aeQTL effects. Obversely, 55 tested SNP-gene pairs were found as eQTL but not aeQTL.

In the two investigated brain regions, we found that the majority of expression association evidence was found in DLPFC, and not confirmed in hippocampus. Of the 30 SNP-gene pairs observed by aeQTL, 4 were replicated in the smaller set of hippocampus data. Since the DLPFC data set was slightly larger than the hippocampus (n = 680 vs n = 421), we repeated this analysis with a downsampling of the DLPFC, still obtaining overrepresentation of DLPFC findings (Figure 4 in S1 File). There were not enough fetal samples to conduct a separate analysis to highlight developmental genes, but including the additional 58 fetal samples in the cohort to the main analysis can suggest genes more sensitive to developmental changes. We found 11 other associations, 8 in DLPFC and 3 in hippocampus, all borderline significant except *CENPM* (p = 2.4e-12) and *EFTUD1P1* (p = 2.8e-05) for eQTL, and the *“GNL3*, *SNORD19*, *SNORD69*, *SNORD19B”* region (7e-04) for aeQTL. The inclusion of the 58 fetal samples also resulted in a loss of 7 borderline significant associations, 5 in DLPFC and 2 in hippocampus (Tables S5 and S6 in S1 File). Overall, these findings indicate that novel gene associations can be established with aeQTL, that there is overlap between this method and the eQTL method, and that DLPFC tissue contains more transcriptomic associations of SZ risk-SNPs than hippocampal tissue.

### Detailed investigation of each txSNP reveals possible splice effects

Although each gene only has a limited amount of analyzable txSNPs, we explored if the aeQTL-differences in the txSNP of each gene could nonetheless be used to describe possible splice effects. We expect neighboring txSNPs within the same gene should show similar effect-size and direction, if they are not affected by splice effects. Conversely, splice-effects may be observable as differences in effect-size and direction. We therefore investigated each txSNP in the genes of interest, illustrated by Fig 3 that shows the example of HAPLN4 (all others are available as Figures 1 to 65 in S3 File). While the HAPLN4 region showed an example of consistent unidirectional aeQTL effects from all txSNP in a single gene, this was not always the case: the txSNP-level plotting revealed a number of cases with non-significant gene-level metrics, but strong independent aeQTL-effects from some txSNPs. Taking the increased multiple-testing burden into account we found 156 txSNPs with aeQTL effect significant at FDR5% level (Tables S3 and S4 in S1 File), associating 18 more risk-SNPs not found by aeQTL at the gene level. The txSNP aeQTL approach could conceivably be used for a similar purpose as splice eQTL investigations described elsewhere [7].

### Interaction analysis provides possible explanations of discrepancy between eQTL and aeQTL

To study the discrepancies between eQTL and aeQTL and attempt to discover their causes we re-calculated all QTLs using covariates likely to have influence. These included RNA-integrity number (RIN), age, sex, diagnosis and smoking status. Particularly for RIN-value we wished to test the hypothesis that aeQTL was less affected by changes in RNA quality. While attractive as an example case, the systematic analysis did not completely support that RIN value is a main covariate for all risk-SNP gene pairs; using a binary cutoff at RIN 8, eQTL analysis showed 5 pairs with interaction effects, in aeQTL analysis only 1 pair did (Figure 5 in S1 File). Some examples of this were found, including the gene *LRRN3* and risk-SNP *rs13240464* which showed significant interactions with RIN-values(P_interaction_ = 1.36e-04), raising an uncertainty regarding the main effect of the eQTL at lower RNA qualities, as the low RIN samples show a significant eQTL (P = 0.00049) (Figure 6A in S1 File): The aeQTL analysis for the same risk-SNP gene pair showed no significance interaction (P_interaction_ = 0.37), and showed no significant aeQTL for low RIN-value (P = 0.51) (Figure 6B in S1 File). In Figure 7 in S1 File, eQTL appears more sensitive to RIN covariates than aeQTL, and we can also see that addition of more covariates has a higher effect on eQTL. In particular, including genetic and expression PCs changes the p-value more than for RIN, which suggests that for brain tissue more covariates than the RIN value is likely to be important. The use of aeQTL can be used to partially circumvent these covariates, and be a good compliment to standard eQTL analysis. Likewise, the remaining covariate analyses gave some possible explanatory examples, but were not able discover a fundamental explanation for discrepancies.

## Discussion

The eQTL approach is an established tool to link non-transcribed GWAS risk-SNPs with transcribed proximal genes. In this paper, we explore and compare with the possibly complimentary aeQTL approach. This method is inspired by previous microarray-based studies, in which phasing of genotypes is used to link txSNPs with a non-transcribed (risk) SNP [8]. We here refer to this linking as aeQTL analysis, and describe a study were the method is adapted to RNA-sequencing data, enabling the systematic uncovering of previously concealed expression-modulating effects from all known SZ GWAS-risk SNPs. The overall goal is to compare established (eQTL) and alternative tools (aeQTL) for functional linking of GWAS risk-SNPs and genes.

### Findings

In the previous brain eQTL analysis it was shown that 30 loci have gene-level eQTL and an additional 21 loci had splice- or exon-eQTL [7]. In this study, 16 and 6 of those loci were also identified using the gene-level and exon-level aeQTL approach, respectively, but not necessarily pointing towards the same gene. We speculate that these discrepancies may arise from the complexity of transcriptional regulation, such as for example how feedback loops may work to mask one method but not the other. While the goal of both eQTL and aeQTL is the same–to functionally investigate links between SNPs and genes–the approach is different. Clearly, the eQTL approach has potentially greater resolution because it does not require an expressed heterozygous SNP, as the comparison data show, but the aeQTL strategy may uncover additional genetic associations missed in the eQTL analysis. Overall, we found that 30 genes proximal to 21 known SZ risk-SNPs showed evidence of risk-SNP mediated allelic expression modulation (aeQTL).

Several of the findings emphasize already known links to brain functioning and development. In addition to eQTL-discoverable genes such as *CHRNA5*, *MAPK3* and *AS3MT* [14–16], these include the *HAPLN4* (Brain Link Protein 2), and the *RIMS1* (Regulating Synaptic Membrane Exocytosis 1). Several additional findings in Table 1 are of interest to follow-up with further studies: one signaling receptor, peptidase, translocase, transferase and two chaperonins together with several kinases and phosphatases. Systematic gene set enrichment analysis has already been performed in the initial GWAS as well as follow-up studies [1]; however, the main point to underscore is that such analysis often make the mistaken assumption that the closest gene is the mechanistically relevant gene. As shown in the *dist-rank* column of Table 1, and also evidenced by similar findings elsewhere [17], the closest gene is often not the functionally relevant gene. This was the case for 39 of 58 reported SNP-gene pairs in this analysis, which illustrates the strength of strengthening the use of functional genetics methods, such as eQTL and aeQTL, in order to arrive at more solid knowledge of *risk-genes* rather than just risk-SNPs and the genes next to them. Future studies might be further improved by combining this approach with colocalization analysis.

### Challenges of using the aeQTL method

The first challenge is that at least some samples need to be heterozygous for one or more txSNPs to produce a reliable regression model. A second problem is that some deviation from 1:1 may be ascribed to a mapping bias. Thirdly, to link the risk SNP with an AI event in the most effective manner, it is necessary to know the phase of the alleles, a challenge for which several solutions exists. In this paper, we present and apply calculation pipelines that address these three issues, and discuss what limitations remain for each.

To measure allelic imbalance, it is a requirement that a gene have one or more txSNPs that are heterozygous. This problem diminishes with larger sample sizes, as well as longer and less conserved genes. This problem was overcome in part by the creation of a system that summarized consecutive txSNPs within a gene evaluating their joint AI. This enabled us to investigate a total of 340 genes, thereby largely addressing the problem of lack of heterozygous txSNPs (Fig 2D).

The problem of mapping bias appears when RNA-seq reads containing the reference allele have larger likelihood of successful mapping, than those containing the alternative allele. By looking at the overall mean of all SNPs we observed that the average ref-allele fraction of all SNPs was different from the expected value of 0.5, indicating a modest but non-ignorable bias. To address this, we re-aligned the RNA-sequencing data using a version of the reference genome that was N-masked at all common SNP locations; this altered the ref-allele frequency to mean 0.505±0.14, reinforcing the notation that mapping bias is a potential issue in AI-calculations (see Fig 2C). Even if mapping bias is detrimental when looking at the allele frequency for only one individual, the problem is reduced if the bias is spread equally over all genotype groups i.e., the ref-allele is equally present over all genotype groups. Even though the summarization step is advantageous to increase the pool of heterozygote samples, there is a risk when using fraction mean that some reads overlap multiple txSNPs. Other approaches to this have been developed, include handling of the potential for very proximal txSNPs [18].

This situation is nevertheless not unique to aeQTL, as there exist a similar concern in eQTL studies, where RNA sequencing mapping bias or microarray allele specific hybridization may have an impact on the result. To test for any impact by mapping bias on our aeQTL after masking we performed a linear regression using the ref-allele fraction for each genotype group. Even if we could see remains of mapping bias for certain genes, none showed any significant relationship to the genotype group, supporting that our aeQTLs found were real and not due to technical artifacts (Supplementary Figures 1 to 52 in S2 File).

Finally, to link allelic imbalance with a (non-transcribed) risk SNP, phase has to be known in order to match the alleles of the txSNPs with the alleles of the risk SNP. The allele fraction calculated could suffer from uncertainty in phase generation, but this will be randomly distributed, introducing noise but not biasing the results. Other methods have been explored for doing this, by assuming that the AI for the heterozygous risk-SNPs should have a higher variance than the homozygote risk-SNPs [19], but we found the statistical power of such analysis is drastically reduced, and we could not find any associations in our data (Figures 1 to 52 in S2 File). From this we conclude that phasing-based methods are the stronger for linking txSNPs and non-transcribed SNPs.

Functionality for using these approaches has been implemented into the *AllelicImbalance* package, which is available in the Bioconductor repository [11].

Contrary to our initial hypothesis regarding the quality of samples from brain tissue and its confounding effects on eQTL, our results indicate a relatively little impact of RIN. This is in the same line as previous research where genotype did not significantly associate with RIN [20]. Recent work has demonstrated that RIN is not an adequate approach to RNA quality correction and that other methods may be indicated to further resolve this issue [20]. The increase of QTLs by using allelic imbalance information is more likely coming from its ability to remove the variation caused by trans-regulation of transcription.

## Conclusion

We have compared eQTL and aeQTL strategies in the context of human brain and schizophrenia risk SNPs. The main conceptual points we pursued in this study are i) the description of methodology for linking allelic imbalance with non-transcribed risk-SNPs in RNA-sequencing analysis and the ii) application of this methodology to a brain-tissue biobank. Overall, we find that the two methodologies have an overlap of significant QTLs, but also some differences where each method can find additional QTLs. We have discussed possible explanations for the differences, such as heterozygozity, read-depth, phasing, sample size and trans-regulation. However, further research will be required for determining the exact mechanism behind the differences. Taken together we believe these findings will have implications for how we use functional genetics to link GWAS risk-SNPs with genes.

## Supporting information

S1 File Collection of supplemental figures and tables. The File contains the supplemental figures S1-1 to S1-7, and supplemental tables S1-1 to S1-6.
(PDF)

S2 File Collection of supplemental figures and tables. The File contains the supplemental figures S2-1 to S2-34.
(PDF)

S3 File Collection of supplemental figures and tables. The File contains the supplemental figures S3-1 to S3-64.
(PDF)

S4 File Workflow overview. A more detailed description of the pre-processing and analysis workflow.
(PDF)

## Abbreviations

aeQTL: Allele specific expression quantitative trait loci
DLPFC: Dorso lateral prefrontal cortex
eQTL: Expression quantitative trait loci
GWAS: Genome wide association studies
LD: Linkage disequilibrium
SNP: Single nucleotide polymoprhism
SZ: Schizophrenia
txSNP: transcribed SNP

## References

[1] Schizophrenia Working Group of the Psychiatric Genomics Consortium. Biological insights from 108 schizophrenia-associated genetic loci. Nature 2014 7 24;511(7510):421–7. 10.1038/nature13595 25056061 PMC4112379

[2] Folkersen L, van’t Hooft F, Chernogubova E, Agardh HE, Hansson GK, Hedin U, et al Association of Genetic Risk Variants With Expression of Proximal Genes Identifies Novel Susceptibility Genes for Cardiovascular Disease. Circulation-Cardiovascular Genetics 2010 8;3(4):365–U306. 10.1161/CIRCGENETICS.110.948935 20562444

[3] Folkersen L, Biswas S, Frederiksen KS, Keller P, Fox B, Fleckner J. Applying genetics in inflammatory disease drug discovery. Drug Discov Today 2015 6 4. 10.1016/j.drudis.2015.05.012 26050580

[4] Okada Y, Wu D, Trynka G, Raj T, Terao C, Ikari K, et al Genetics of rheumatoid arthritis contributes to biology and drug discovery. Nature 2014 2 20;506(7488):376–81. 10.1038/nature12873 24390342 PMC3944098

[5] Wood AR, Esko T, Yang J, Vedantam S, Pers TH, Gustafsson S, et al Defining the role of common variation in the genomic and biological architecture of adult human height. Nat Genet 2014 11;46(11):1173–86. 10.1038/ng.3097 25282103 PMC4250049

[6] Fromer M, Roussos P, Sieberts SK, Johnson JS, Kavanagh DH, Perumal TM, et al Gene expression elucidates functional impact of polygenic risk for schizophrenia. Nat Neurosci 2016 11;19(11):1442–53. 10.1038/nn.4399 27668389 PMC5083142

[7] Jaffe A, Straub R, Shin J, Tao R, Gao Y, Torres J, et al. Developmental and genetic regulation of the human frontal cortex transcriptome in schizophrenia. Nat Neurosci 2017 B.C. Nov 11;In review. 10.1038/s41593-018-0197-y PMC6438700 30050107

[8] Almlof JC, Lundmark P, Lundmark A, Ge B, Maouche S, Goring HH, et al Powerful identification of cis-regulatory SNPs in human primary monocytes using allele-specific gene expression. PLoS One 2012;7(12):e52260 10.1371/journal.pone.0052260 23300628 PMC3530574

[9] Tukiainen T, Villani AC, Yen A, Rivas MA, Marshall JL, Satija R, et al Landscape of X chromosome inactivation across human tissues. Nature 2017 10 11;550(7675):244–8. 10.1038/nature24265 29022598 PMC5685192

[10] Delaneau O, Marchini J, Zagury JF. A linear complexity phasing method for thousands of genomes. Nat Methods 2011 12 4;9(2):179–81. 10.1038/nmeth.1785 22138821

[11] Gadin JR, van’t Hooft FM, Eriksson P, Folkersen L. AllelicImbalance: an R/bioconductor package for detecting, managing, and visualizing allele expression imbalance data from RNA sequencing. BMC Bioinformatics 2015;16:194 10.1186/s12859-015-0620-2 26066318 PMC4465016

[12] Degner JF, Marioni JC, Pai AA, Pickrell JK, Nkadori E, Gilad Y, et al Effect of read-mapping biases on detecting allele-specific expression from RNA-sequencing data. Bioinformatics 2009 12 15;25(24):3207–12. 10.1093/bioinformatics/btp579 19808877 PMC2788925

[13] Zhu Z, Zhang F, Hu H, Bakshi A, Robinson MR, Powell JE, et al Integration of summary data from GWAS and eQTL studies predicts complex trait gene targets. Nat Genet 2016 5;48(5):481–7. 10.1038/ng.3538 27019110

[14] Li M, Jaffe AE, Straub RE, Tao R, Shin JH, Wang Y, et al A human-specific AS3MT isoform and BORCS7 are molecular risk factors in the 10q24.32 schizophrenia-associated locus. Nat Med 2016 6;22(6):649–56. 10.1038/nm.4096 27158905

[15] Blizinsky KD, Diaz-Castro B, Forrest MP, Schurmann B, Bach AP, Martin-de-Saavedra MD, et al Reversal of dendritic phenotypes in 16p11.2 microduplication mouse model neurons by pharmacological targeting of a network hub. Proc Natl Acad Sci U S A 2016 7 26;113(30):8520–5. 10.1073/pnas.1607014113 27402753 PMC4968749

[16] Bhalala OG, Nath AP, Inouye M, Sibley CR. Identification of Brain Expression Quantitative Trait Loci Associated with Schizophrenia and Affective Disorders in Normal Brain Tissue. bioRxiv 2016. 10.1371/journal.pgen.1007607 PMC6126875 30142156

[17] Smemo S, Tena JJ, Kim KH, Gamazon ER, Sakabe NJ, Gomez-Marin C, et al Obesity-associated variants within FTO form long-range functional connections with IRX3. Nature 2014 3 20;507(7492):371–5. 10.1038/nature13138 24646999 PMC4113484

[18] Castel SE, Mohammadi P, Chung WK, Shen Y, Lappalainen T. Rare variant phasing and haplotypic expression from RNA sequencing with phASER. Nat Commun 2016 9 8;7:12817 10.1038/ncomms12817 27605262 PMC5025529

[19] Fogarty MP, Xiao R, Prokunina-Olsson L, Scott LJ, Mohlke KL. Allelic expression imbalance at high-density lipoprotein cholesterol locus MMAB-MVK. Human Molecular Genetics 2010 5 15;19(10):1921–9. 10.1093/hmg/ddq067 20159775 PMC2860891

[20] Jaffe AE, Tao R, Norris AL, Kealhofer M, Nellore A, Shin JH, et al qSVA framework for RNA quality correction in differential expression analysis. Proc Natl Acad Sci U S A 2017 7 3;114(27):7130–5. 10.1073/pnas.1617384114 28634288 PMC5502589

