# Supplementary material for: Comparison of quantitative trait loci methods: Total expression and allelic imbalance method in brain RNA-seq
Source: PLoS One. 2019 Jun 17;14(6):e0217765. doi: 10.1371/journal.pone.0217765 (PMC6576752; doi:10.1371/journal.pone.0217765)
Supplement: S1 File — The File contains the supplemental figures S1-1 to S1-7, and supplemental tables S1-1 to S1-6. (PDF) [file pone.0217765.s001.pdf]

Allelic Imbalance in SZ (Supplemental Material 1)

This is a document containing supplemental figures and tables for the “Comparison of Quantitative Trait Loci Methods: Total Expression and Allelic Imbalance Method in Brain RNA-seq” manuscript.

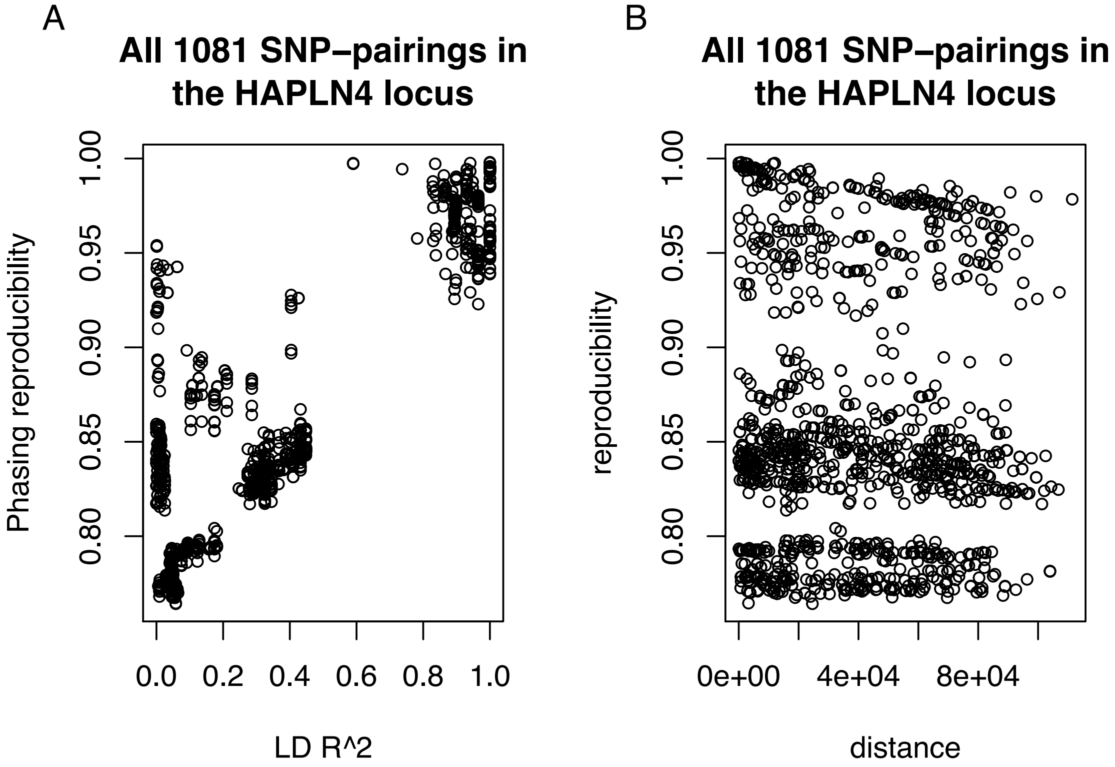

Fig S1-1: Illustration of the phasing quality of SNPs in the HAPLN4 region. All risk-SNP regions were phased using the Shapeit2 tool in order to link risk-SNPs with txSNPs. The fundamental mechanism of phasing is to determine likely parent of origin for all txSNPs that are heterozygous, by assigning alleles as being either of parent 1 (P1) origin or parent 2 (P2) origin. According to Shapeit2 notation, this is denoted with a vertical bar |, such that for example an A/G heterozygous SNP can be either A|G or G|A, meaning that the A-allele was inherited from the parent 1 or the parent 2, respectively.

A default execution of Shapeit2 provides one guess of likely haplotype, so the degree of probability is not directly available. However, as suggested by Shapeit2 authors a probability can be calculated by iterating through the same phasing using different random seeds. The phasing reproducibility between any two SNP is then defined as the fraction of iterations were the two SNPs have the same results with different random seeds; a value of 1 indicates perfect reproducibility and 0.5 indicates random choice.

We used this approach to investigate the relation beween reproducibility and linkage disequilibrium (LD) R2, as well as between reproducibility and distance. This is illustrated here for all pairwise combinations of SNPs in the HAPLN4 region. For A) reproducibility and LD, we un-surprisingly found that all SNP-pairs in perfect LD (R2=1) achieve almost completely perfect phasing reproducibility. However, importantly SNP-pairs that are not in LD (R2=0) also can achieve reproducibility-values above 0.9. Moreover, in this region no SNP-pair had worse reproducibility than 0.78. For B) reproducibility and distance, we found that at the investigated distance (400KB) there was only a weak tendency towards diminished phasing reproducibility with distance.

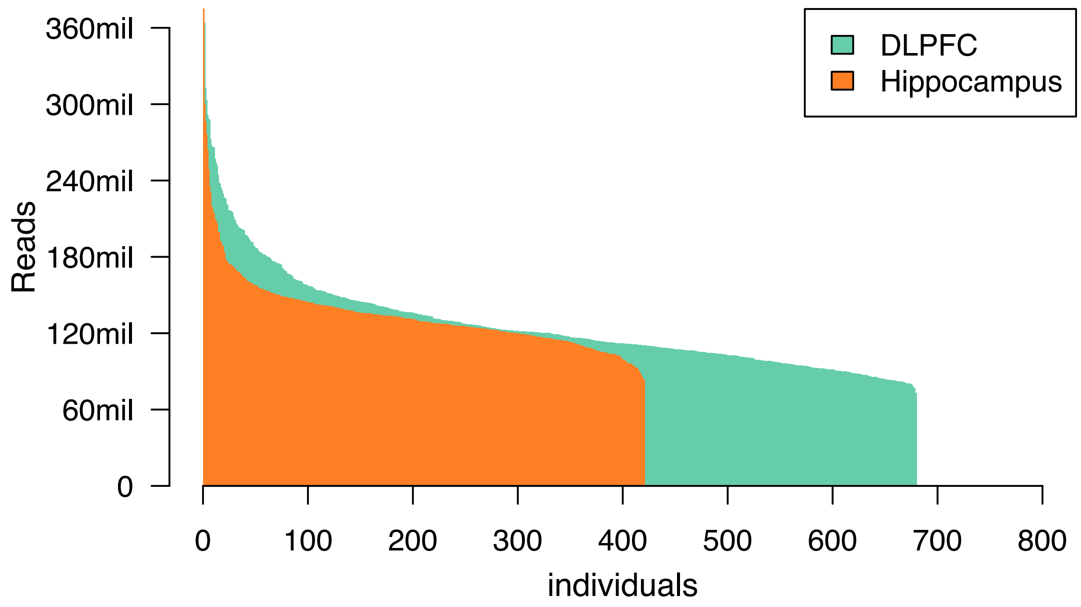

Fig S1-2: Pair-end reads in each fastq file for respective individual

	DLPFC	Hippocampus	Both
Mean	127133556	134174615	129825914
SD	39665006	30281344	36510189

Table S1-1: mean and standard deviation values for read counts in fastq files

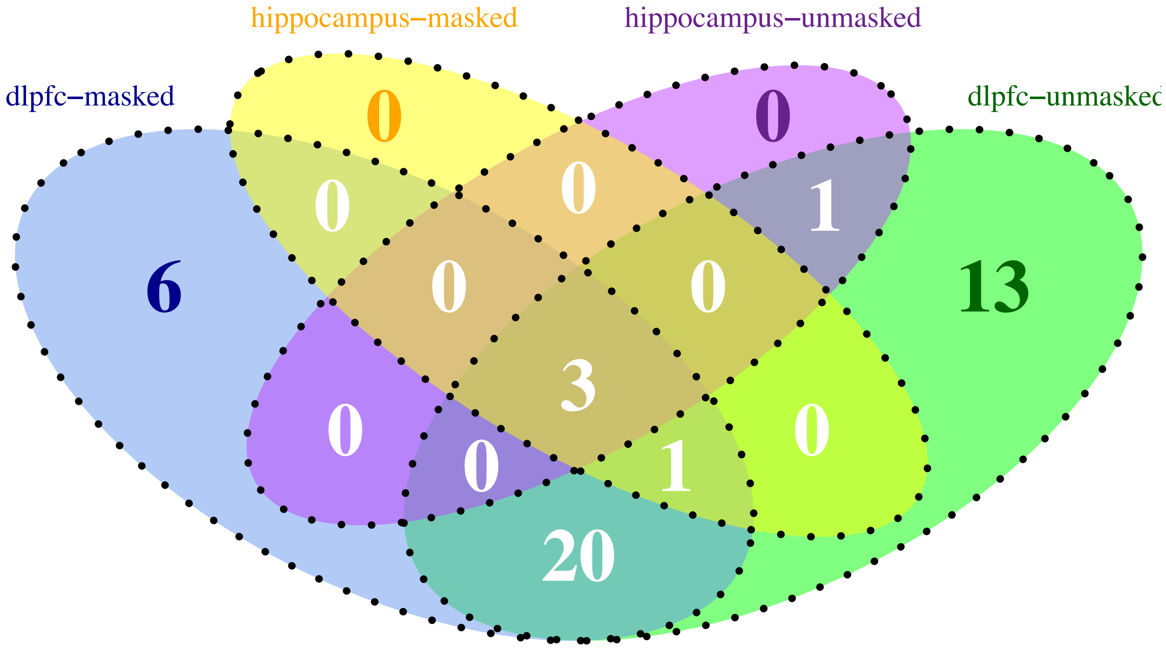

Fig S1-3: The number of aeQTLs found in respective tissue and alignment using significance thresholds set according to FDR5% as in main text table 1

	mean	sd	txSNPs
dlpfc.masked	0.506	0.127	247978
dlpfc.unmasked	0.517	0.125	239262
hippocampus.masked	0.505	0.161	137116
hippocampus.unmasked	0.513	0.160	135948

Table S1-2: A table of reference frequency and number of txSNPs in all genes and samples used for the calculation

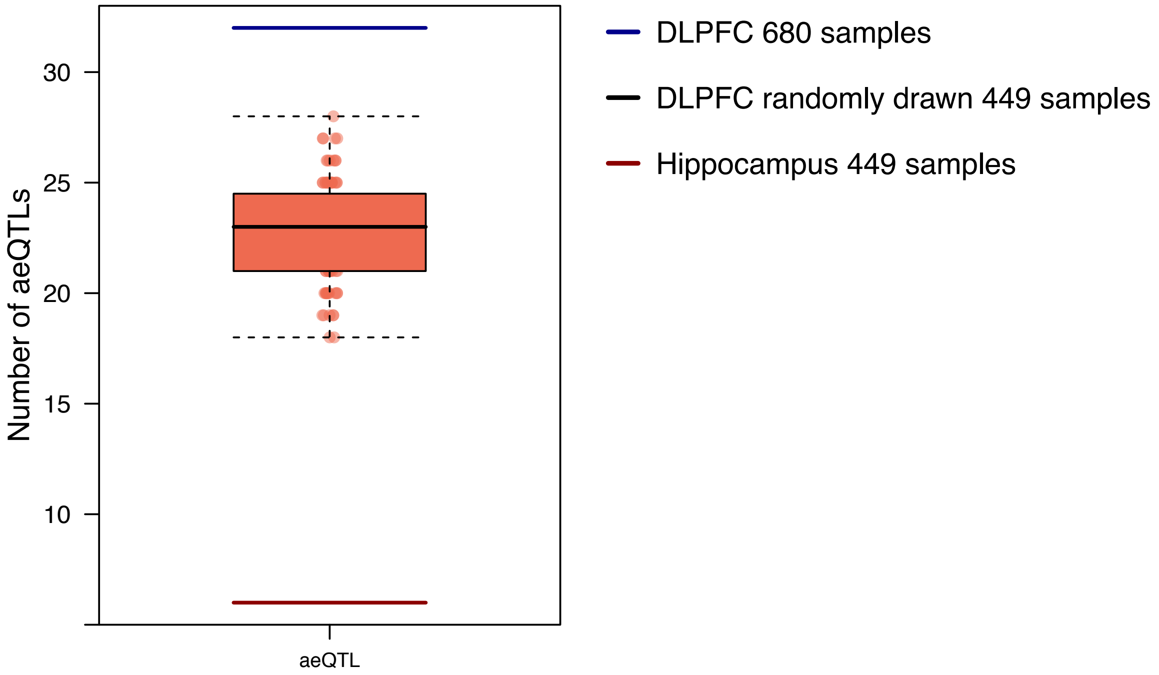

Fig S1-4: Figure demonstrating the amount of aeQTLs found for DLPFC(n=680) using the same sample size as Hippocampus(n=449). The DLPFC dataset was downsampled down to 449 and investigated for aeQTLs 100 times.

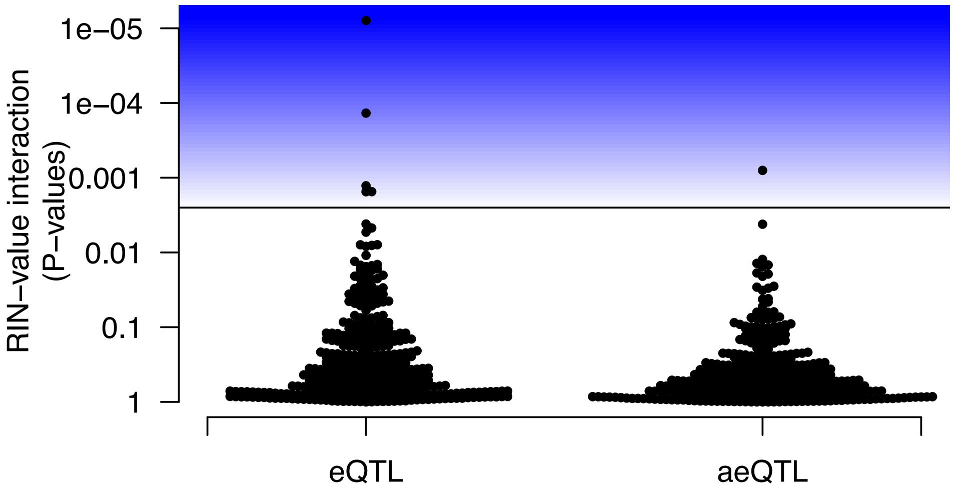

Fig S1-5: Calculation of RIN-interaction values for eQTL and aeQTL. Each dot shows the interaction P-value for all SNP-gene pairs. A binary RIN value cutoff was set at RIN 8. For the eQTL analysis 5 pairs were significant after multiple testing correction, in aeQTL analysis 1 pair was significant ($P<0.0025$).

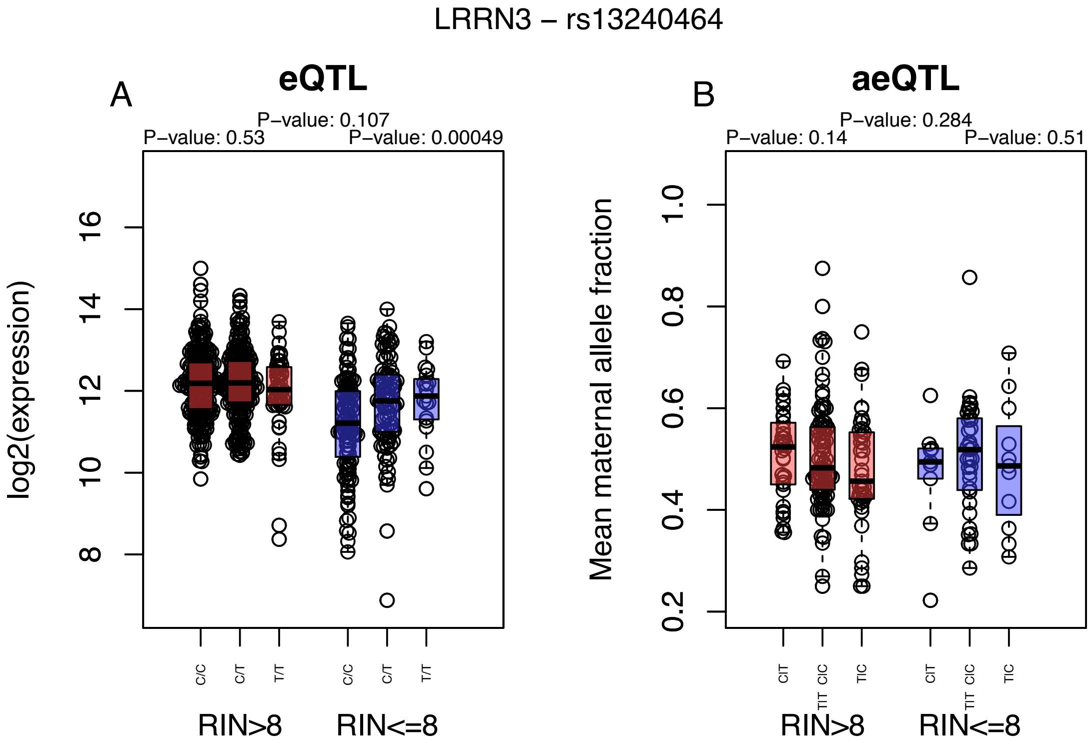

Fig S1-6: The eQTL for LRRN3 and risk-SNP rs13240464, stratified by RIN value. This plot illustrates an eQTL with potential interaction effects caused by RNA-degradation: when testing eQTL effect A), there was a significant interaction between RIN-value and risk-SNP genotype (Pinteraction=1.36e-04), with lower RIN values giving rise to a stronger eQTL effect. B) This was not the case for aeQTL (Pinteraction=0.37). Neither the non-stratified by RIN, but RIN-corrected eQTL (P= 0.107) nor the aeQTL (P= 0.284) for this gene-SNP pair were significant after multiple testing

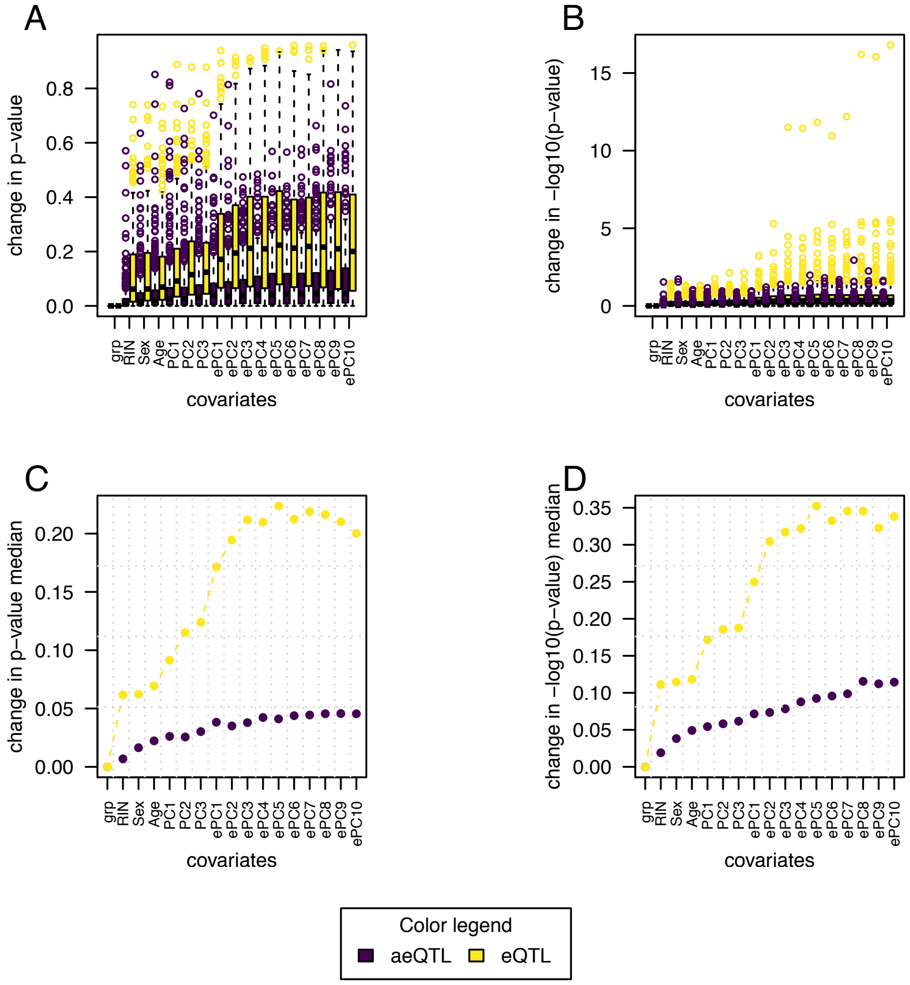

Fig S1-7: Covariates impact on p-values in aeQTL versus eQTL. All testable 371 gene-snp pairs in DLPFC have been included in this analysis, comparing the change of p-value distributions of aeQTL(purple) and eQTL(yellow). For each step to the right on the x-axis one more covariate is added, indicated as x-axis labels. For example, the third point's p-value was calculated using the formula expr ~ grp+RIN+Sex. A) Changes in raw p-value, calculated by |pvalue1 - pvalue0|, giving more effect of pvalues in the higher p-value range. B) Performing the same comparison, but starting with p-values which had been -log10 transformed, to give more effect on changes in the lower range of p-values. Taking the median from each p-value change using C) raw p-value and D) -log10 p-value, the pattern for both variants are very similar, indicating a similar effect of each additional covariate on the whole p-value range. In general, the aeQTL analysis shows little impact of covariate usage compared to eQTL analysis.

Gene	RiskSNP	Pos	Dist (Kb)	Ref/Alt	aeQTL (est)	aeQTL txSNP (est)	aeQTL (pval)	aeQTL txSNP (pval)	txSNP	Reads	Genogroup (ind)
ALKBH5	rs8082590	chr17:18	-154	[G]/A	4.1e-05	-0.027	0.99	0.00098	rs13284	110	57/116/54
ALKBH5	rs8082590	chr17:18	-153	[G]/A	4.1e-05	0.031	0.99	0.002	rs1563371	84	57/116/54
B4GALT5	rs7267348	chr20:48	-119	T/[C]	0.012	0.1	0.0076	0.00023	rs575023	18	10/16/4
BAG5	rs12887734	chr14:104	23	G/[T]	0.016	0.046	0.0048	3.8e-07	rs7693	110	50/154/49
CDIPT	rs12691307	chr16:30	66	[A]/G	-0.029	-0.079	0.027	5.9e-05	rs11540970	19	10/20/14
CNNM2	rs11191419	chr10:105	-202	[T]/A	-0.0084	-0.036	0.26	0.001	rs2275271	87	112/55/114
CTNND1,TMX2,C11orf31	rs9420	chr11:58	-75	[A]/G	-0.007	-0.024	0.13	5.8e-08	rs708228	140	101/14/127
CTNND1,TMX2,C11orf31	rs9420	chr11:58	-76	[A]/G	-0.007	0.28	0.13	3.9e-07	rs200470100	12	7/29/16
CTNND1,TMX2,C11orf31	rs9420	chr11:58	-54	[A]/G	-0.007	-0.032	0.13	0.0024	rs10896644	34	62/113/68
CYP2D6	rs6002655	chr22:43	78	C/[T]	0.036	0.13	0.035	0.00075	rs28371704	34	22/25/16
DFNA5	chr7_24747494_D	chr7:25	-11	I/[D]	-0.028	-0.058	0.0097	0.0027	rs78941420	1900	30/206/21
DHX35	rs6065094	chr20:37	-168	A/[G]	0.0014	0.16	0.92	0.0013	rs36053162	8.7	7/10/8
GIGYF2	rs6704768	chr2:234	-120	[G]/A	-0.015	-0.085	0.028	9.2e-08	rs12328151	300	52/64/53
GOT2	rs12325245	chr16:59	-60	A/[T]	-0.0036	-0.034	0.55	5.9e-06	rs6993	310	45/221/44
HSPA9, SNORD63	rs3849046	chr5:138	-51	C/[T]	0.00087	0.018	0.72	7e-07	rs1042665	4900	86/74/75
HSPA9, SNORD63	rs3849046	chr5:138	-40	C/[T]	0.00087	-0.043	0.72	2.5e-06	rs11032	67	10/17/11
HSPA9, SNORD63	rs10043984	chr5:138	-190	C/[T]	0.005	0.017	0.085	0.00092	rs1042665	4900	51/143/41
LCAT, SLC12A4	rs8044995	chr16:68	205	G/[A]	0.033	0.035	0.00039	0.026	rs11542821	18	34/41/40
LCAT, SLC12A4	rs8044995	chr16:68	212	G/[A]	0.033	0.036	0.00039	0.066	rs1109166	3.1	15/21/15
LCAT, SLC12A4	rs8044995	chr16:68	212	G/[A]	0.033	0.052	0.00039	0.3	rs11860141	2.9	8/7/13
LCAT, SLC12A4	rs8044995	chr16:68	212	G/[A]	0.033	-0.13	0.00039	0.42	rs11860115	1.3	7/5/8
LCAT, SLC12A4	rs8044995	chr16:68	210	G/[A]	0.033	-0.025	0.00039	0.59	rs17851876	8.2	5/38/4
LCAT, SLC12A4	rs8044995	chr16:68	208	G/[A]	0.033	-0.0011	0.00039	0.96	rs11860125	7.1	31/31/23
LINC00634	rs1023500	chr22:42	-14	[T]/C	-0.013	-0.076	0.082	1e-05	rs62240997	120	49/151/46
LINC00634	rs1023500	chr22:42	-14	[T]/C	-0.013	0.03	0.082	0.0021	rs62240998	150	46/150/60
MAD1L1, MIR4655	chr7_2025096_I	chr7:2	45	D/[I]	-0.0071	-0.04	0.33	0.0011	rs10950456	41	96/22/92
MAD1L1, MIR4655	chr7_2025096_I	chr7:2	45	D/[I]	-0.0071	-0.041	0.33	0.0025	rs11557347	42	86/28/83
MAN2A2	rs4702	chr15:91	-37	[G]/A	0.0045	0.017	0.3	0.0029	rs4773	180	73/156/70
MAU2	rs2905426	chr19:19	11	[G]/T	-0.0078	-0.037	0.078	0.0022	rs756997	29	51/71/49
NCAN	rs2905426	chr19:19	116	[G]/T	0.014	0.028	0.013	8.1e-05	rs1064395	130	64/98/65
NCAN	rs2905426	chr19:19	132	[G]/T	0.014	-0.05	0.013	0.0029	rs2228600	88	16/59/21
NDRG4	rs12325245	chr16:59	141	A/[T]	0.00025	-0.032	0.95	2.5e-05	rs17821543	1800	23/7/36
NDUFA6	rs6002655	chr22:43	122	C/[T]	-0.011	-0.031	0.0083	2.8e-07	chr22_42481849	120	98/71/80
NGEF	rs6704768	chr2:234	-152	[G]/A	-0.0059	-0.024	0.023	2.4e-09	rs709937	1500	95/46/120
NGEF	rs6704768	chr2:234	-165	[G]/A	-0.0059	-0.018	0.023	4e-05	rs748002	1600	104/56/117
NSUN6	rs7893279	chr10:19	-90	[T]/G	0.048	0.11	0.0032	1.7e-05	rs373525672	10	23/58/11
PLCB2	rs56205728	chr15:41	-16	G/[A]	-0.015	-0.11	0.34	0.00027	rs62021888	3	21/4/13
PPP1R13B	rs12887734	chr14:104	-158	G/[T]	0.036	0.035	6e-04	0.016	rs17292804	23	30/14/25
PPP1R13B	rs12887734	chr14:104	-154	G/[T]	0.036	0.016	6e-04	0.58	rs10134157	40	8/48/6
PPP1R16B	rs6065094	chr20:37	-95	A/[G]	0.0029	0.058	0.51	0.0013	rs45562136	31	11/27/8
PRMT7	rs8044995	chr16:68	-156	G/[A]	-0.029	-0.072	0.00081	0.005	rs3743741	85	34/119/40
PRMT7	rs8044995	chr16:68	-201	G/[A]	-0.029	-0.035	0.00081	0.035	rs61733486	32	21/23/27
PRMT7	rs8044995	chr16:68	-198	G/[A]	-0.029	-0.021	0.00081	0.095	rs61746794	340	43/55/40
PRMT7	rs8044995	chr16:68	-200	G/[A]	-0.029	-0.0082	0.00081	0.38	rs1131933	530	48/207/64
PRMT7	rs8044995	chr16:68	-191	G/[A]	-0.029	-0.0065	0.00081	0.96	rs75567372	5.7	2/12/6
PRMT7	rs8044995	chr16:68	-184	G/[A]	-0.029	-0.29	0.00081	NaN	rs138720818	2.9	3/5/5
PRRT2	rs12691307	chr16:30	114	[A]/G	-0.0035	-0.087	0.3	0.00061	rs141436390	31	7/7/6
R3HDM2	rs324017	chr12:57	-186	A/[C]	-0.01	0.1	0.11	0.0017	rs77784795	56	7/13/8
RCN3	rs56873913	chr19:50	45	[T]/G	-0.025	-0.075	0.088	1e-04	rs3187346	50	40/49/34
REEP2	rs10043984	chr5:138	-70	C/[T]	-0.00031	-0.0099	0.89	0.00086	rs1046576	470	109/118/117
RERE	chr1_8424984_D	chr1:8	12	[I]/D	-0.0048	-0.04	0.15	2.9e-11	rs1064826	250	74/28/107
RERE	chr1_8424984_D	chr1:8	12	[I]/D	-0.0048	-0.026	0.15	9.2e-08	rs8627	420	79/136/99
RERE	chr1_8424984_D	chr1:8	6	[I]/D	-0.0048	0.026	0.15	8.1e-05	rs11121172	240	87/22/121
RERE	chr1_8424984_D	chr1:8	6	[I]/D	-0.0048	0.021	0.15	0.0018	rs1058766	240	86/19/121
RFTN2	rs6434928	chr2:198	-132	[G]/A	-0.021	-0.03	0.011	2.5e-05	rs7582536	43	120/3/104
RPS11, SNORD35B	rs56873913	chr19:50	92	[T]/G	-0.0051	-0.071	0.7	0.0014	rs17555056	26	30/56/55
SDCCAG8, MIR4677	rs10803138	chr1:244	-24	A/[G]	-0.024	-0.059	0.033	5.3e-05	rs10927011	40	64/130/48
SEZ6L2	rs12691307	chr16:30	29	[A]/G	0.015	0.033	0.032	4e-04	rs11344	160	67/143/74
SMG6	rs4523957	chr17:2	23	G/[T]	0.0026	0.037	0.62	5.3e-06	rs749240	23	112/17/123
SMG6	rs4523957	chr17:2	6	G/[T]	0.0026	-0.042	0.62	0.0012	rs1885987	27	53/82/68

Gene	RiskSNP	Pos	Dist (Kb)	Ref/Alt	aeQTL (est)	aeQTL txSNP (est)	aeQTL (pval)	aeQTL txSNP (pval)	txSNP	Reads	Genogroup (ind)
SOX2, SOX2-OT	rs9841616	chr3:181	-291	[T]/A	-0.004	0.039	0.49	0.0017	rs73182316	610	39/188/49
STAT6	rs324017	chr12:57	-2	A/[C]	0.0074	0.11	0.25	8.8e-26	rs4559	180	94/141/89
STAT6	rs324017	chr12:57	-2	A/[C]	0.0074	-0.055	0.25	1e-13	rs324015	54	104/18/108
STAT6	rs324017	chr12:57	-2	A/[C]	0.0074	-0.032	0.25	0.00011	rs703817	83	67/148/66
STAT6	rs324017	chr12:57	-2	A/[C]	0.0074	0.079	0.25	0.00011	rs1059513	35	18/60/16
SUGP1	rs2905426	chr19:19	88	[G]/T	-0.00062	-0.03	0.93	0.00045	rs11555053	240	60/86/57
TAOK2	rs12691307	chr16:30	-55	[A]/G	0.001	-0.03	0.81	0.00011	rs3814883	280	95/56/112
TCF20	rs6002655	chr22:43	-6	C/[T]	-0.012	-0.1	0.13	0.0012	rs34030679	2.8	9/18/10
TMEM110, MUSTN1,TMEM110-MUSTN1	rs2535627	chr3:53	-23	[T]/C	-0.012	0.11	0.05	0.0013	rs6787916	78	13/27/9
VPS45	rs140505938	chr1:150	-8	[C]/T	0.056	0.062	0.0018	0.0037	rs2027349	5.2	18/39/21
WBP1L	rs11191419	chr10:105	38	[T]/A	-0.002	-0.034	0.63	0.00019	rs284858	46	103/110/105
WDR55	chr5_140143664_I	chr5:140	95	D/[I]	-0.0094	-0.1	0.14	0.00019	rs35983033	9.6	31/26/11

Table S1-3: For DLPFC, similar to Table 1 in the main manuscript, but for individual txSNPs that were FDR5% signif cant

Gene	RiskSNP	Pos	Dist (Kb)	Ref/Alt	aeQTL (est)	aeQTL txSNP (est)	aeQTL (pval)	aeQTL txSNP (pval)	txSNP	Reads	Genogroup (ind)
AS3MT, BORCS7	rs55833108	chr10:105	118	G/[T]	-0.032	-0.13	0.14	0.00027	rs9527	35	10/95/9
ATXN7	rs832187	chr3:64	-155	[C]/T	-0.004	-0.082	0.69	0.00056	rs3733126	54	40/45/27
BAG5	rs12887734	chr14:104	23	G/[T]	0.013	0.077	0.3	0.0017	rs7693	46	21/73/25
CTNND1,TMX2,C11orf31	rs9420	chr11:58	-19	[A]/G	0.0083	0.088	0.28	3e-04	rs11570176	210	29/64/19
DFNA5	chr7_24747494_D	chr7:25	9	I/[D]	-0.056	-0.089	0.029	0.0026	rs12979	39	22/43/18
FAM53C	rs10043984	chr5:138	27	C/[T]	0.022	0.077	0.056	0.0014	rs1052648	37	27/74/21
HAPLN4	rs2905426	chr19:19	111	[G]/T	0.038	0.093	0.022	0.0021	rs55762233	39	20/45/24
HSPA9, SNORD63	rs10043984	chr5:138	-190	C/[T]	0.0047	0.042	0.42	2.9e-05	rs1042665	4800	33/88/29
HSPA9, SNORD63	rs3849046	chr5:138	-51	C/[T]	0.0022	0.031	0.68	8.1e-05	rs1042665	4800	48/55/47
HSPA9, SNORD63	rs3849046	chr5:138	-41	C/[T]	0.0022	-0.022	0.68	0.00066	rs10117	380	61/52/60
KLC1	rs12887734	chr14:104	-49	G/[T]	-0.0028	0.19	0.8	0.00085	rs1054080	27	7/53/10
LRP1	rs12826178	chr12:58	33	[G]/T	0.02	0.17	0.22	0.0016	rs1800154	110	5/180/5
PTPRF	rs11210892	chr1:44	13	[G]/A	0.0011	-0.073	0.9	1.7e-05	rs1143702	160	57/63/60
RERE	chr1_8424984_D	chr1:8	12	[I]/D	0.0049	-0.053	0.55	0.0012	rs1064826	68	42/21/55
SDCCAG8, MIR4677	rs10803138	chr1:244	61	A/[G]	-0.042	-0.11	0.0032	0.00071	rs2275155	760	24/30/26
SEPT3	rs1023500	chr22:42	-52	[T]/C	0.003	0.067	0.65	0.0027	rs710194	190	7/51/10
SF3B1	rs6434928	chr2:198	47	[G]/A	-0.0083	-0.027	0.13	0.0015	rs4685	170	71/1/70
SOX2, SOX2-OT	rs9841616	chr3:181	-291	[T]/A	-0.007	0.24	0.45	0.0012	rs7616347	89	5/12/7
SREBF2, MIR33A	rs1023500	chr22:42	64	[T]/C	-0.00057	-0.11	0.95	0.00038	rs2228314	2900	14/53/15
STAT6	rs324017	chr12:57	-2	A/[C]	-0.028	-0.055	0.038	0.0026	rs324015	19	36/5/36
TMEM110, MUSTN1,TMEM110-MUSTN1	rs2535627	chr3:53	-27	[T]/C	-0.0055	-0.12	0.64	0.0016	rs2878634	13	14/27/19
TOM1L2	rs8082590	chr17:18	210	[G]/A	-0.011	-0.043	0.026	0.0018	rs4924822	66	68/43/74

Table S1-4: For Hippocampus, similar to Table 1 in the main manuscript, but for individual txSNPs that were FDR5% signif cant

Gene	SNP	Pos	Dist (Kb)	Dist Rank	Ref/Alt	Direction	aeQTL p-value	txSNPs	Reads	Individuals per group	Support
AS3MT, BORCS7	rs11191419	chr10:105	-2	1	[T]/A	up	3.1e-21	20	150	159/141/152	Ge,Ga,Re,Je,Js
APOPT1	rs12887734	chr14:104	0	1	G/[T]	down	2e-44	8	6300	126/64/116	Ge,Ga,Je,Js
ZMAT2	chr5_140143664_I	chr5:140	58	2	D/[I]	down	4e-22	2	250	162/36/143	Ga
HAPLN4	rs2905426	chr19:19	109	5	[G]/T	up	3.8e-19	12	230	103/163/106	Ga
CD46	rs7523273	chr1:208	8	1	[A]/G	up	5.4e-17	9	250	147/121/126	Ge,Ga,Je,Js
BRINP2	rs6670165	chr1:177	29	1	C/[T]	down	1e-12	4	1600	84/272/93	Ga,Js
NDUFA2	chr5_140143664_I	chr5:140	116	8	D/[I]	down	3e-12	2	77	134/85/107	Ga,Re
TOM1L2	rs8082590	chr17:18	186	8	[G]/A	down	3.8e-12	24	360	140/154/162	Ga,Js
FURIN	rs4702	chr15:91	0	1	[G]/A	down	5.3e-12	10	61	142/226/144	Ga,Fe
ANP32E	rs140505938	chr1:150	-160	8	[C]/T	down	4.4e-10	8	680	49/342/56	Ga
CKB	rs12887734	chr14:104	59	4	G/[T]	down	7.7e-09	3	8900	70/268/71	Ga
SPCS1	rs2535627	chr3:53	103	6	[T]/C	down	8e-09	3	240	120/135/104	Ga
SNX19	rs10791097	chr11:131	-28	1	[T]/G	up	1.6e-08	44	710	160/319/145	Ga,Je,Js
PTPRF	rs11210892	chr1:44	12	1	[G]/A	down	2.5e-07	17	1600	140/311/131	Ga
NEK4	rs2535627	chr3:53	40	4	[T]/C	up	4.8e-07	9	44	140/170/141	Ga,Je,Js
FAM57B	rs12691307	chr16:30	-96	12	[A]/G	up	6.6e-07	4	370	139/167/152	Ga,Js
LRP1	rs12826178	chr12:58	16	4	[G]/T	down	5.9e-06	32	150	25/455/23	Ga
HSPE1, MOB4	rs6434928	chr2:198	-60	4	[G]/A	up	3.5e-05	9	51	127/149/119	Ga
HSPD1	rs6434928	chr2:198	-47	3	[G]/A	down	3.9e-05	6	280	142/211/125	Ga,Js
VPS45	rs140505938	chr1:150	-8	1	[C]/T	up	4e-05	5	120	20/90/24	Ga,Js
PRMT7	rs8044995	chr16:68	-156	7	G/[A]	down	0.00027	9	520	86/288/103	Ge,Ga,Re,Je,Js
ZFYVE21	rs12887734	chr14:104	-146	7	G/[T]	down	0.00034	7	420	118/255/103	Ga
FAM53C	rs10043984	chr5:138	27	2	C/[T]	down	0.00035	9	100	104/260/89	Ga,Js
AS3MT, BORCS7	rs55833108	chr10:105	80	2	G/[T]	down	6e-04	20	150	39/369/44	Ge,Ga
IK, MIR3655	chr5_140143664_I	chr5:140	102	7	D/[I]	down	0.00066	5	160	96/130/81	Ga
GNL3, SNORD19,SNORD69,SNORD19B	rs2535627	chr3:53	117	8	[T]/C	up	7e-04	9	410	153/167/144	Re
PPP1R13B	rs12887734	chr14:104	-153	8	G/[T]	down	0.001	9	42	43/119/39	Ga
RIMS1	rs1339227	chr6:73	43	1	[C]/T	down	0.0012	21	420	130/365/108	Ga
DNAJC19	chr3_180594593_I	chr3:181	-107	2	[D]/I	up	0.0014	2	54	126/19/132	Ga
CDIPT	rs12691307	chr16:30	66	7	[A]/G	down	0.0016	3	31	27/63/24	Ga
GATAD2A	rs2905426	chr19:19	-98	4	[G]/T	down	0.0016	15	85	135/162/144	Ga,Je,Js
CUL3	rs11685299	chr2:225	0	1	[C]/A	up	0.0018	24	820	96/217/96	Ga,Re
NSUN6	rs7893279	chr10:19	-90	2	[T]/G	up	0.0023	2	23	39/241/31	Ga,Js

Table S1-5: For DLPFC, similar to Table 1 in the main manuscript, but here all fetal samples were used

Gene	SNP	Pos	Dist (Kb)	Dist Rank	Ref/Alt	Direction	aeQTL p-value	txSNPs	Reads	Individuals per group	Support
AS3MT, BORCS7	rs11191419	chr10:105	-2	1	[T]/A	up	2.2e-08	20	120	90/58/88	Ge,Ga,Re,Je,Js
ZMAT2	chr5_140143664_I	chr5:140	58	2	D/[I]	down	3.9e-09	2	180	97/34/78	Ga
SNX19	rs10791097	chr11:131	-28	1	[T]/G	up	2e-07	44	970	98/212/90	Ga,Je,Js
HSPD1	rs6434928	chr2:198	-47	3	[G]/A	down	6.3e-06	6	380	77/157/71	Ga,Js
DNAJC19	chr3_180594593_I	chr3:181	-107	2	[D]/I	up	0.0015	2	37	79/21/64	Ga
SDCCAG8	rs10803138	chr1:244	0	1	A/[G]	down	0.0022	4	730	54/148/54	Ga

Table S1-6: For Hippocampus, similar to Table 1 in the main manuscript, but here all fetal samples were used
