# Supplementary material for: Comparison of quantitative trait loci methods: Total expression and allelic imbalance method in brain RNA-seq
Source: PLoS One. 2019 Jun 17;14(6):e0217765. doi: 10.1371/journal.pone.0217765 (PMC6576752; doi:10.1371/journal.pone.0217765)
Supplement: S2 File — The File contains the supplemental figures S2-1 to S2-34. (PDF) [file pone.0217765.s002.pdf]

Allelic Imbalance in SZ (Supplemental Material 2)

This is a document containing supplemental figures for the "Comparison of Quantitative Trait Loci Methods: Total Expression and Allelic Imbalance Method in Brain RNA-seq" manuscript. On each page are each gene-snp pair in table 1 in the main manuscript additionally described by graphs and auxillary tests. **A**) Similar to the plot 1B, which is described in the main manuscript. **B**) A regression test of the reference fraction for each genotype group to prove that there is no underlying skew in the distribution of ref/alt alleles, which would bias the aeQTL result. As can be seen in some graphs there is an underlying mapbias where the reference allele has a higher fraction in all genotype groups, but important is that the bias is equally present in each group, and therefore not affecting the aeQTL analysis. **C**) Illustration of the distribution between homozygotes and homozygotes together with a p-value for a one sided variance test as used by Fogarthy et al. The idea is that the heterozygote group should have a higher variance as it is assumed that all fractions from the homozygote genotype groups are supposed to lie close to 0.50 and the heterozygote group to have a deviation from 0.50 and thereby create a higher variance in the heterozygote group. This approach was largely unsuccessful, as no test showed significance, even though we know that we have several aeQTLs present from our regression analysis. **D**) A QQ-plot for each genotype group's parent-1 allele fraction value to its theoretical normal distribution quantiles. The grey shading indicates the confidence bands at alpha=0.05. **E**) A residuals versus fitted values plot based on the aeQTL test on parent-1 allele fraction, to inspect heteroscedasticity/homoscedasticity.

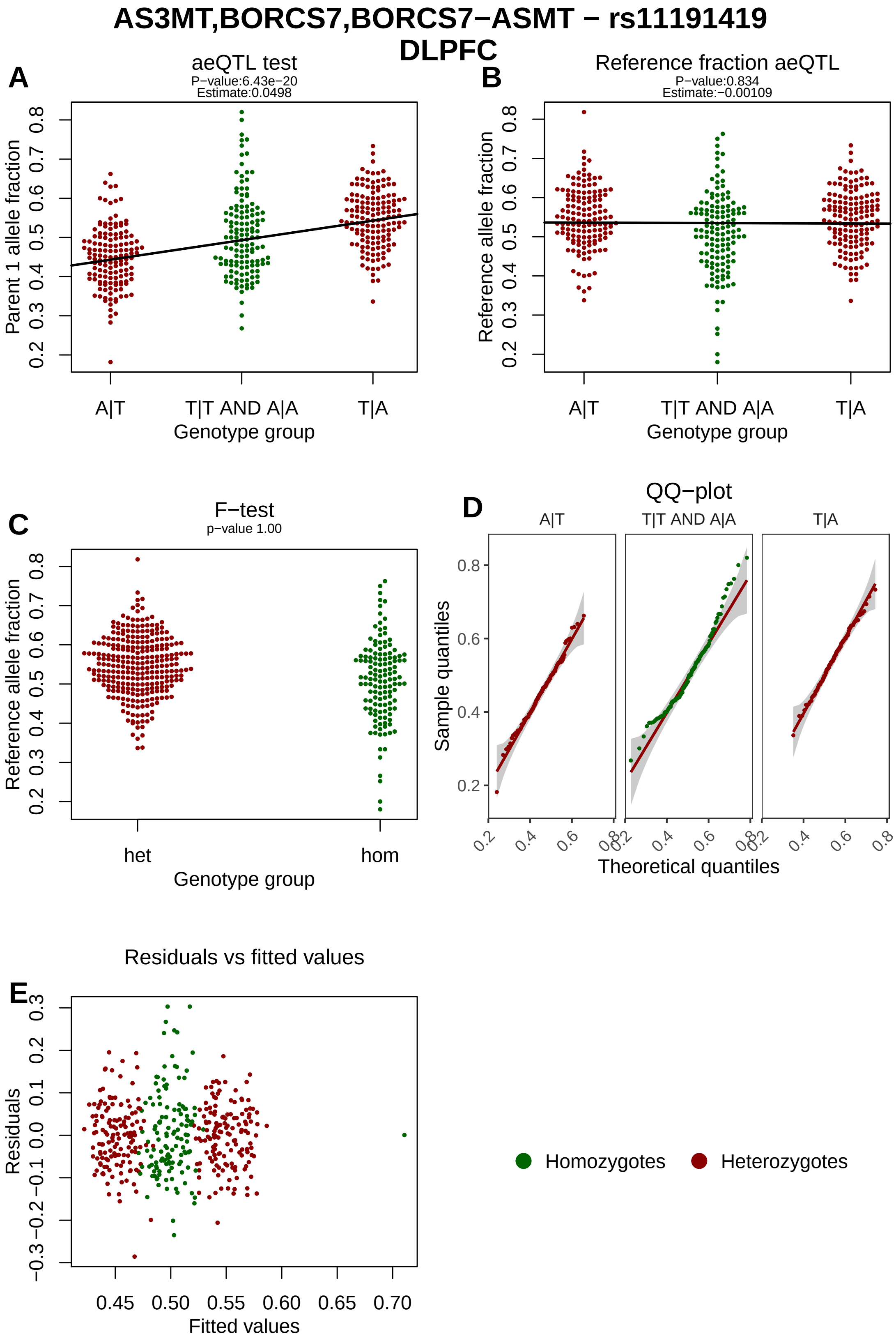

Figure S2−1: More details in the header of this document.

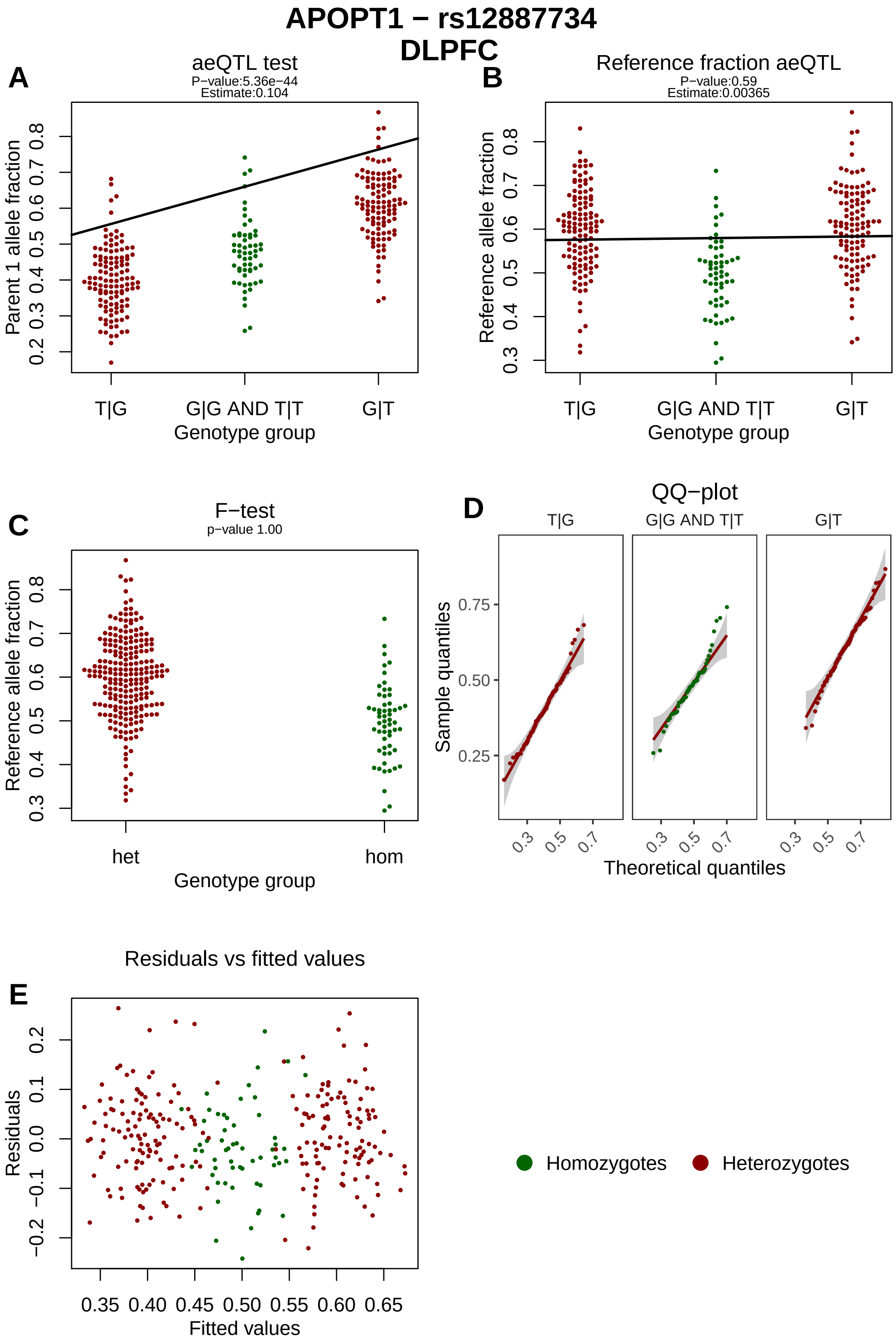

Figure S2−2: More details in the header of this document.

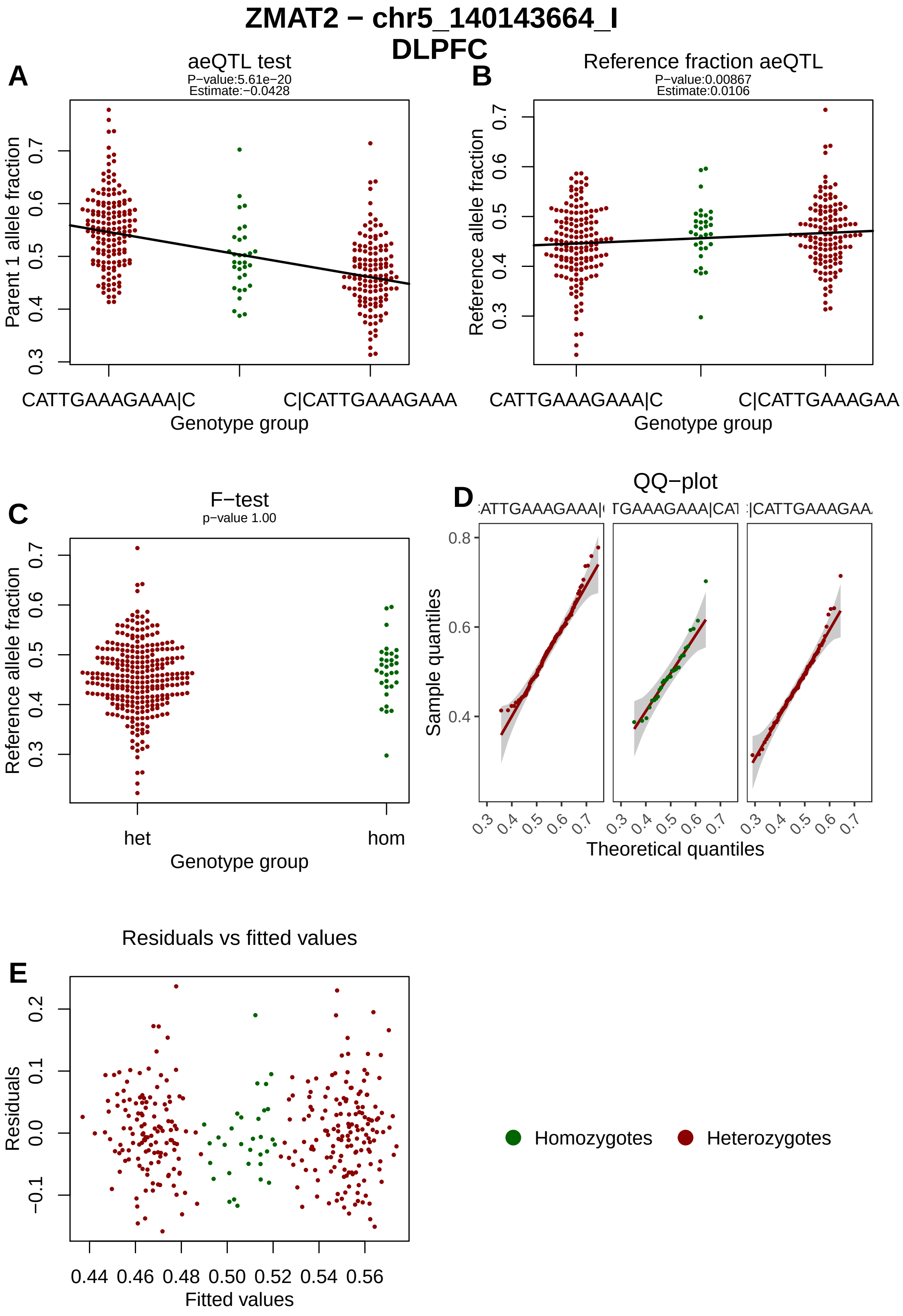

Figure S2−3: More details in the header of this document.

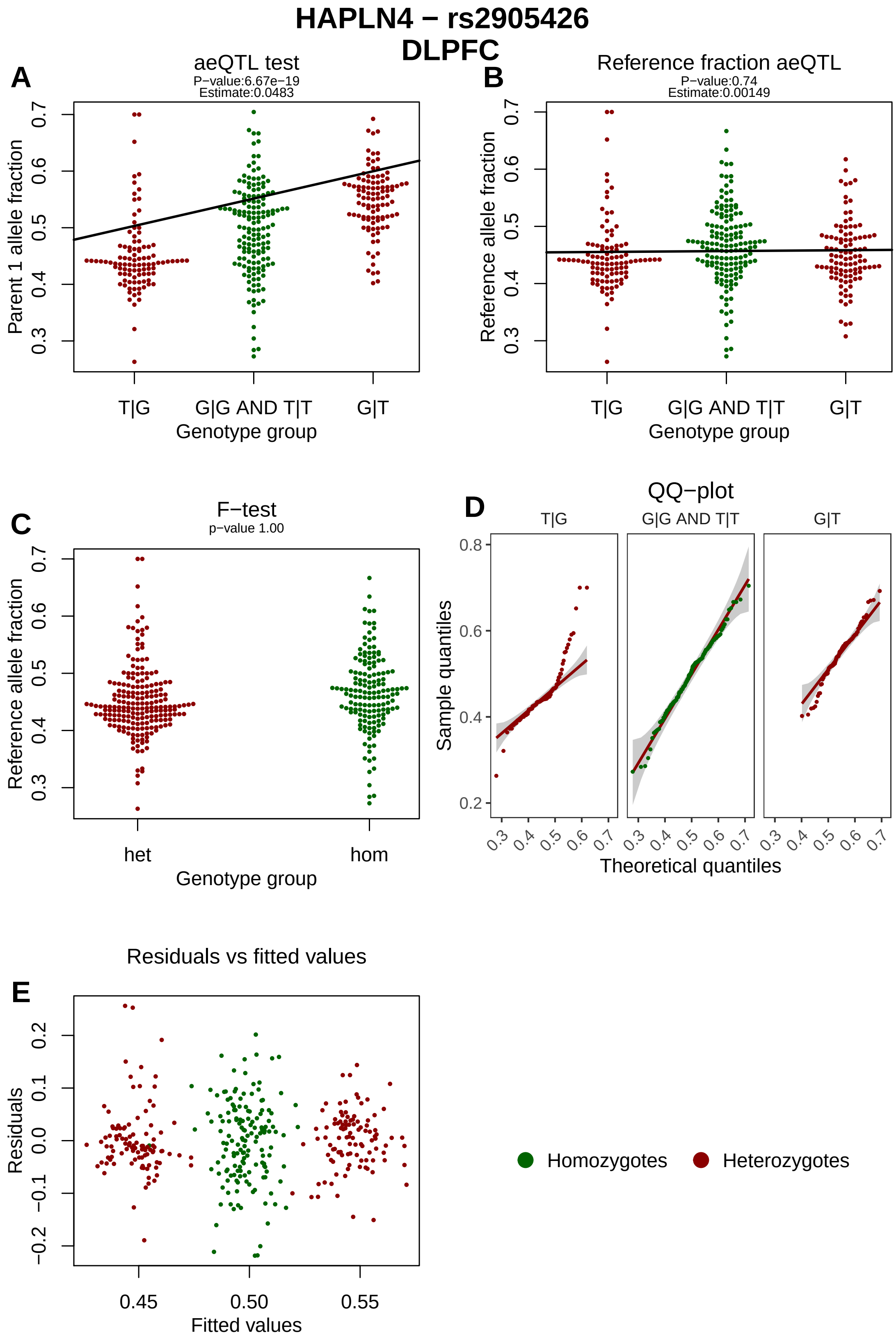

Figure S2−4: More details in the header of this document.

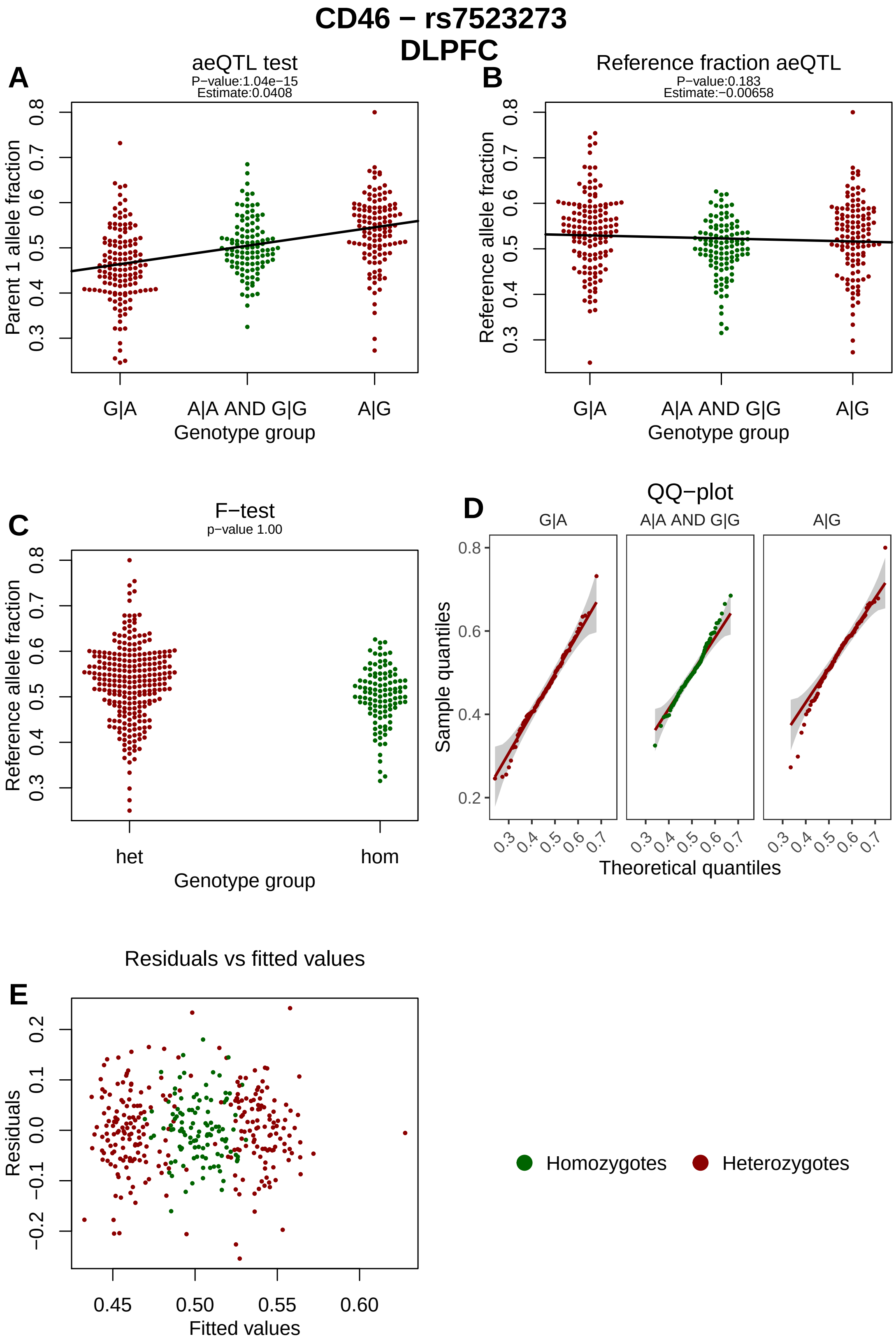

Figure S2−5: More details in the header of this document.

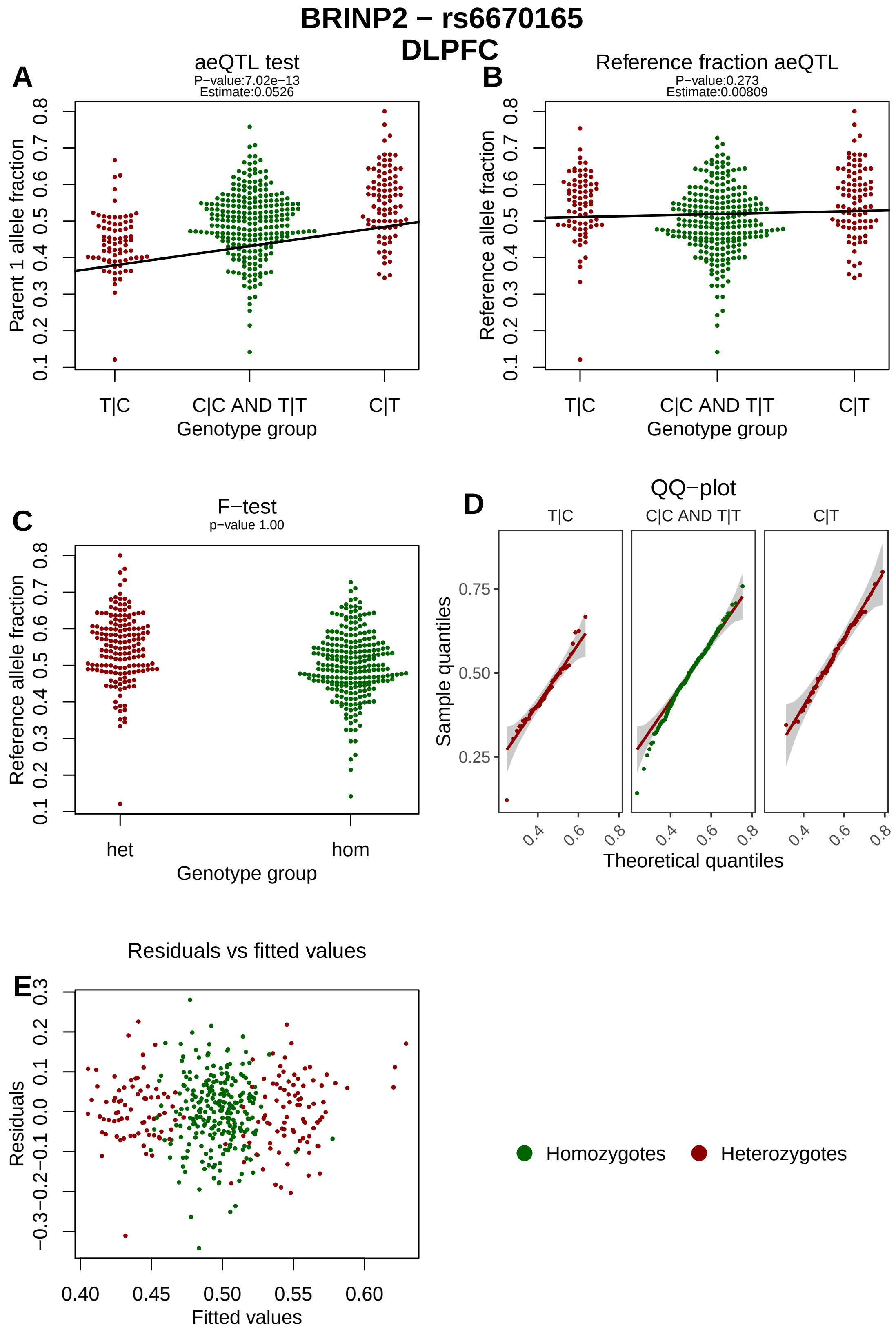

Figure S2−6: More details in the header of this document.

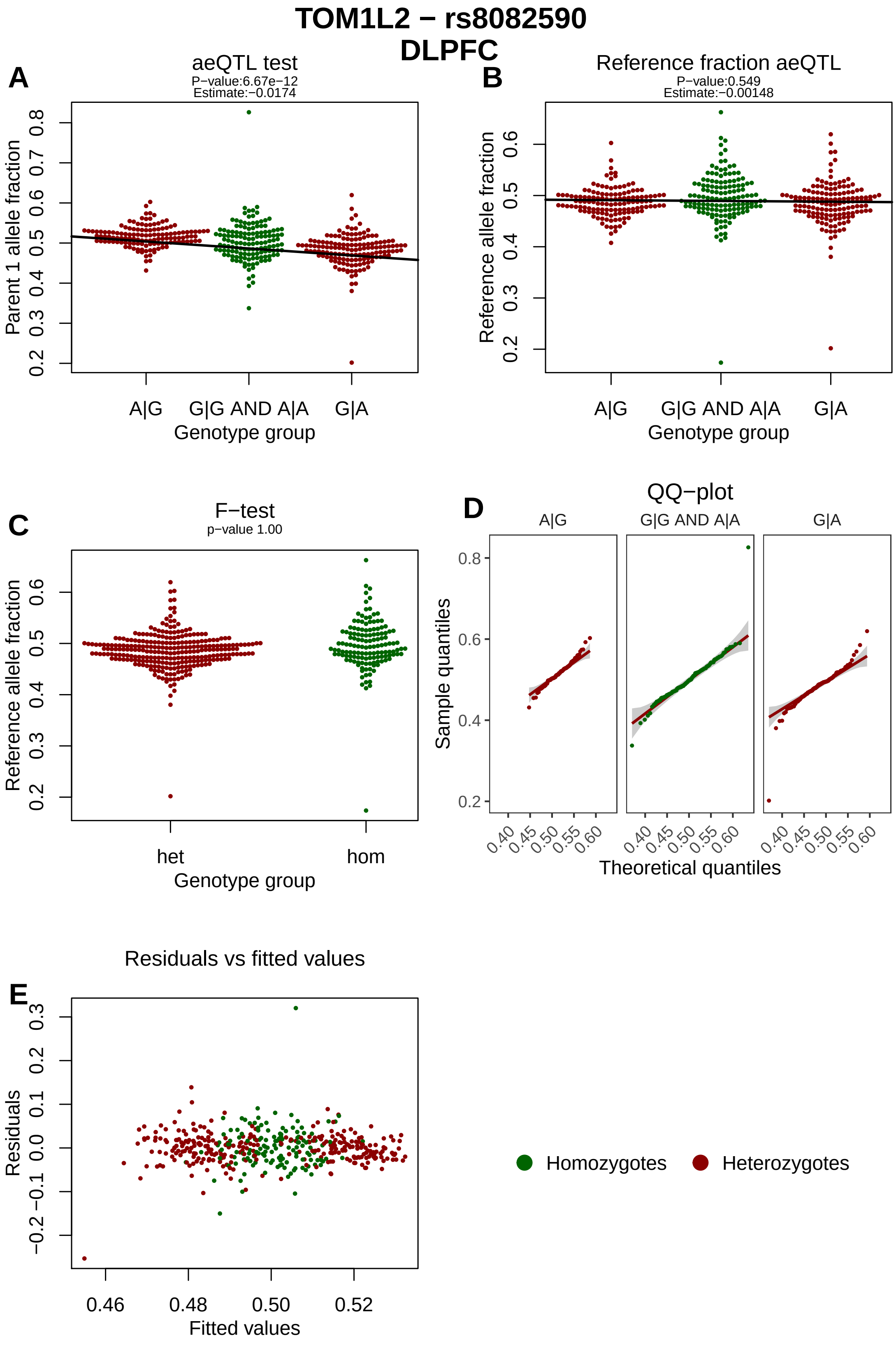

Figure S2−7: More details in the header of this document.

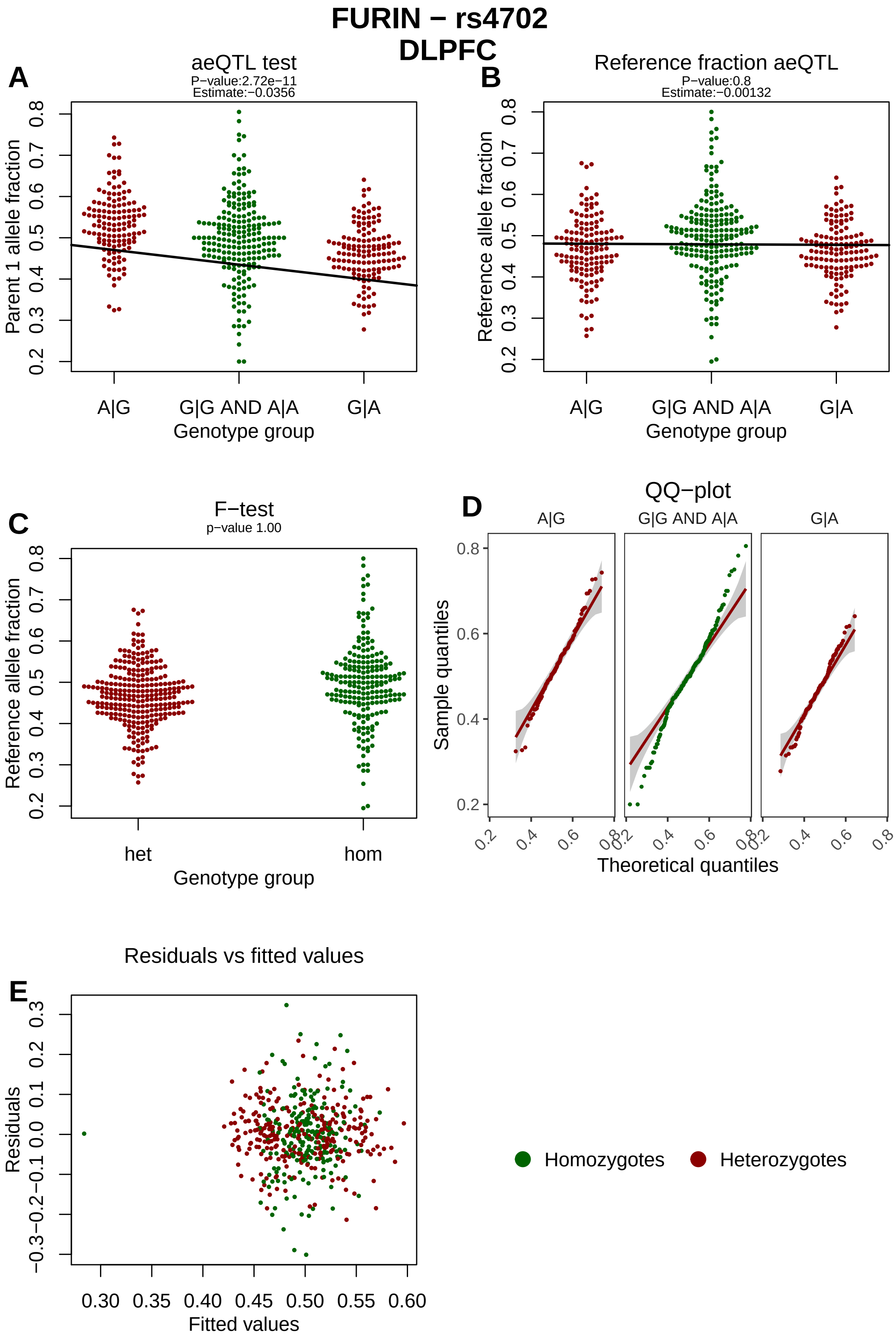

Figure S2−8: More details in the header of this document.

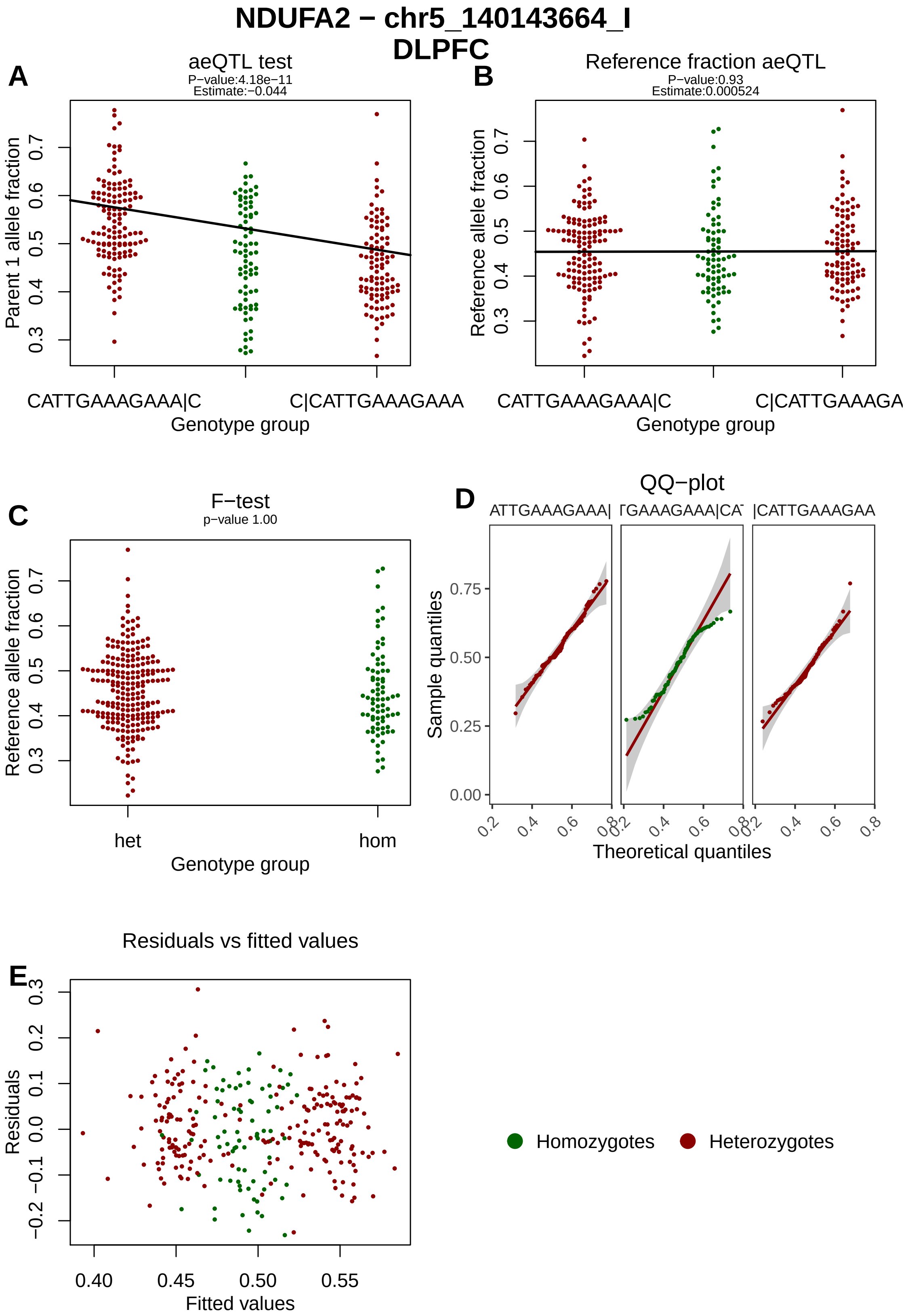

Figure S2−9: More details in the header of this document.

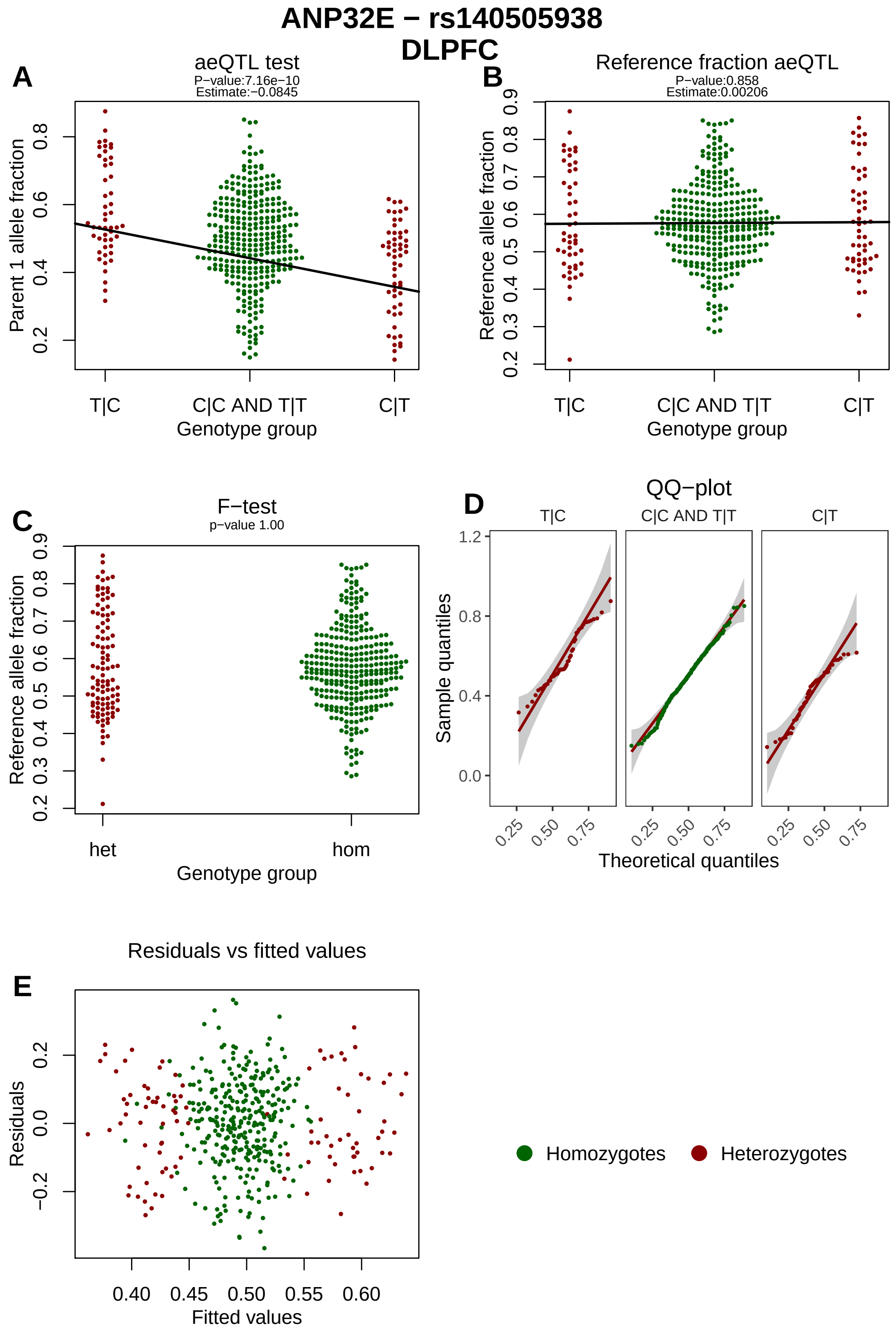

Figure S2−10: More details in the header of this document.

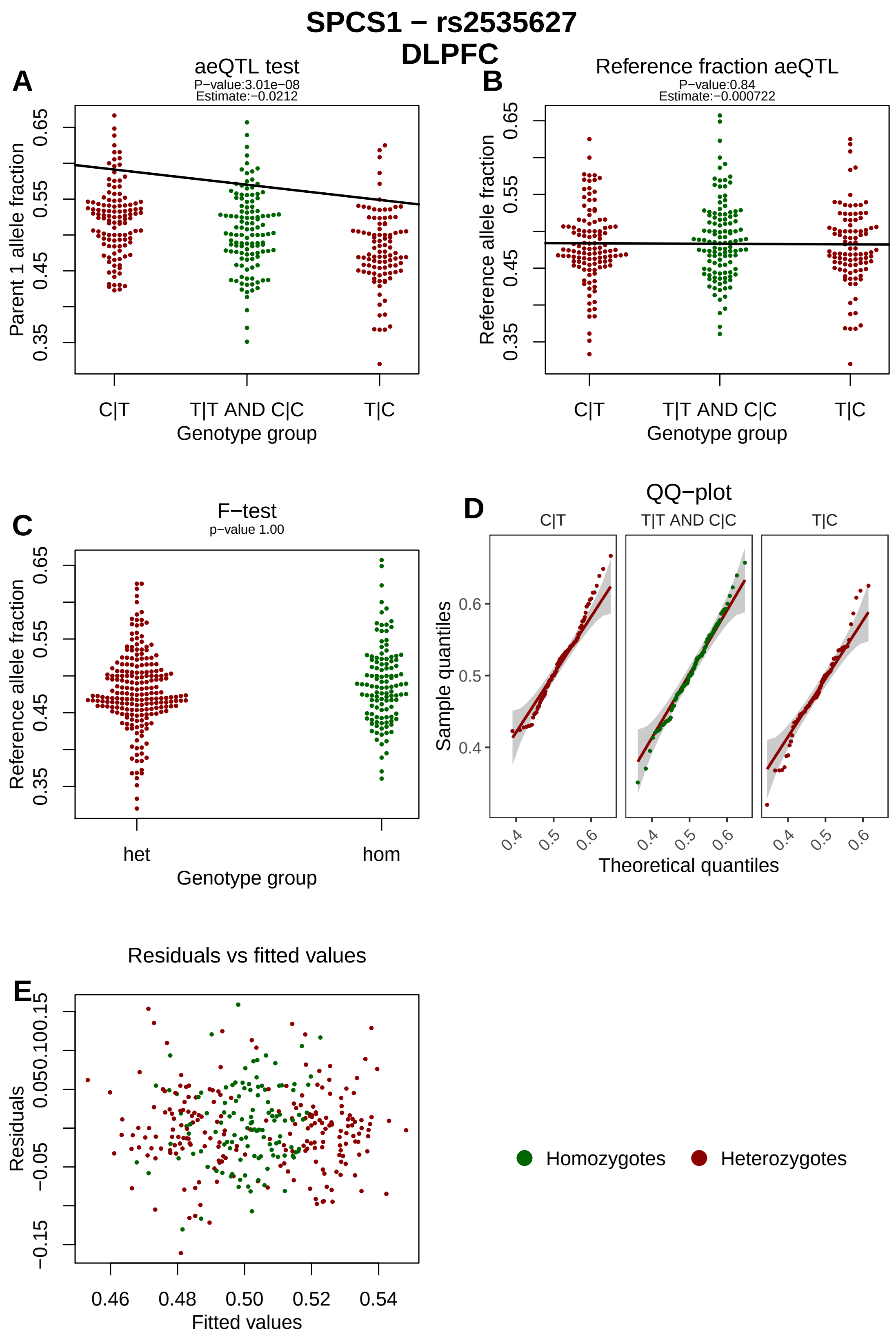

Figure S2−11: More details in the header of this document.

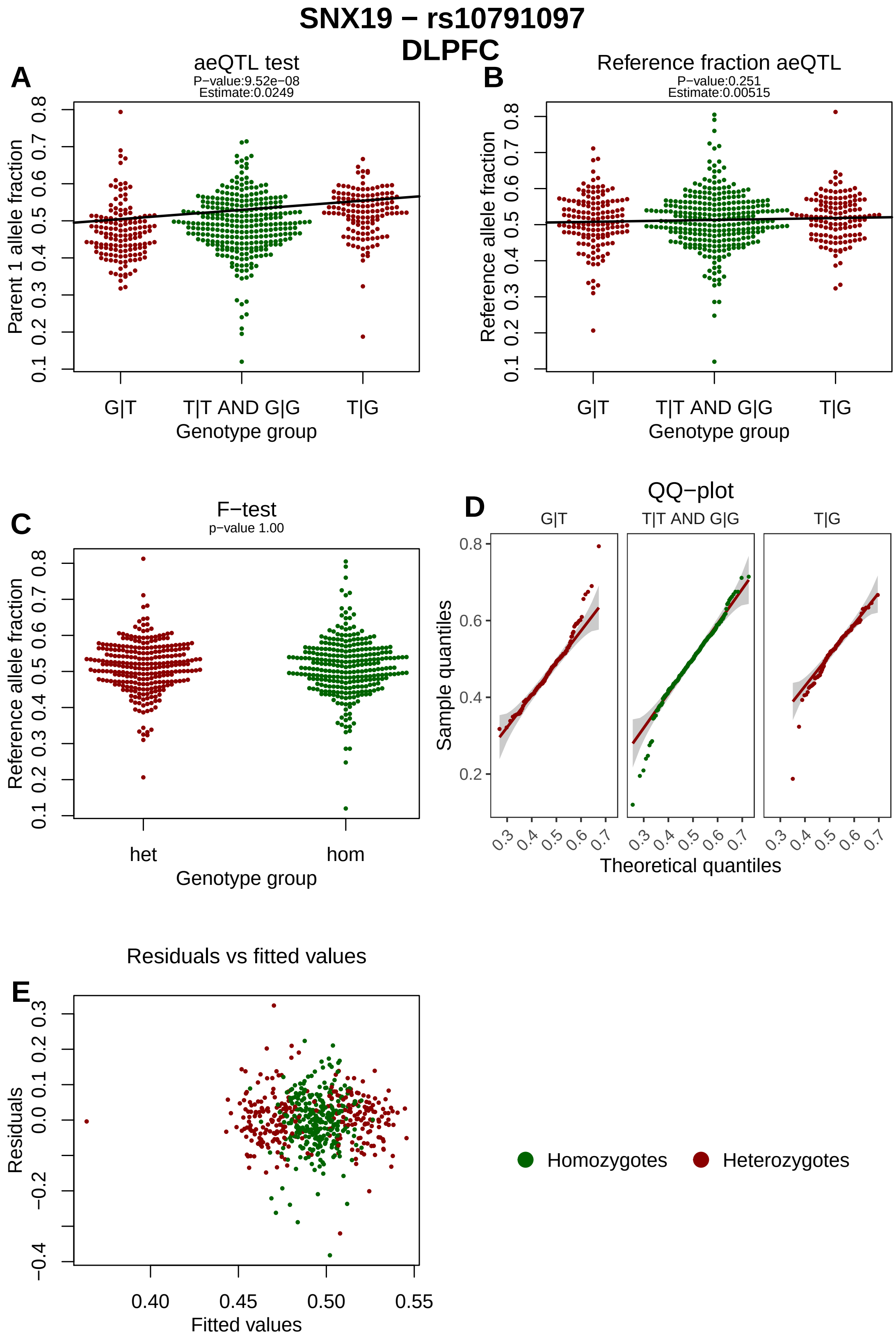

Figure S2−12: More details in the header of this document.

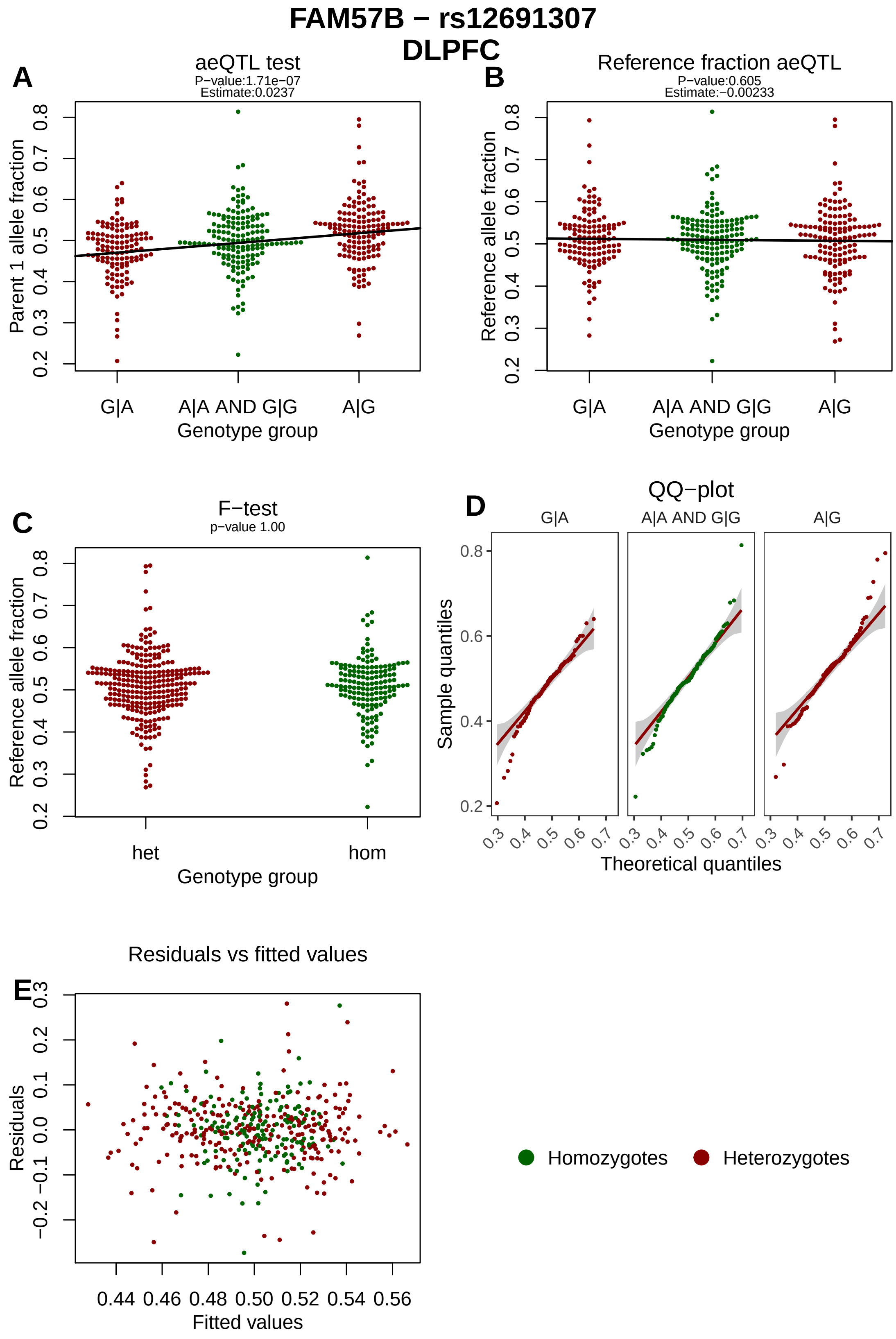

Figure S2−13: More details in the header of this document.

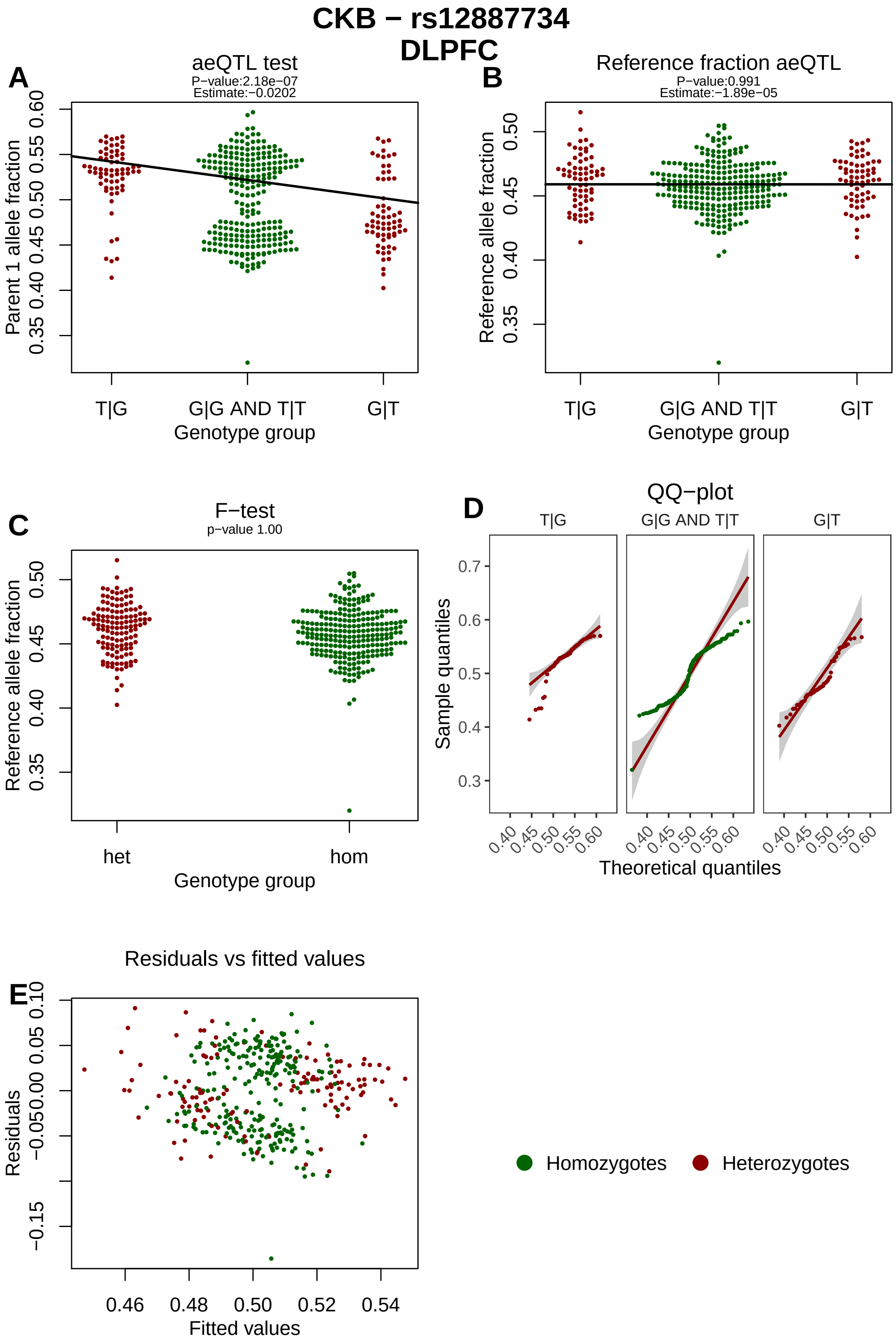

Figure S2−14: More details in the header of this document.

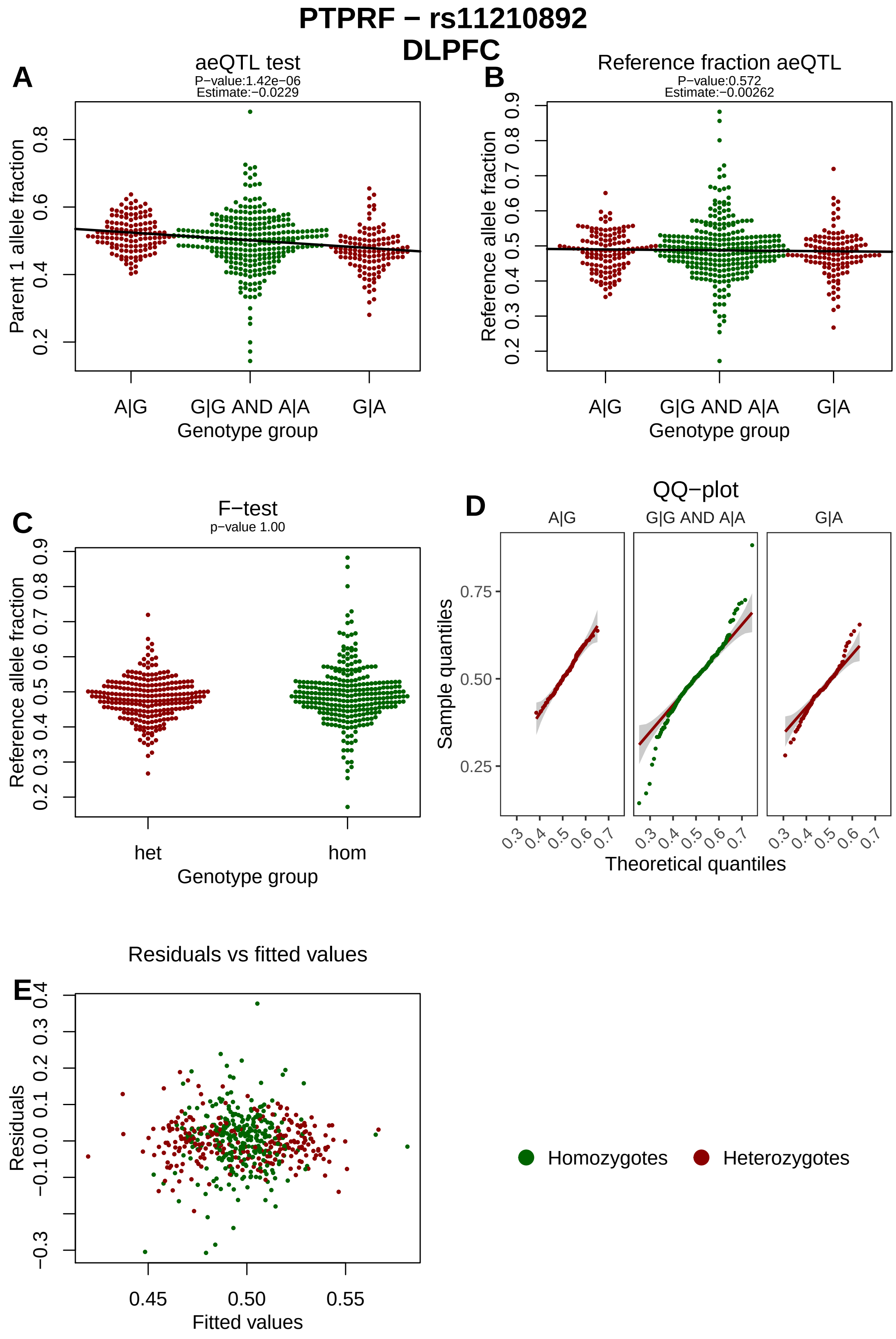

Figure S2−15: More details in the header of this document.

HSPE1,MOB4,HSPE1−MOB4 − rs6434928
DLPFC

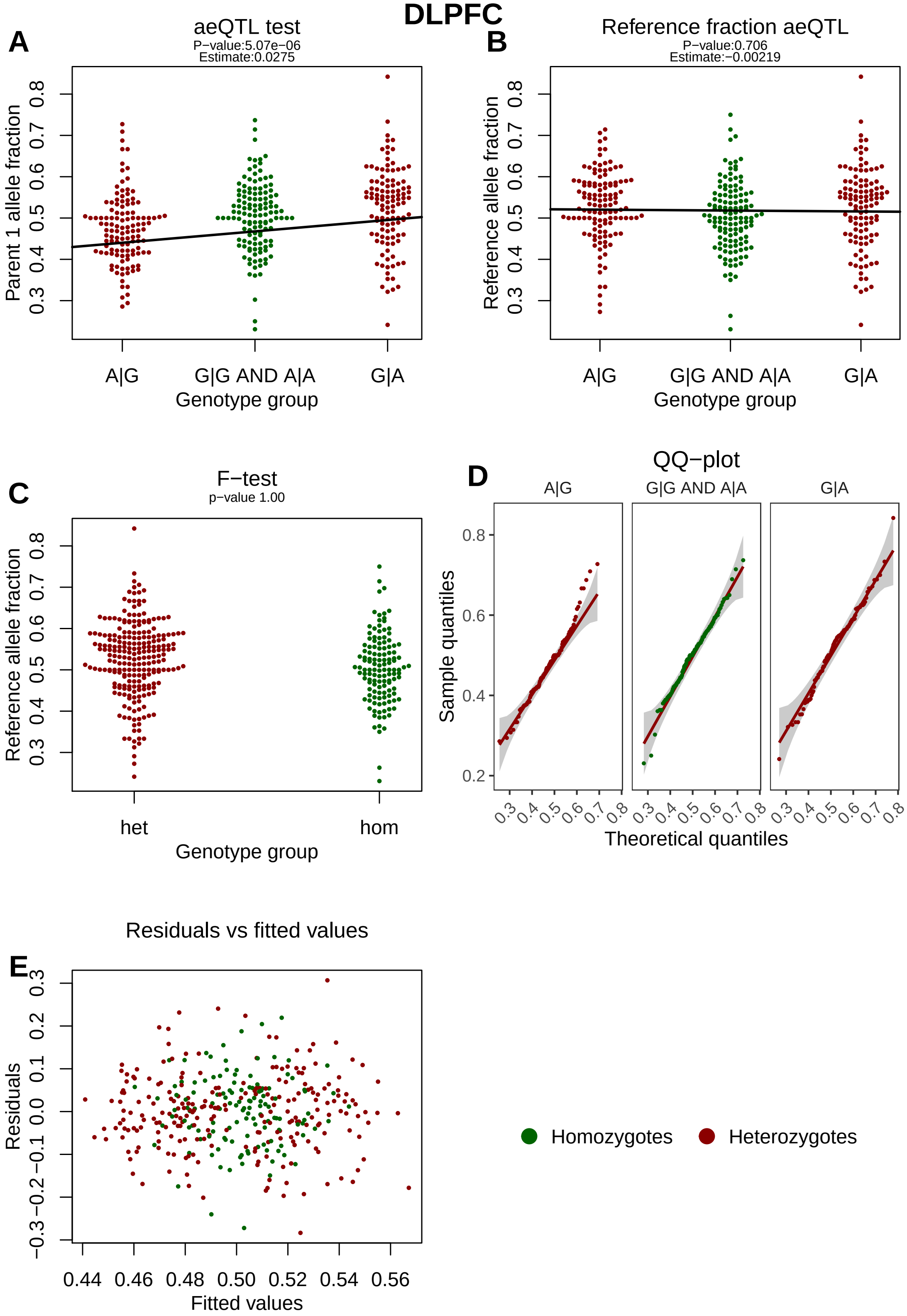

Figure S2−16: More details in the header of this document.

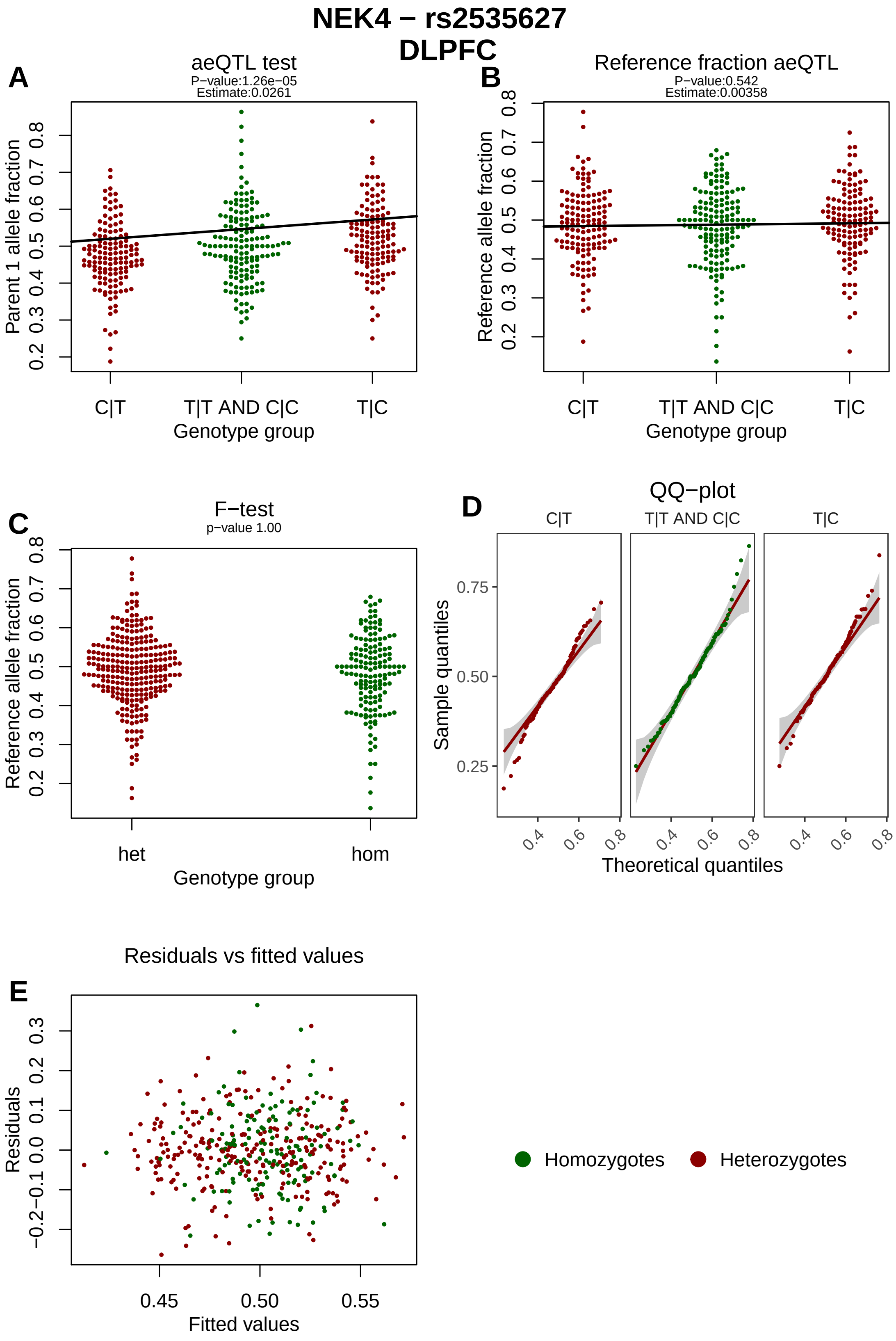

Figure S2−17: More details in the header of this document.

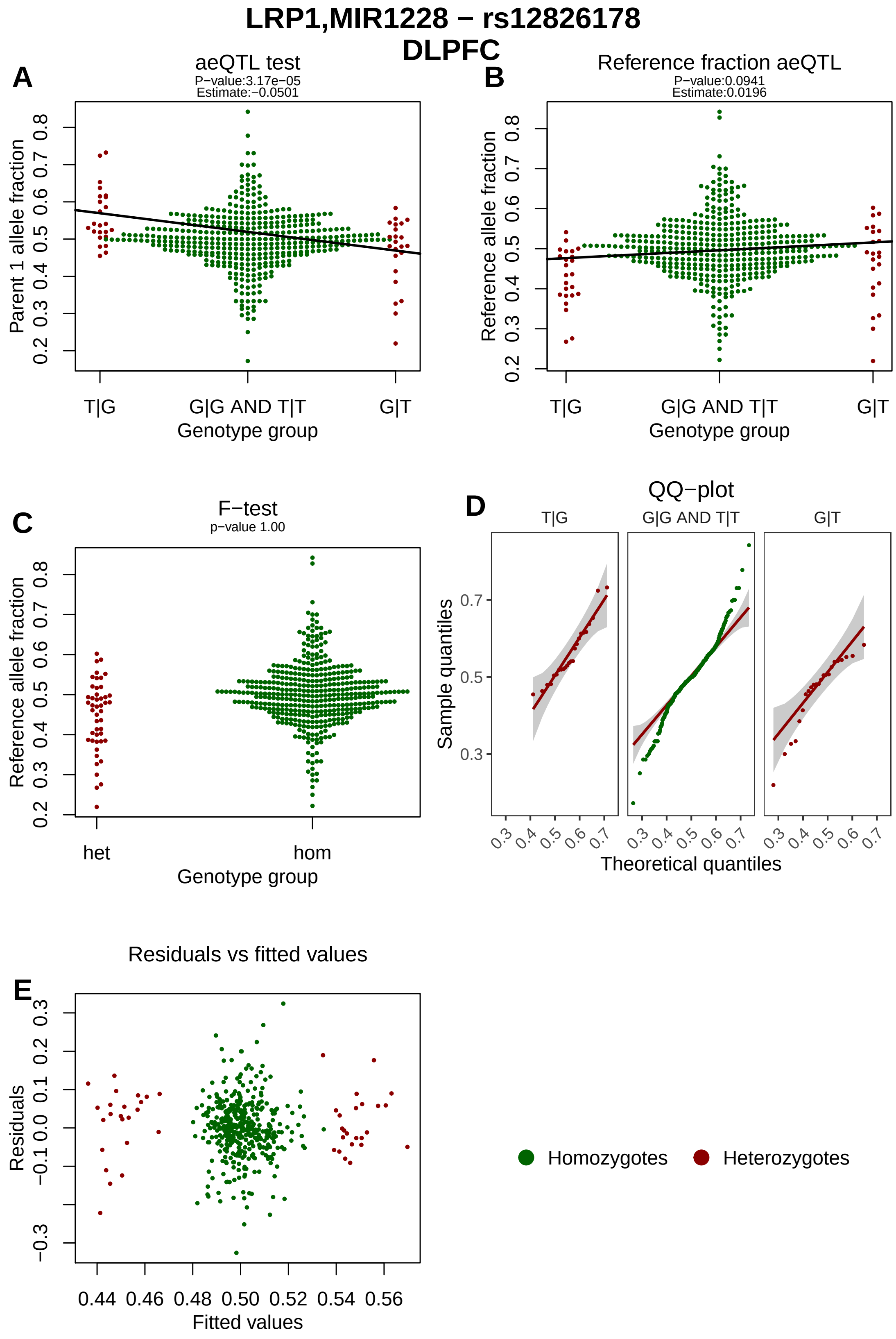

Figure S2−18: More details in the header of this document.

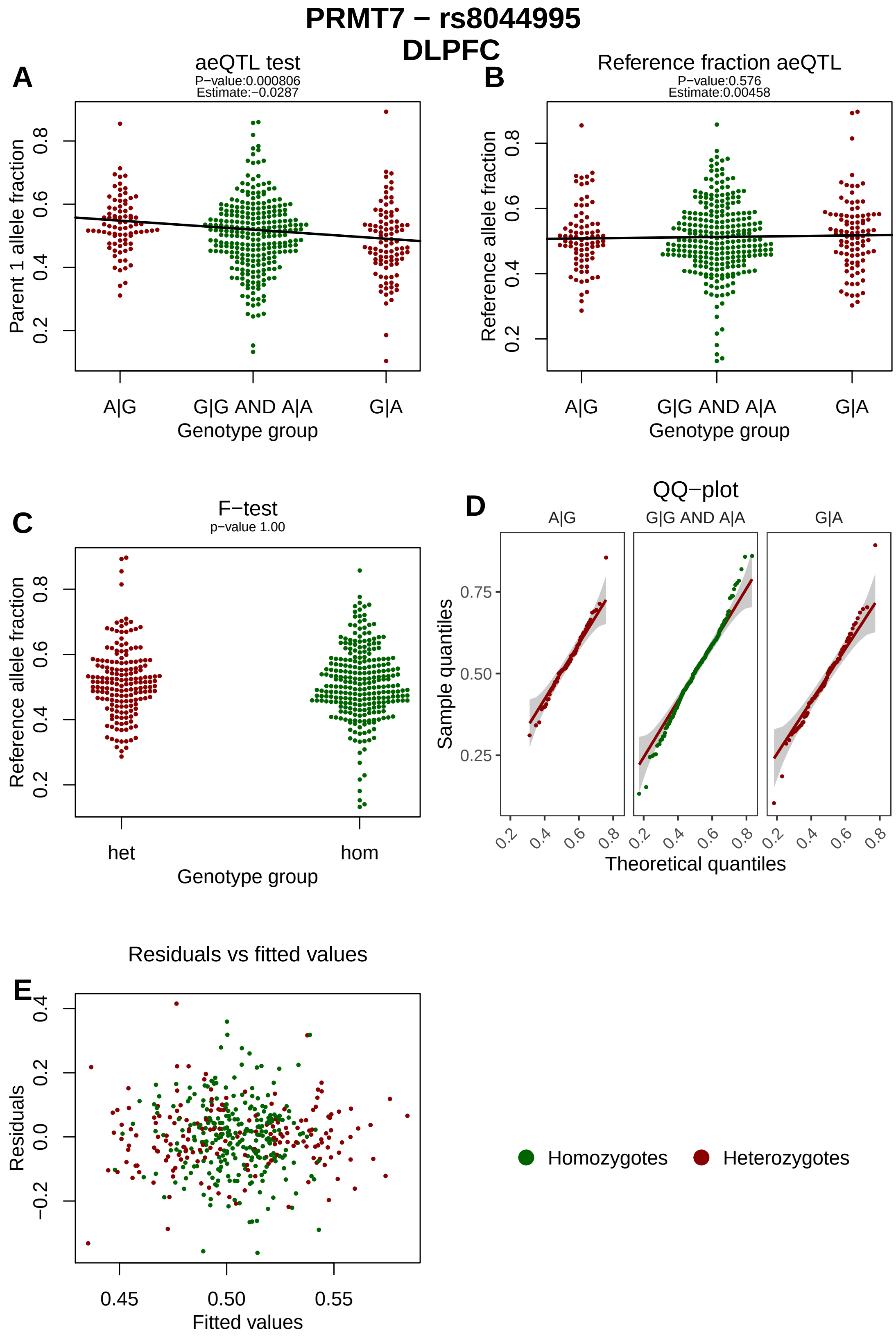

Figure S2−19: More details in the header of this document.

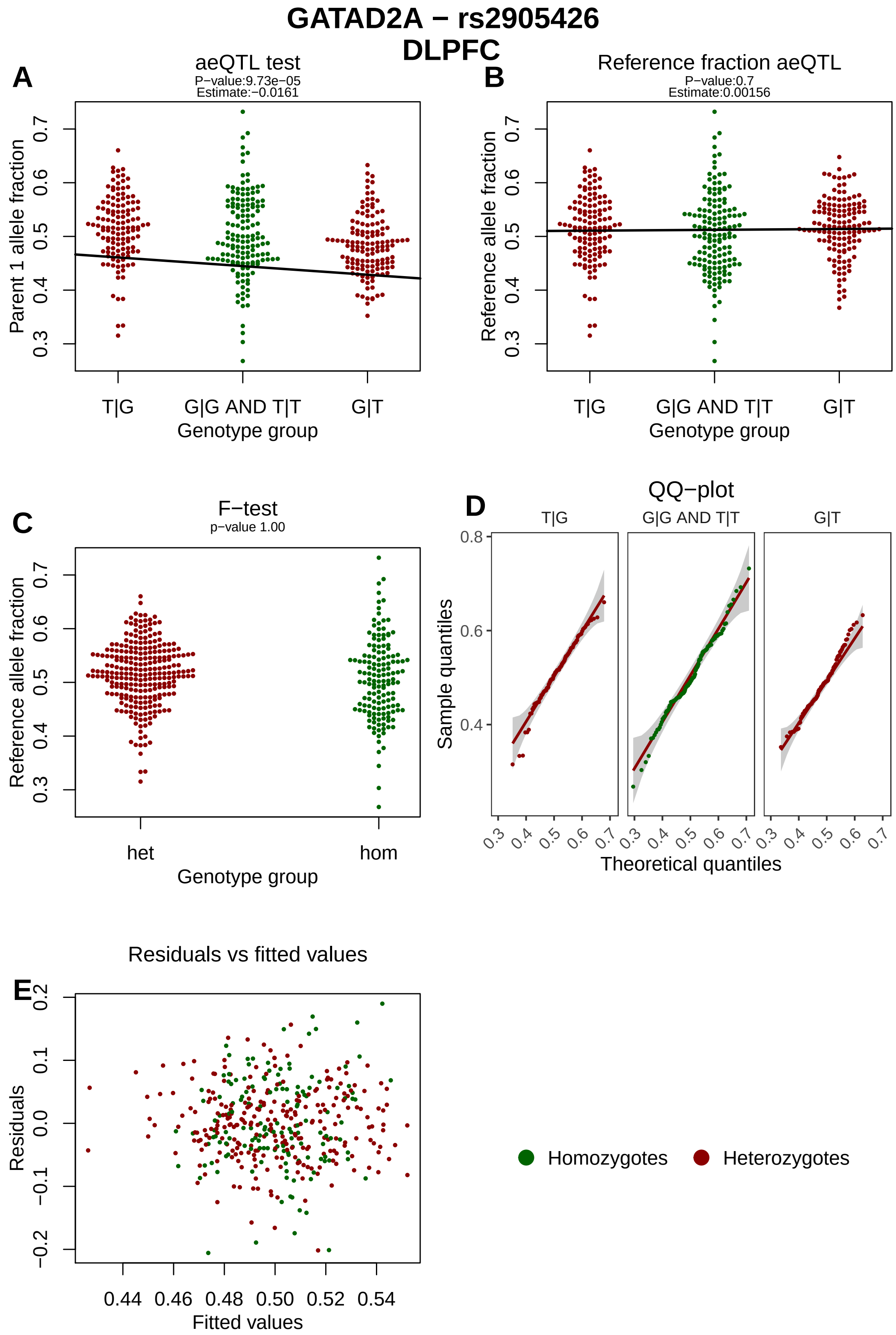

Figure S2−20: More details in the header of this document.

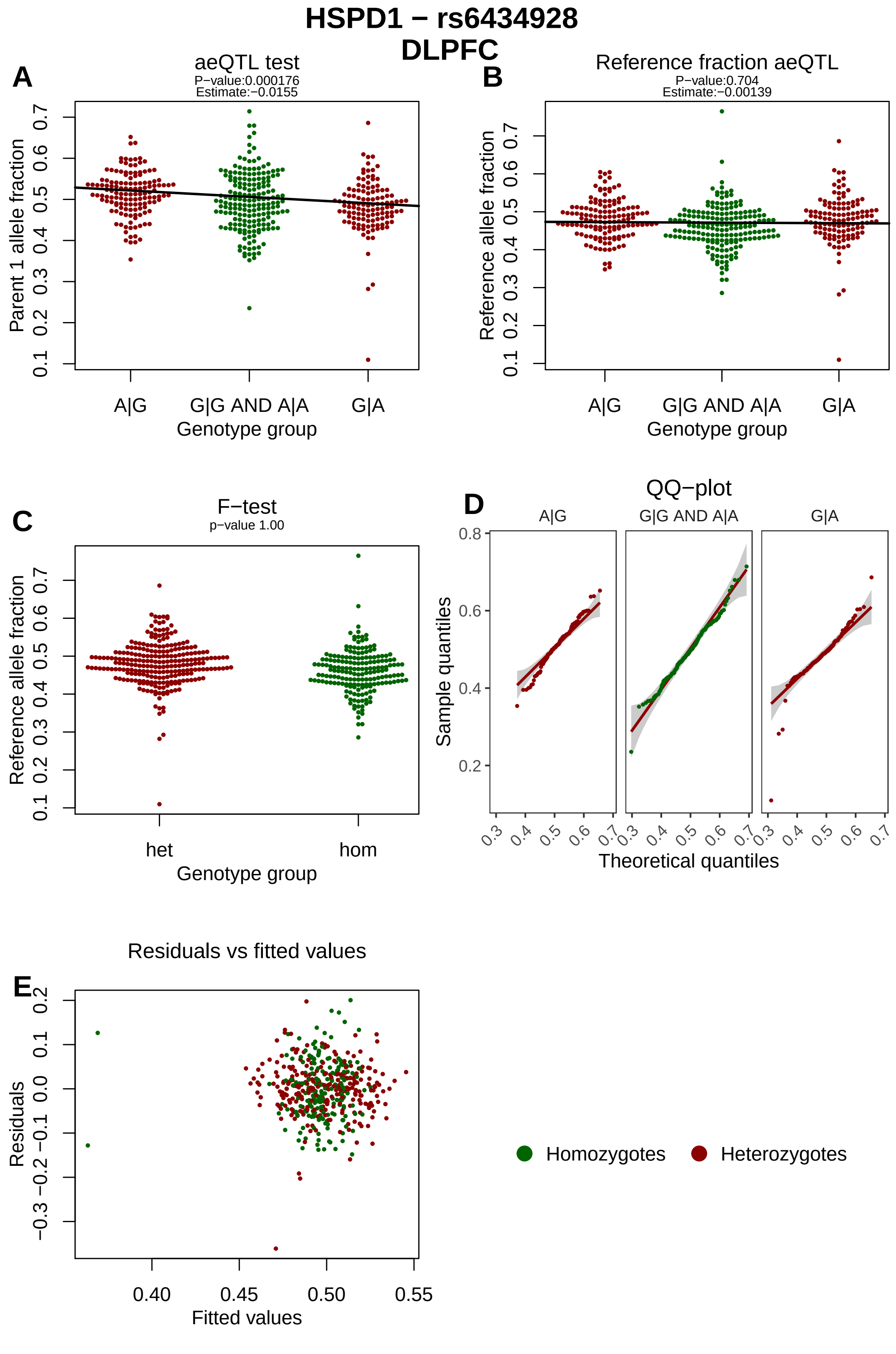

Figure S2−21: More details in the header of this document.

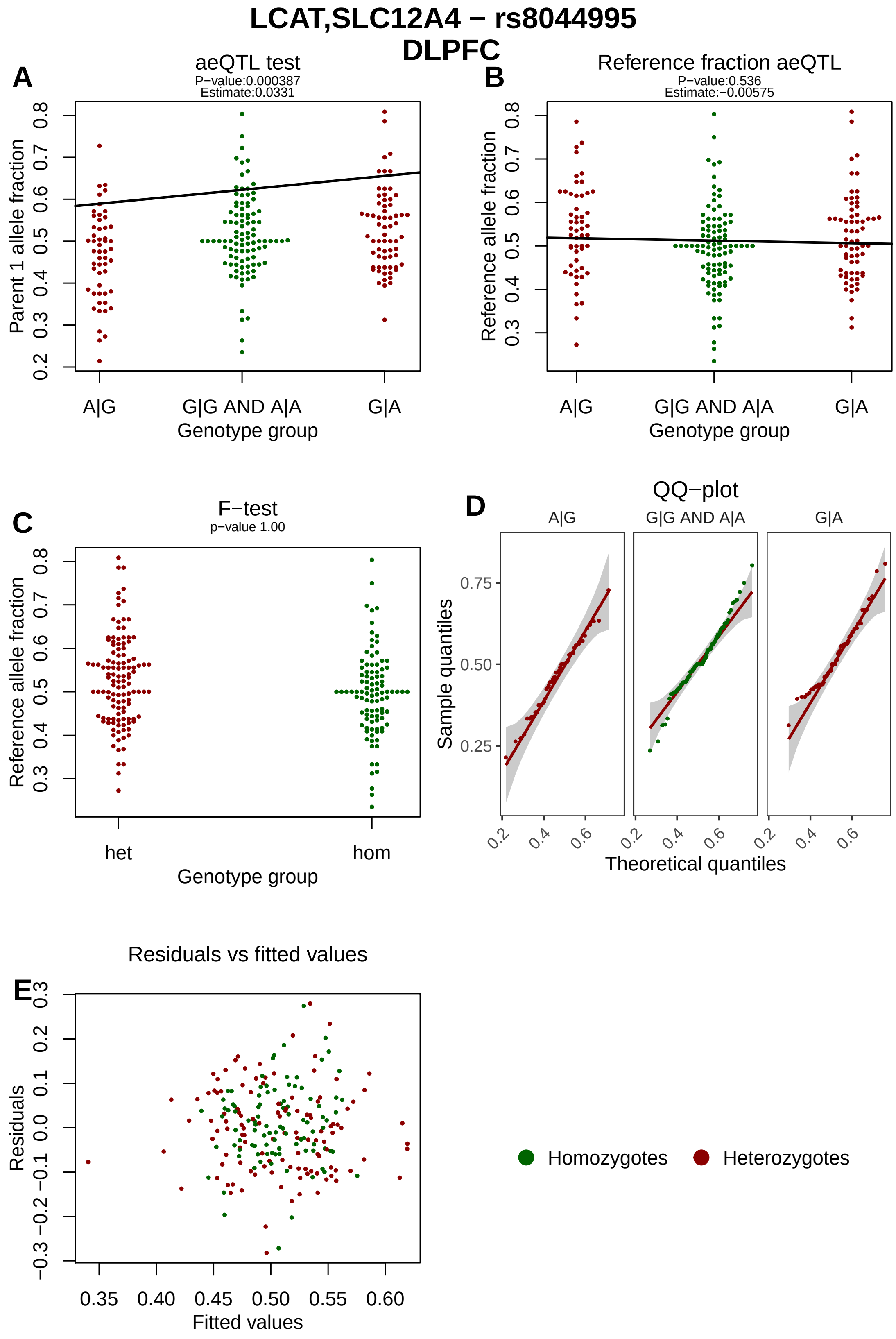

Figure S2−22: More details in the header of this document.

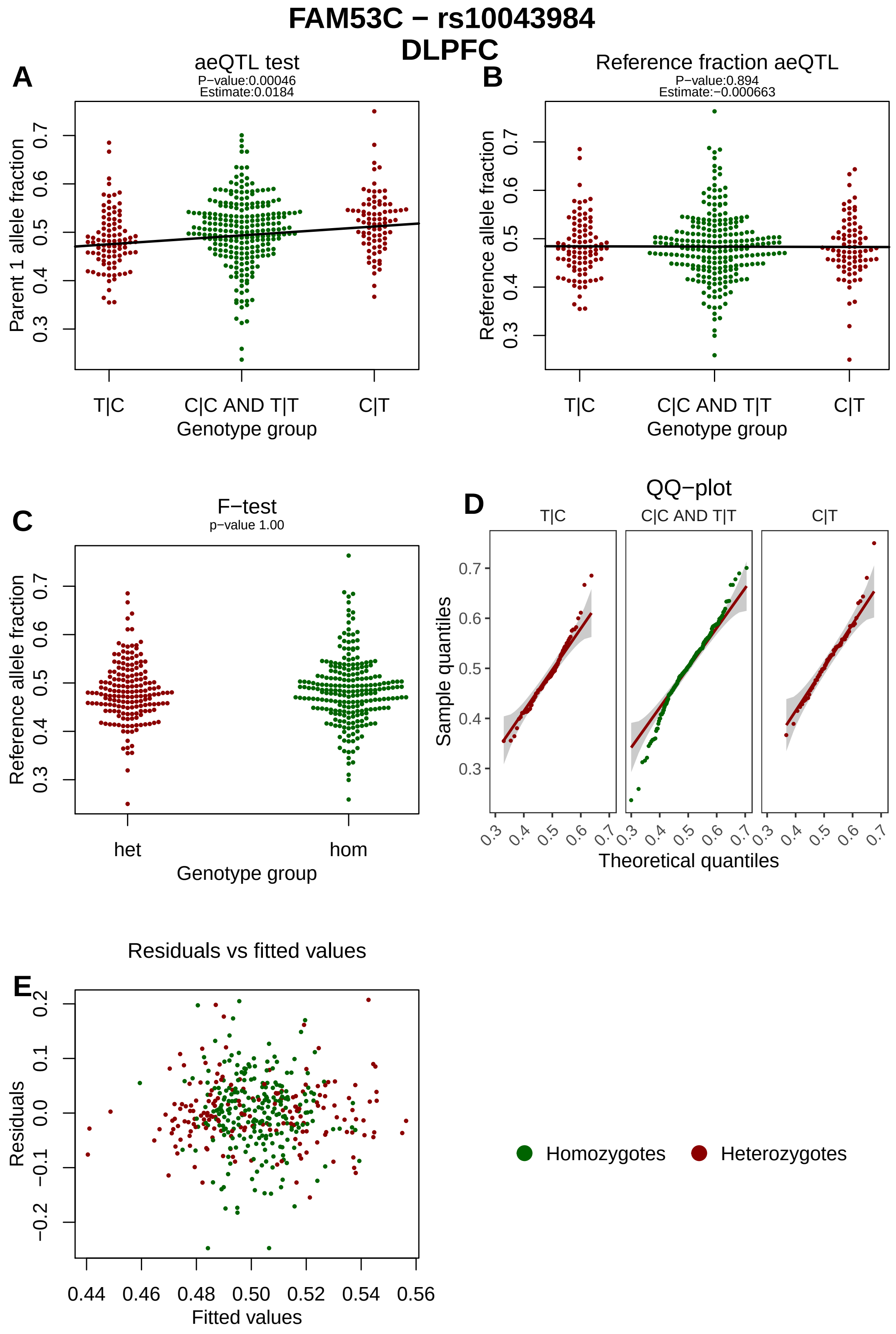

Figure S2−23: More details in the header of this document.

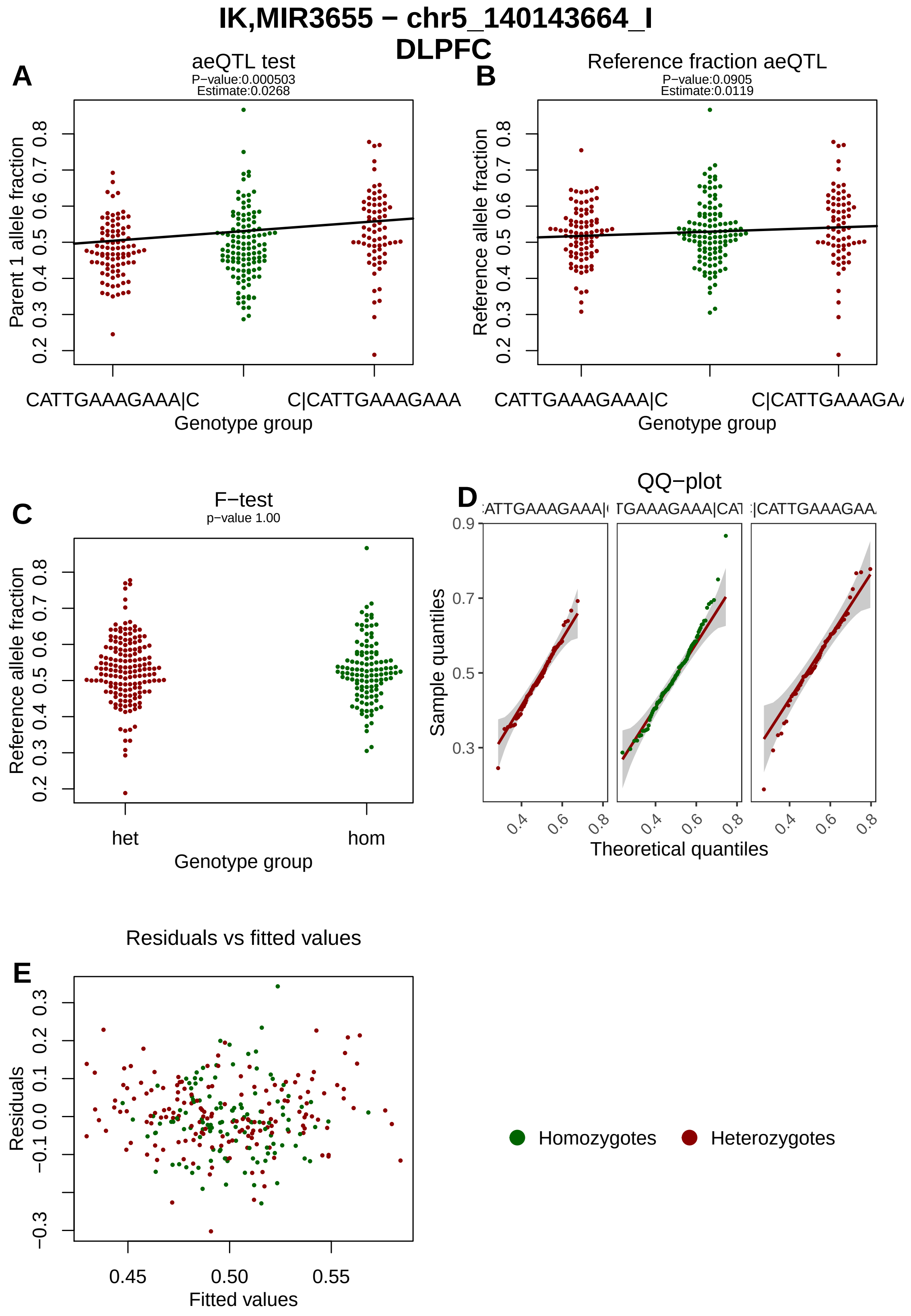

Figure S2−24: More details in the header of this document.

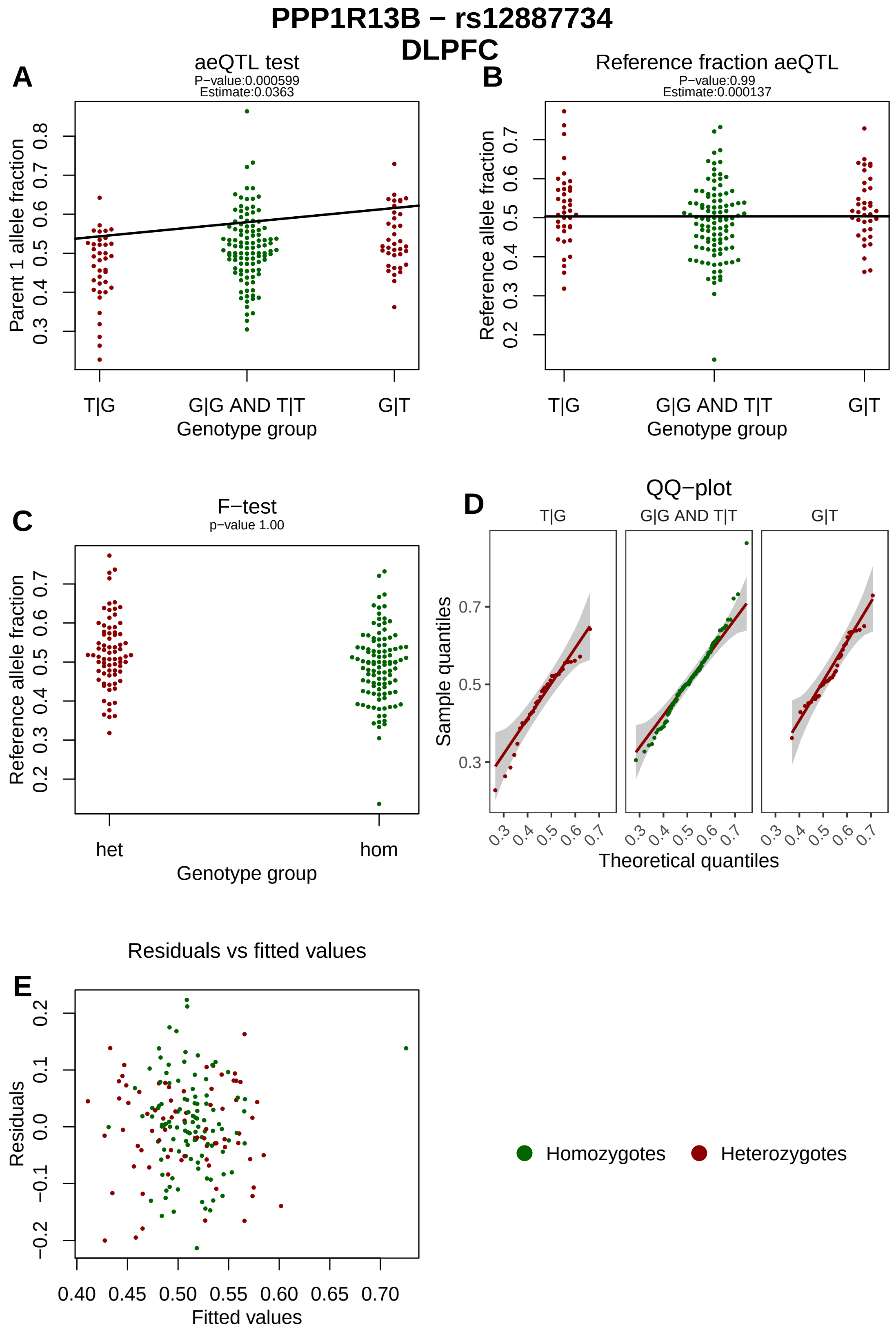

Figure S2−25: More details in the header of this document.

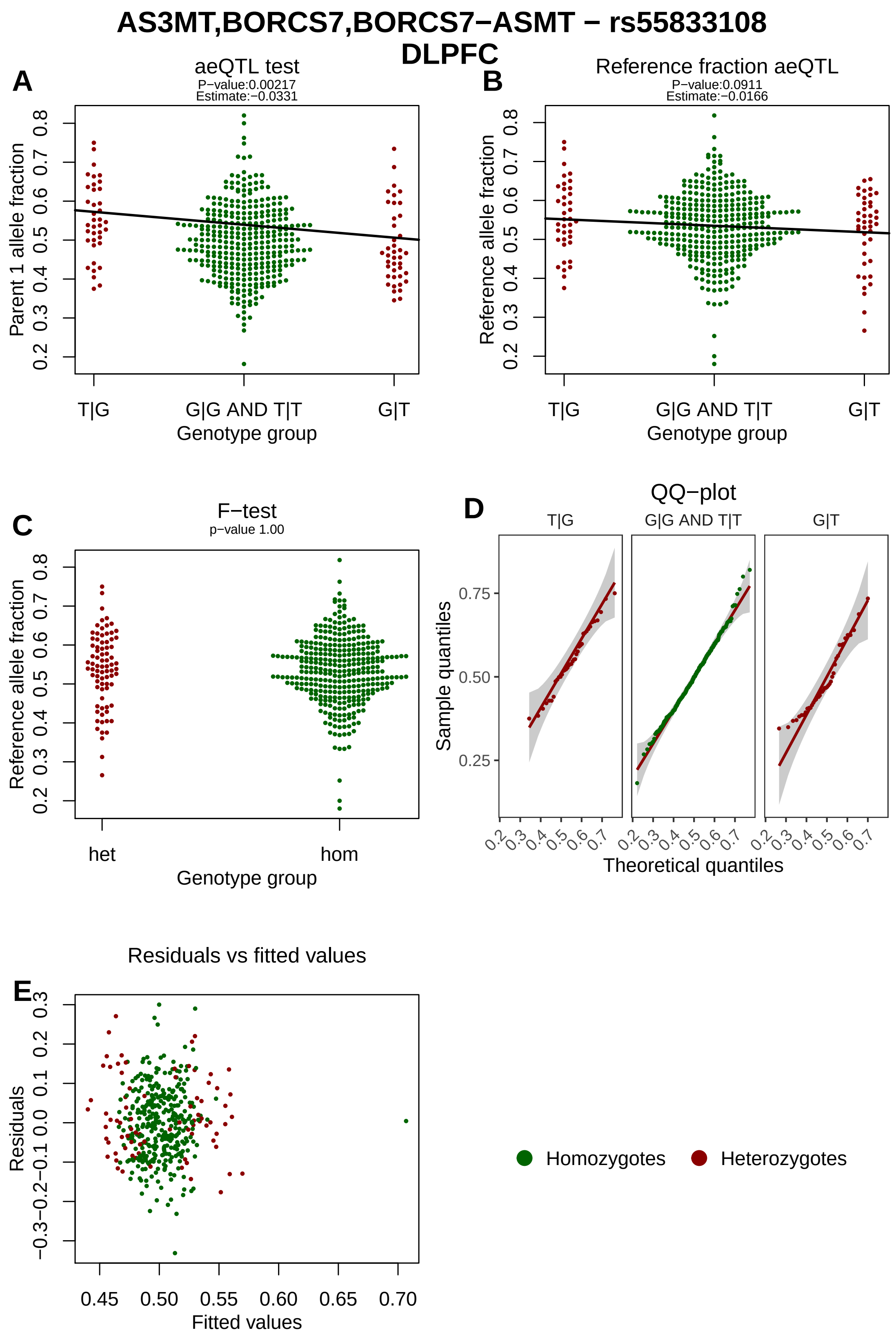

Figure S2−26: More details in the header of this document.

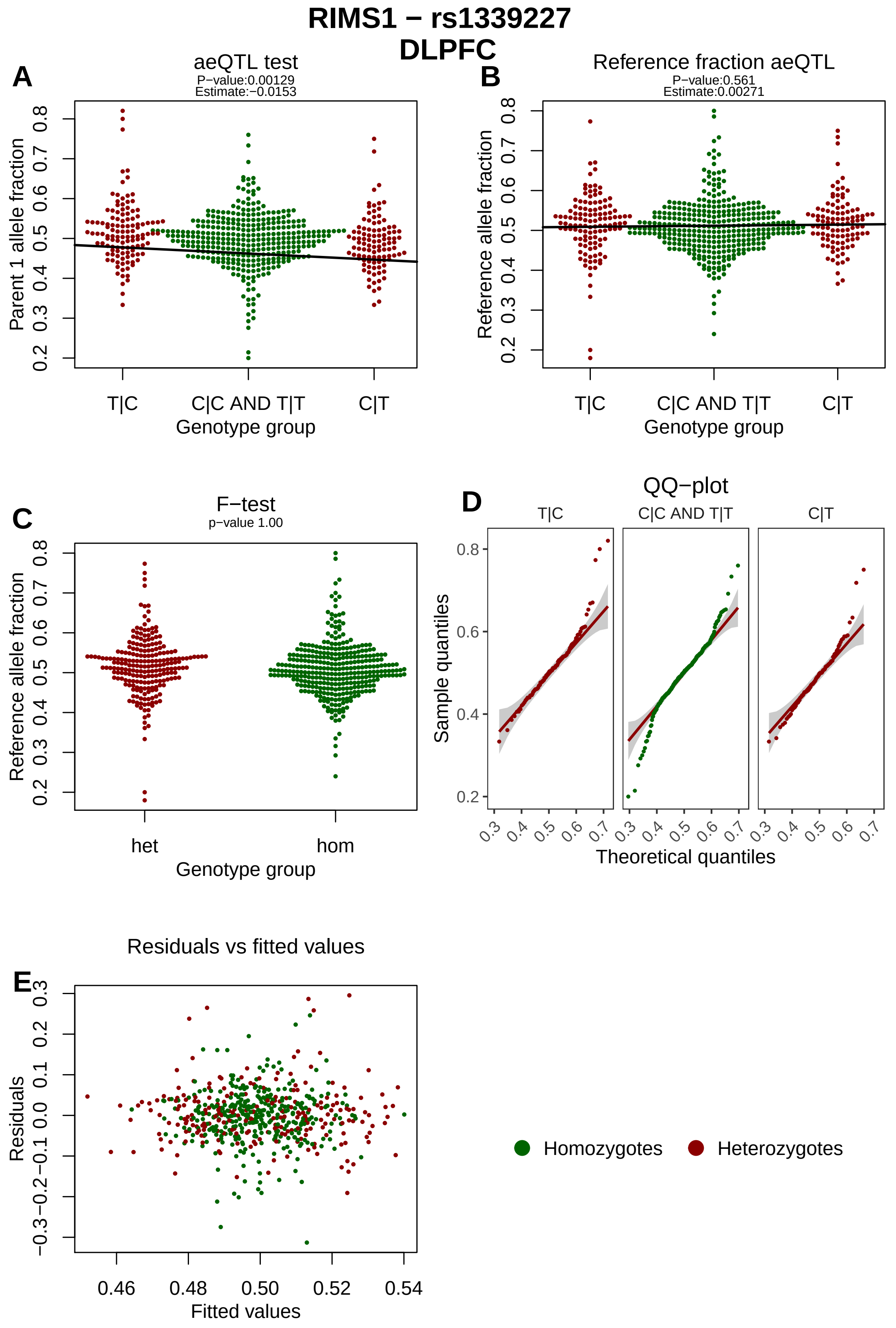

Figure S2−27: More details in the header of this document.

BUB1B,PAK6 − rs56205728
DLPFC

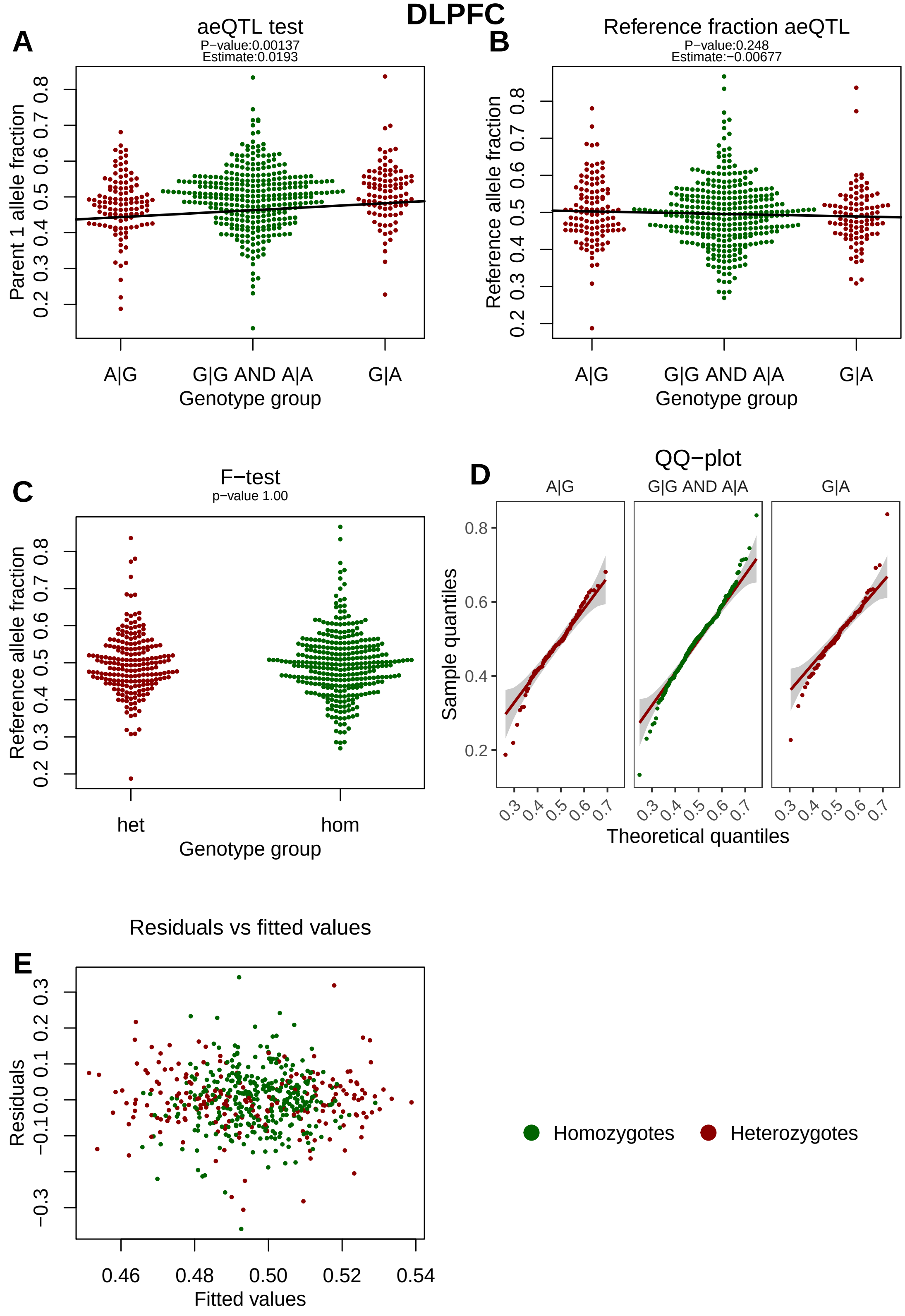

Figure S2−28: More details in the header of this document.

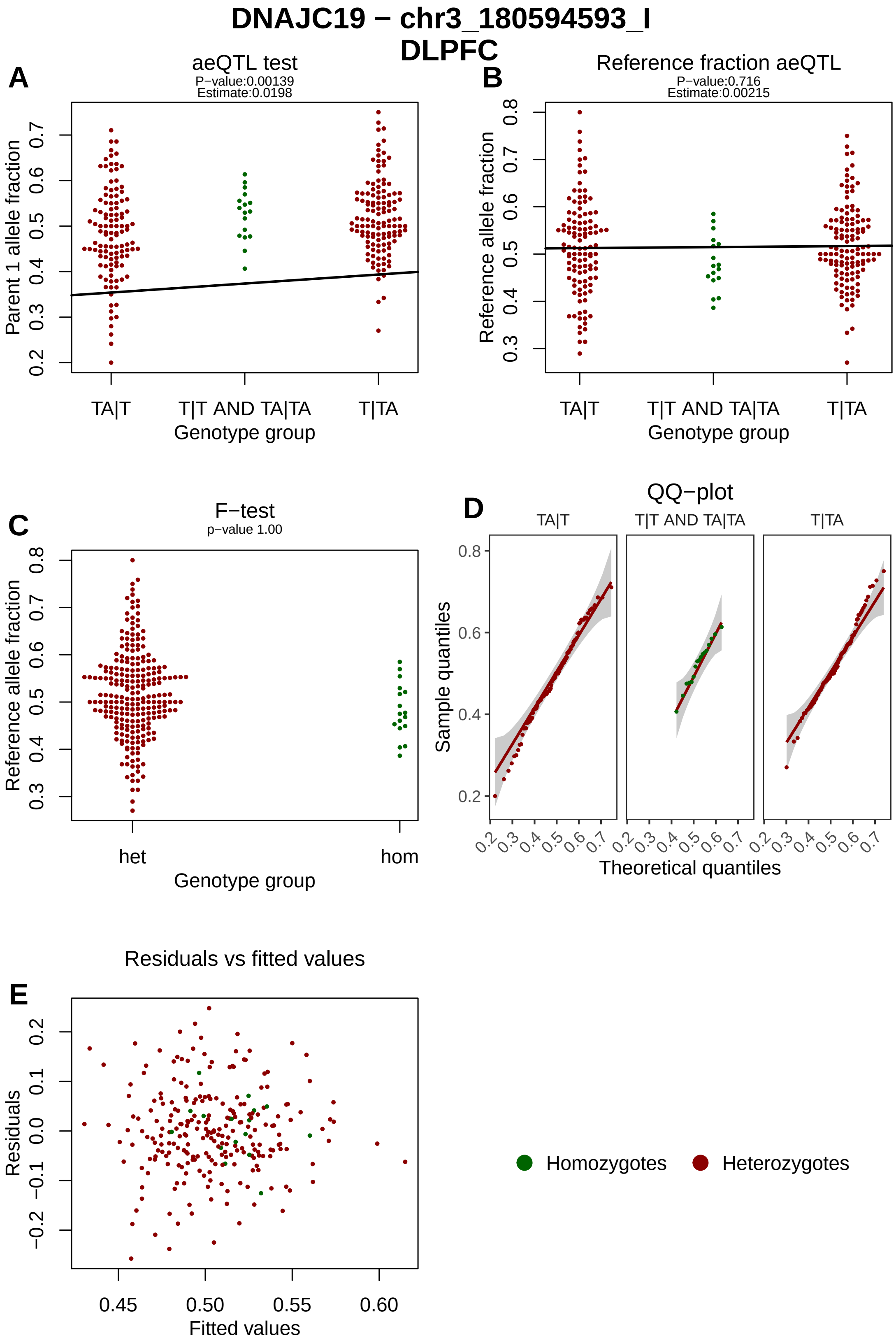

Figure S2−29: More details in the header of this document.

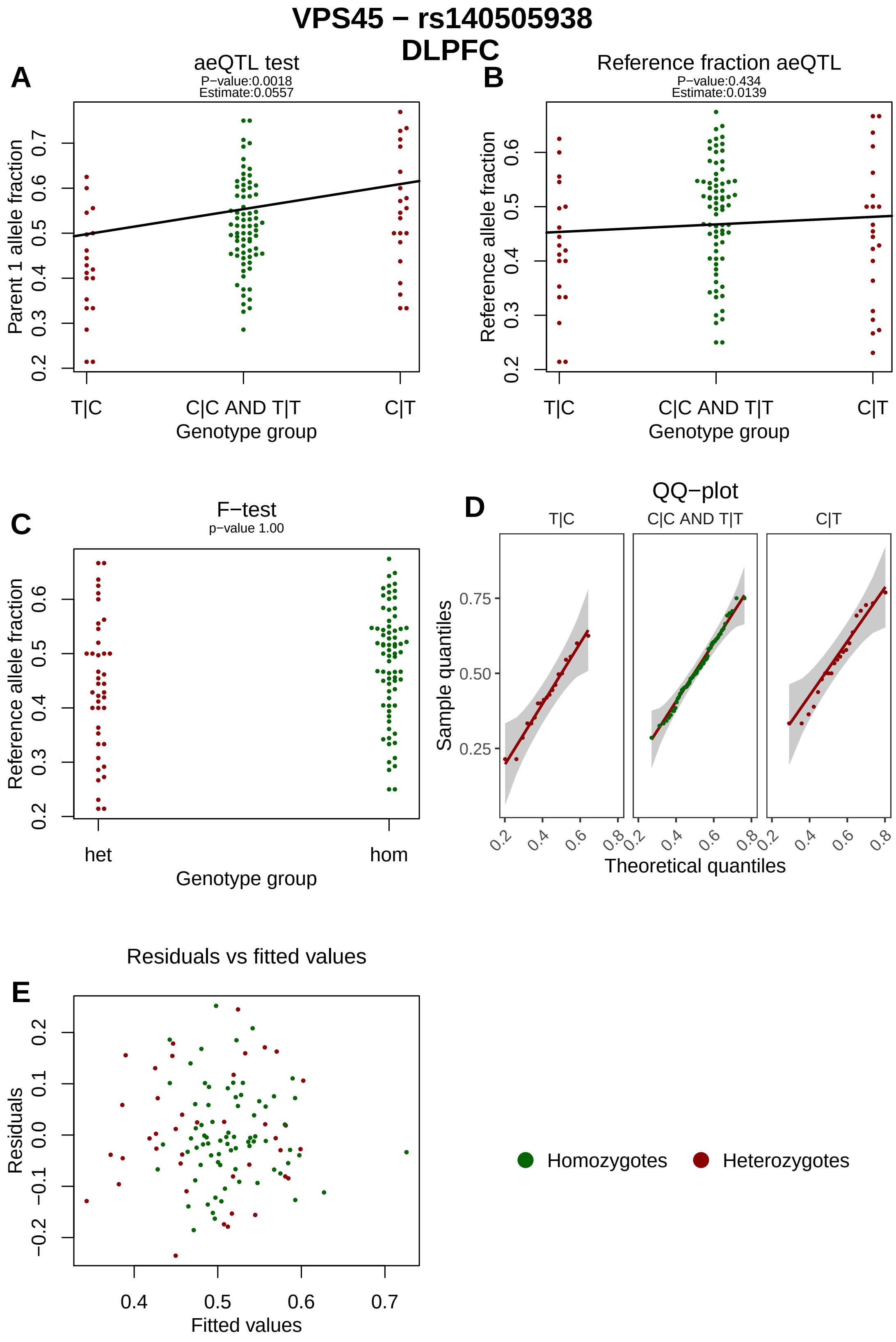

Figure S2−30: More details in the header of this document.

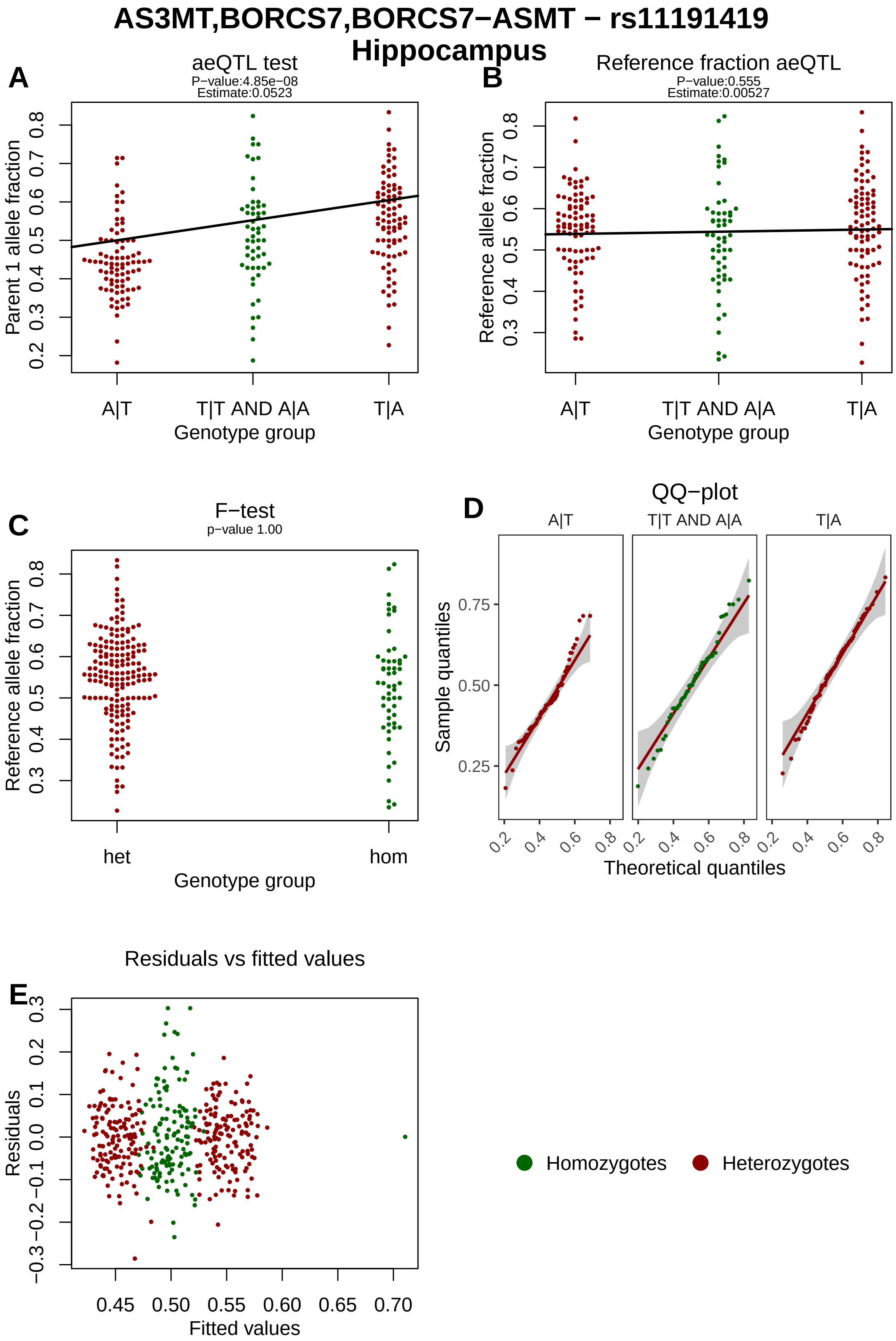

Figure S2−31: More details in the header of this document.

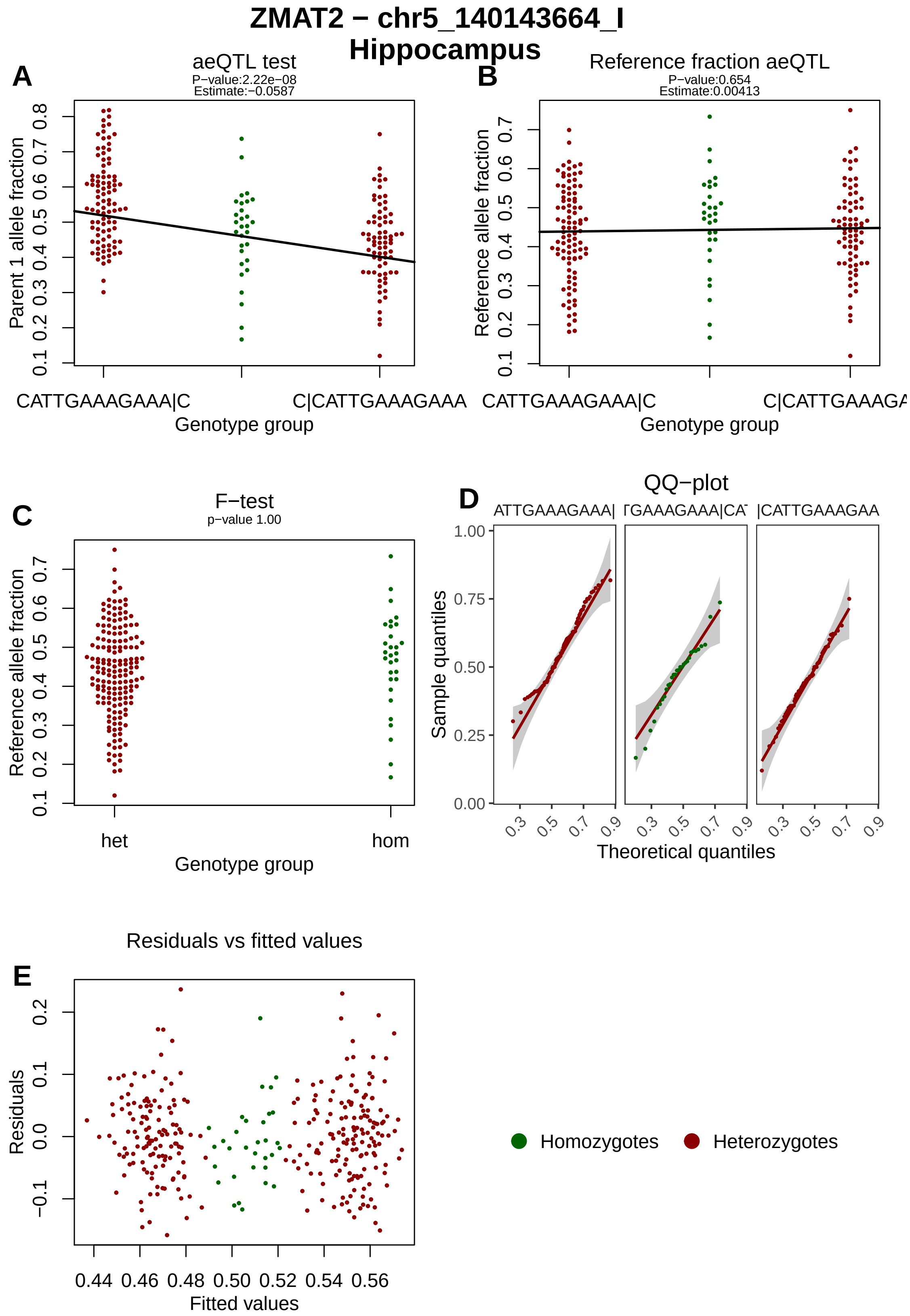

Figure S2−32: More details in the header of this document.

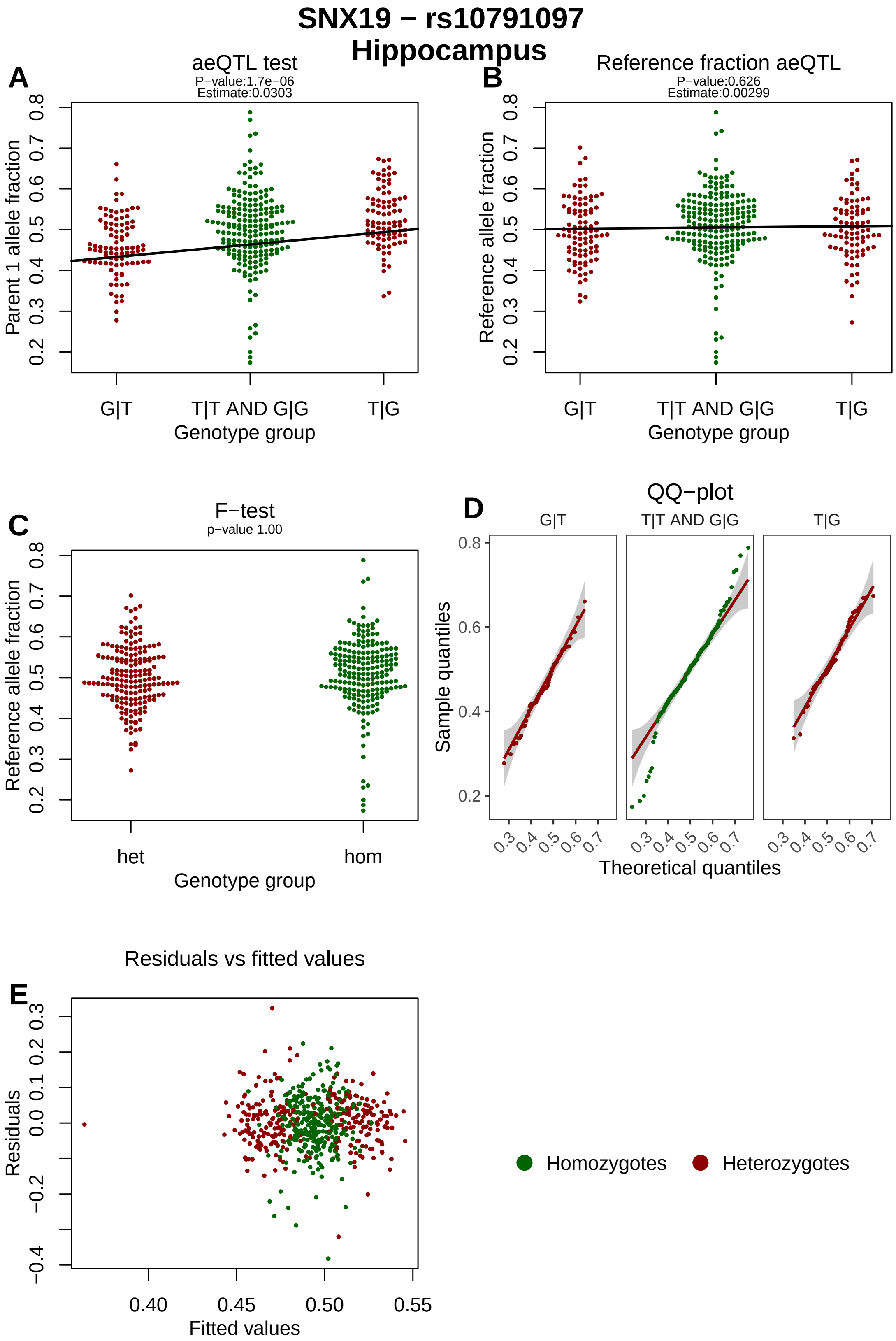

Figure S2−33: More details in the header of this document.

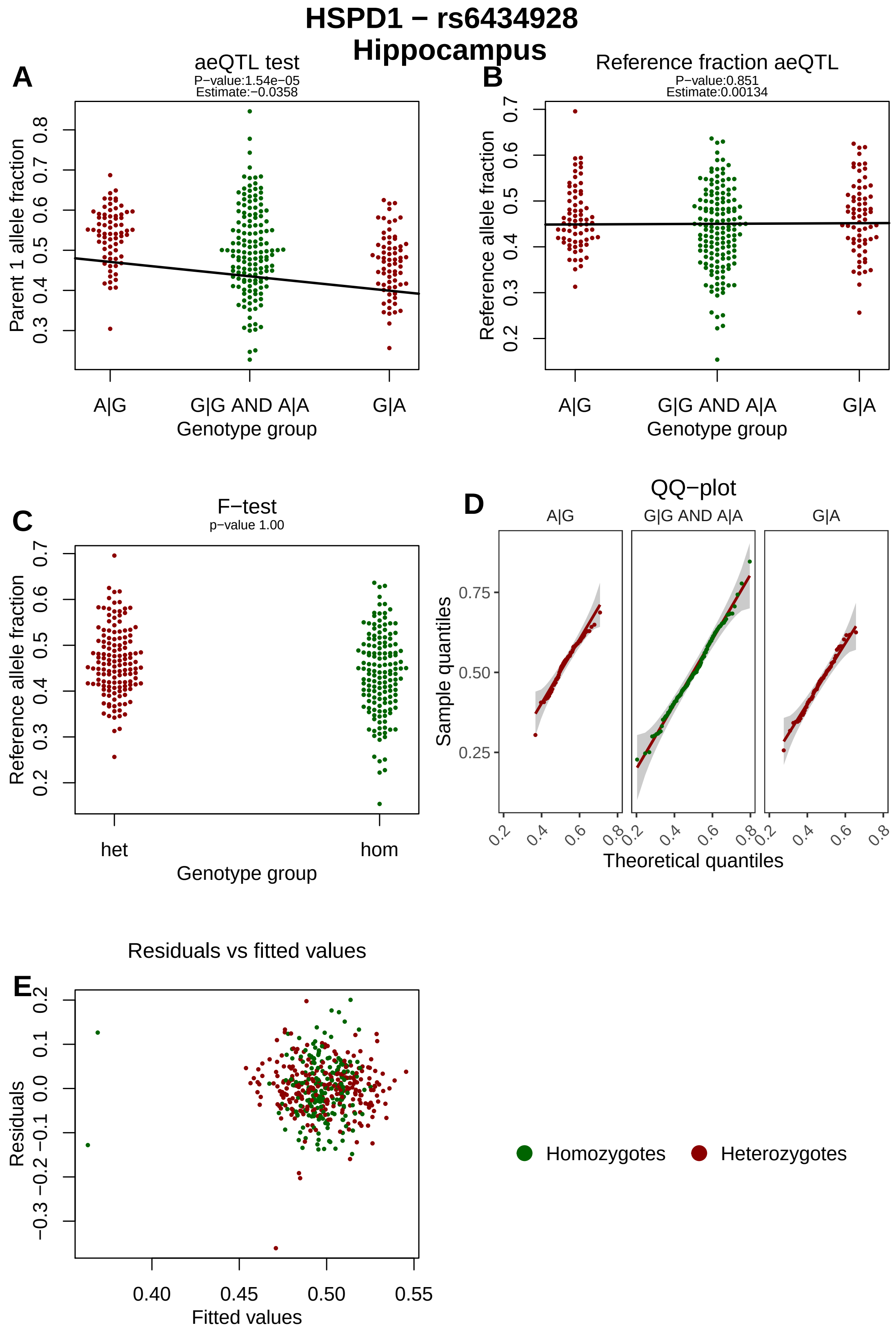

Figure S2−34: More details in the header of this document.
