# Supplementary material for: Comparison of quantitative trait loci methods: Total expression and allelic imbalance method in brain RNA-seq
Source: PLoS One. 2019 Jun 17;14(6):e0217765. doi: 10.1371/journal.pone.0217765 (PMC6576752; doi:10.1371/journal.pone.0217765)
Supplement: S3 File — The File contains the supplemental figures S3-1 to S3-64. (PDF) [file pone.0217765.s003.pdf]

Allelic Imbalance in SZ (Supplemental Material 3)

This is a document containing supplemental figures for the “Comparison of Quantitative Trait Loci Methods: Total Expression and Allelic Imbalance Method in Brain RNA-seq” manuscript. All riskSNPs present in the main manuscripts table 1 is presented on a separate page in a similar location plot as figure 3 in the main manuscript.

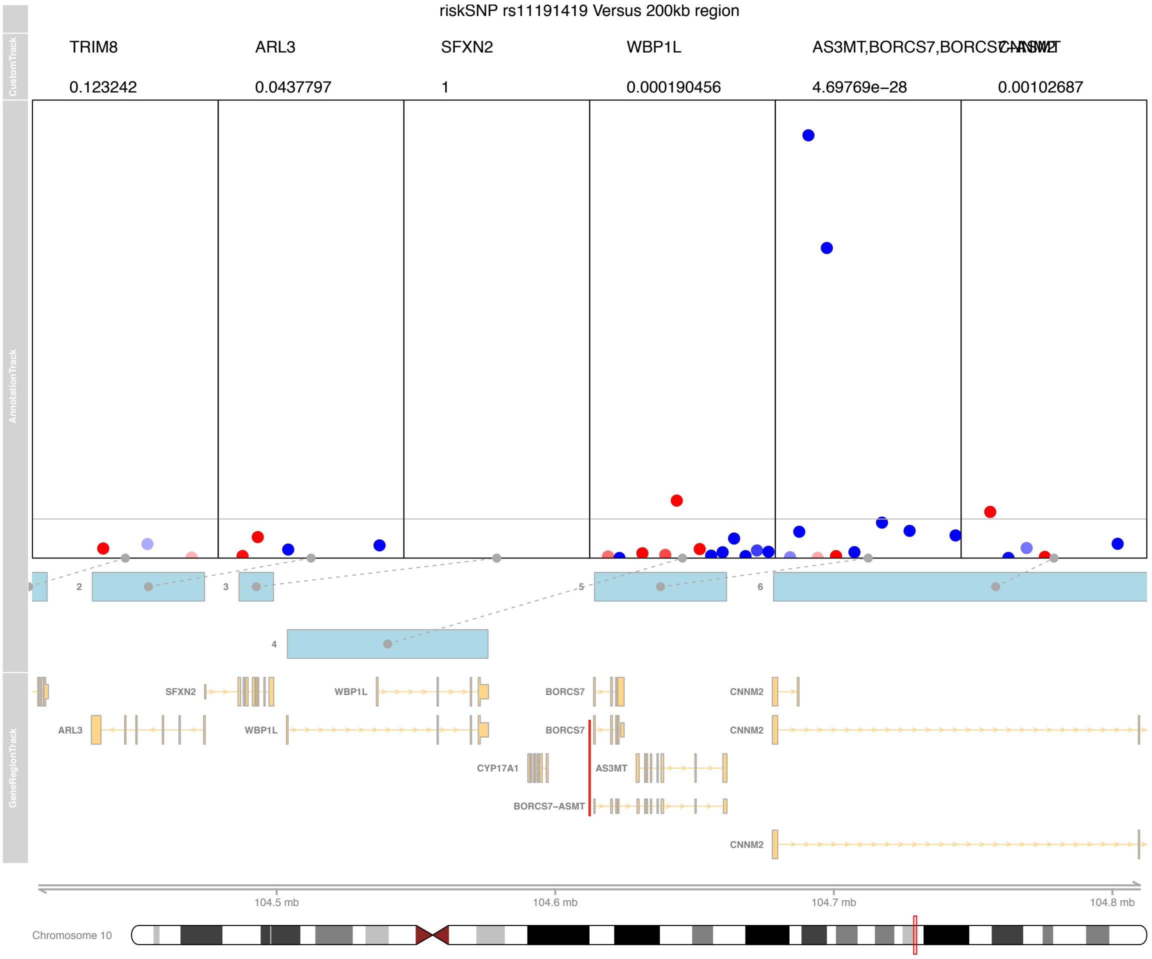

Figure S3-1. riskSNP-centric plot for DLPFC similar to figure 3 in the main manuscript.

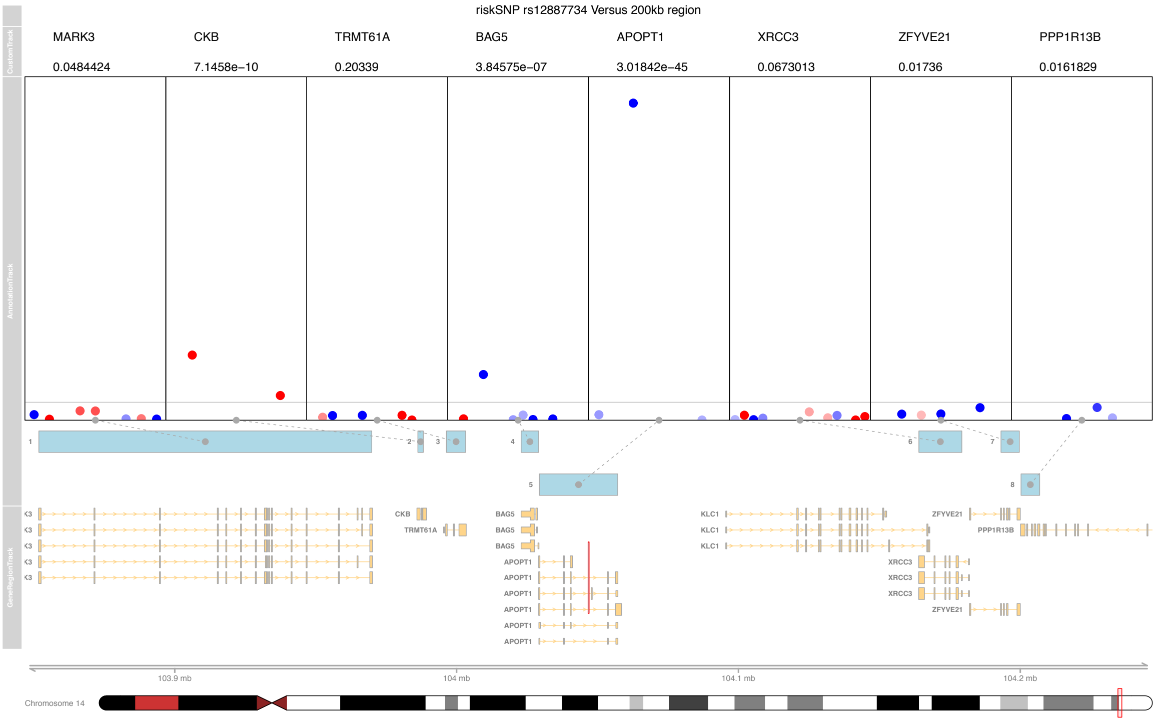

Figure S3-2. riskSNP-centric plot for DLPFC similar to figure 3 in the main manuscript.

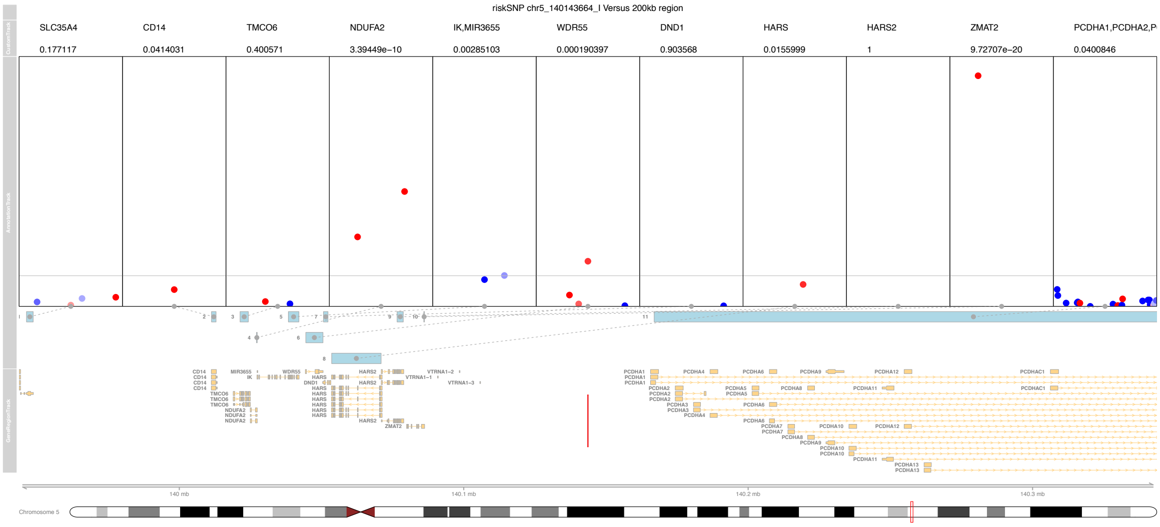

Figure S3-3. riskSNP-centric plot for DLPFC similar to figure 3 in the main manuscript.

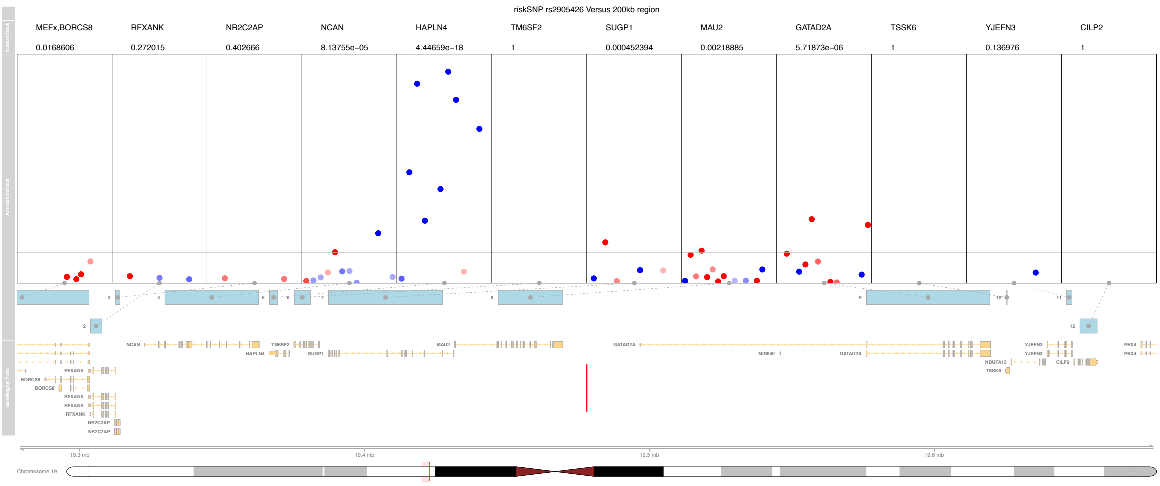

Figure S3-4. riskSNP-centric plot for DLPFC similar to figure 3 in the main manuscript.

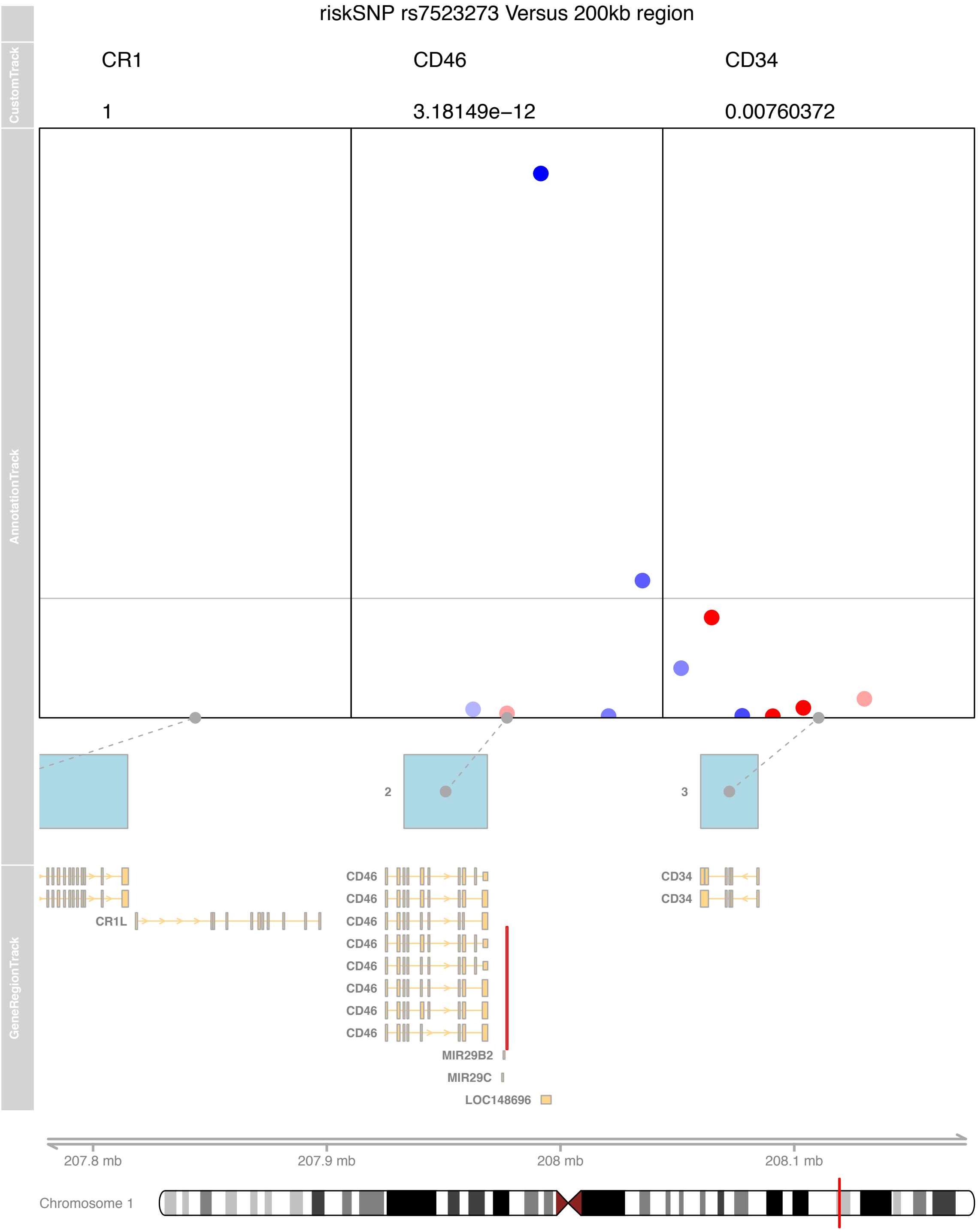

Figure S3-5. riskSNP-centric plot for DLPFC similar to figure 3 in the main manuscript.

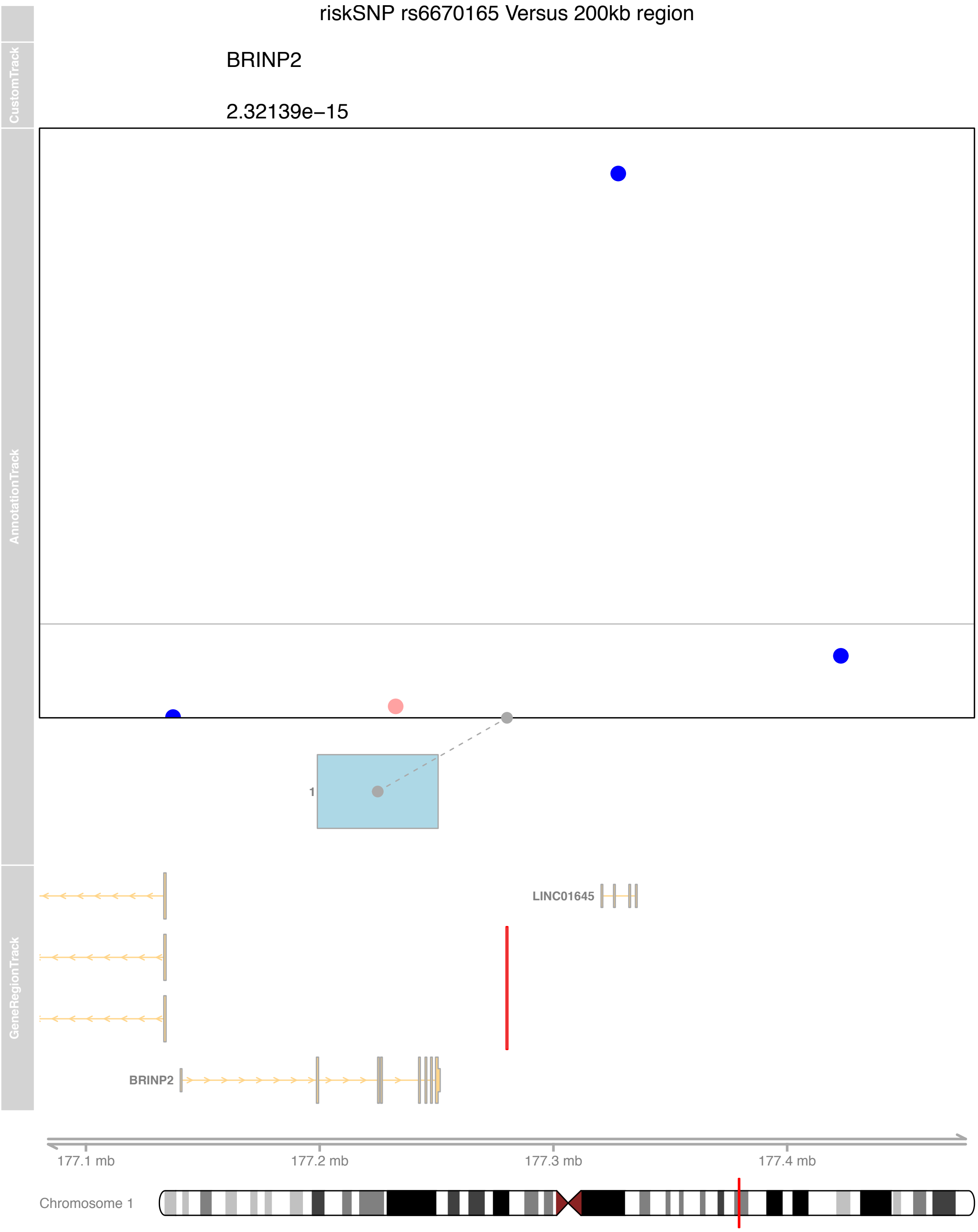

Figure S3-6. riskSNP-centric plot for DLPFC similar to figure 3 in the main manuscript.

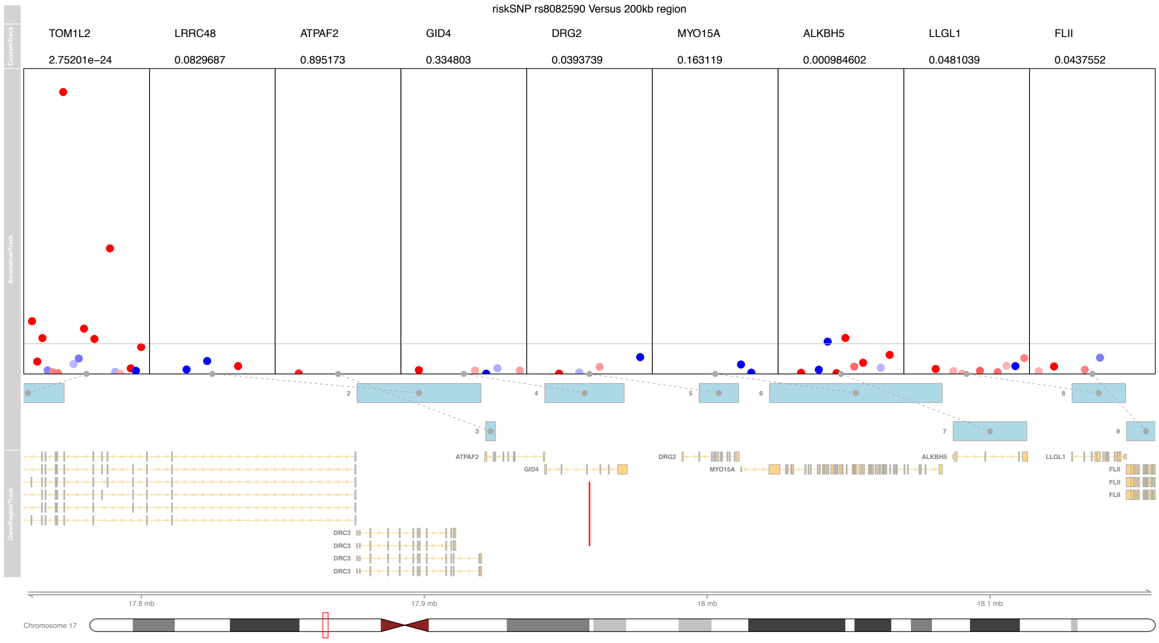

Figure S3-7. riskSNP-centric plot for DLPFC similar to figure 3 in the main manuscript.

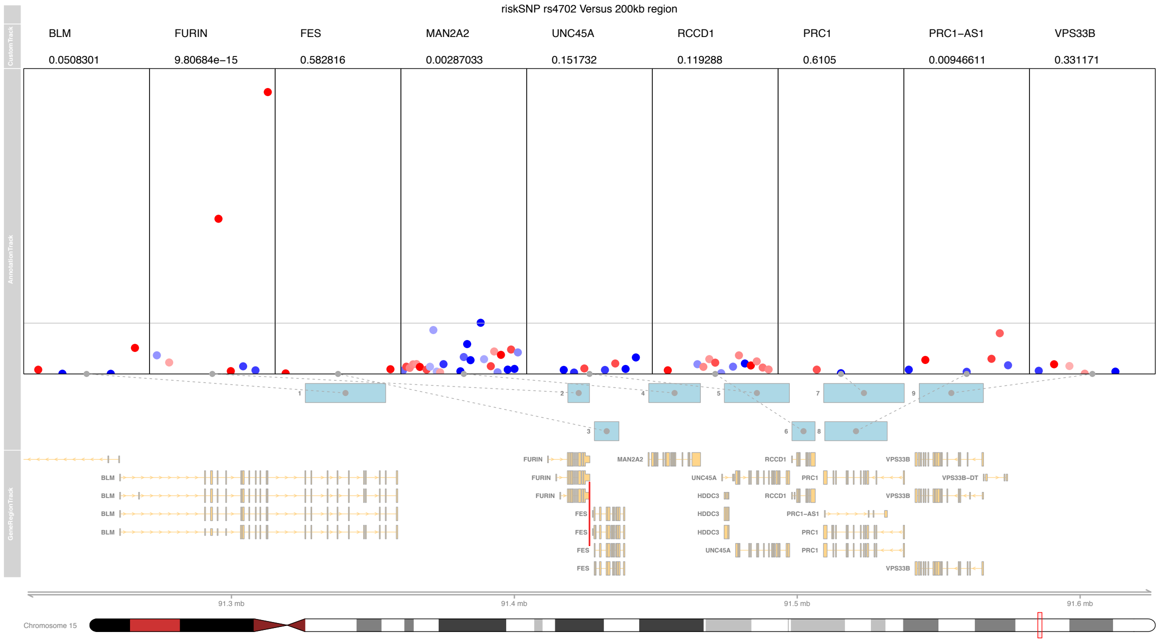

Figure S3-8. riskSNP-centric plot for DLPFC similar to figure 3 in the main manuscript.

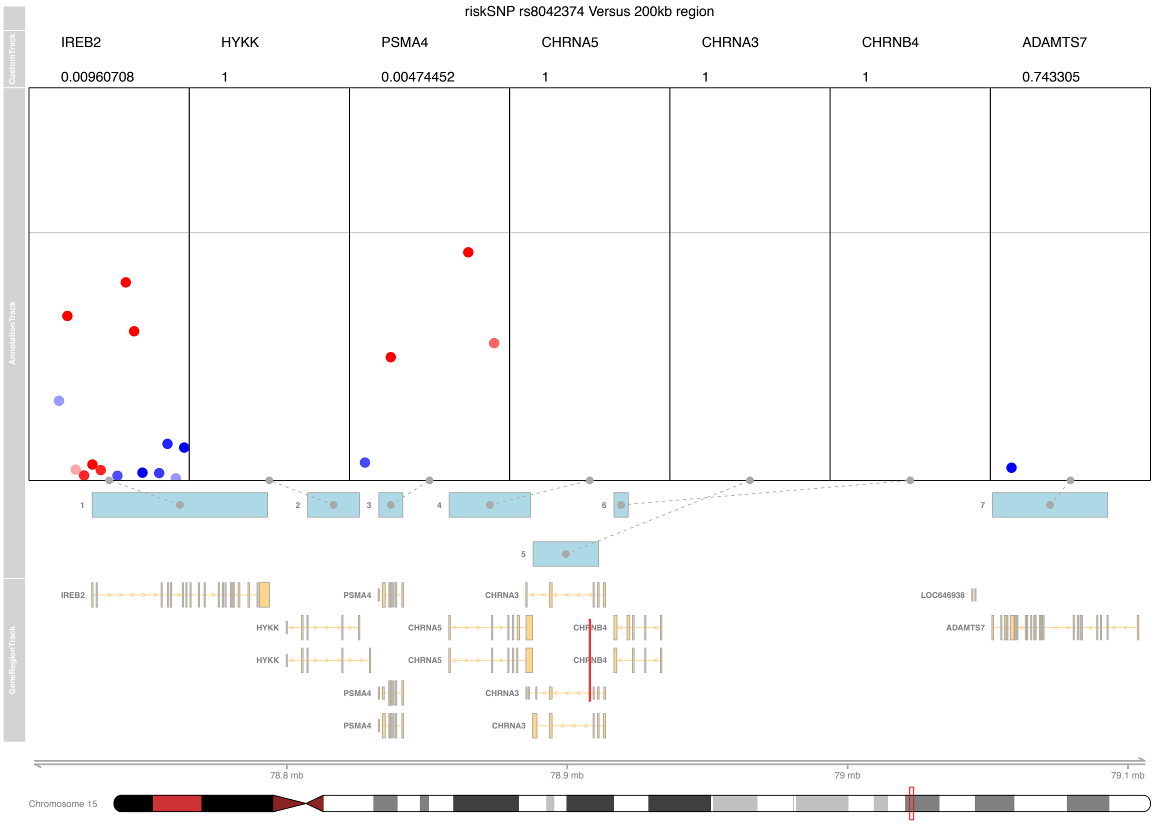

Figure S3-9. riskSNP-centric plot for DLPFC similar to figure 3 in the main manuscript.

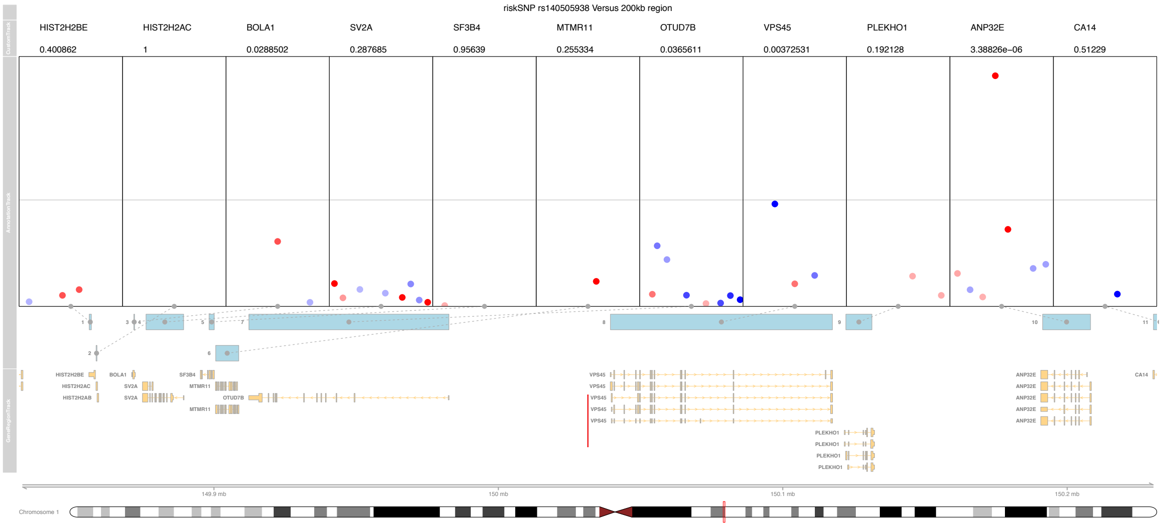

Figure S3-10. riskSNP-centric plot for DLPFC similar to figure 3 in the main manuscript.

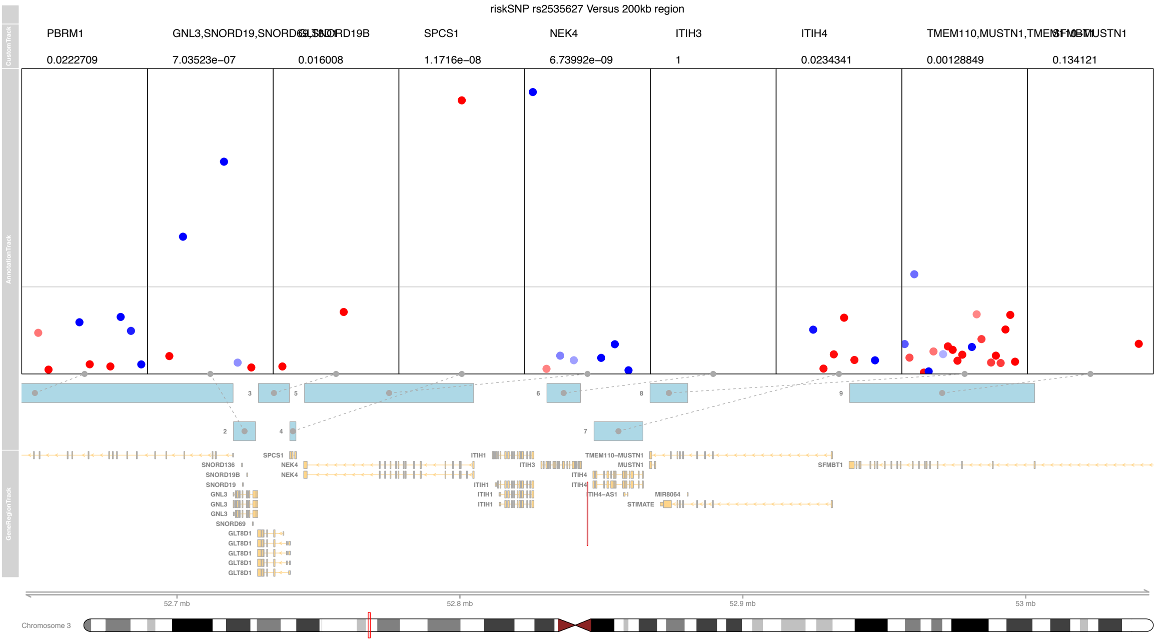

Figure S3-11. riskSNP-centric plot for DLPFC similar to figure 3 in the main manuscript.

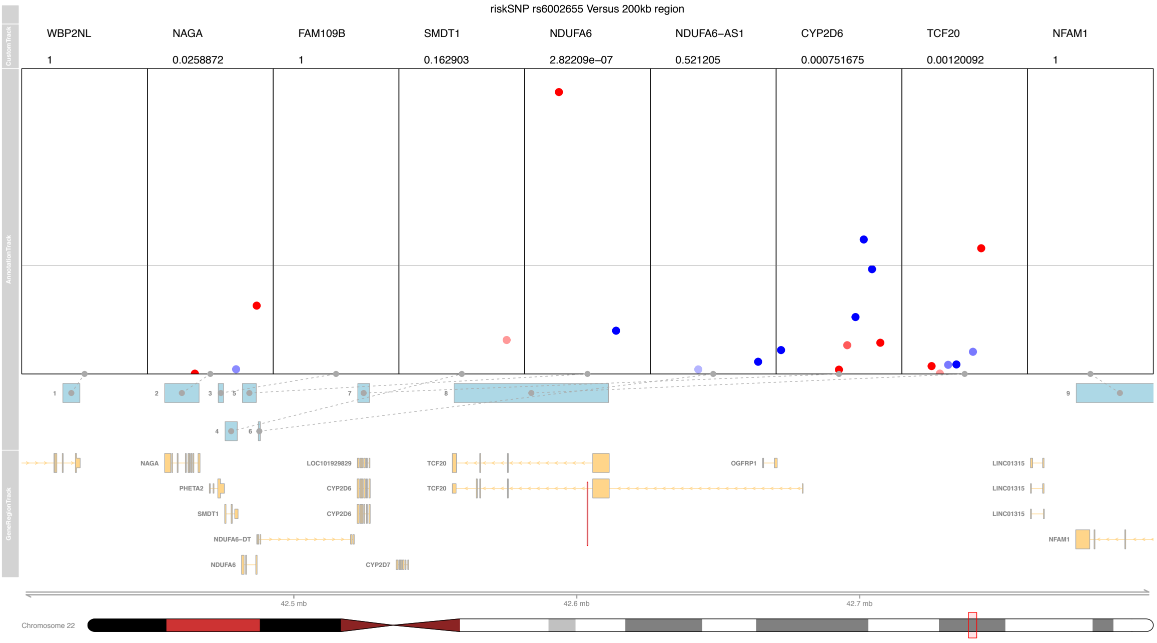

Figure S3-12. riskSNP-centric plot for DLPFC similar to figure 3 in the main manuscript.

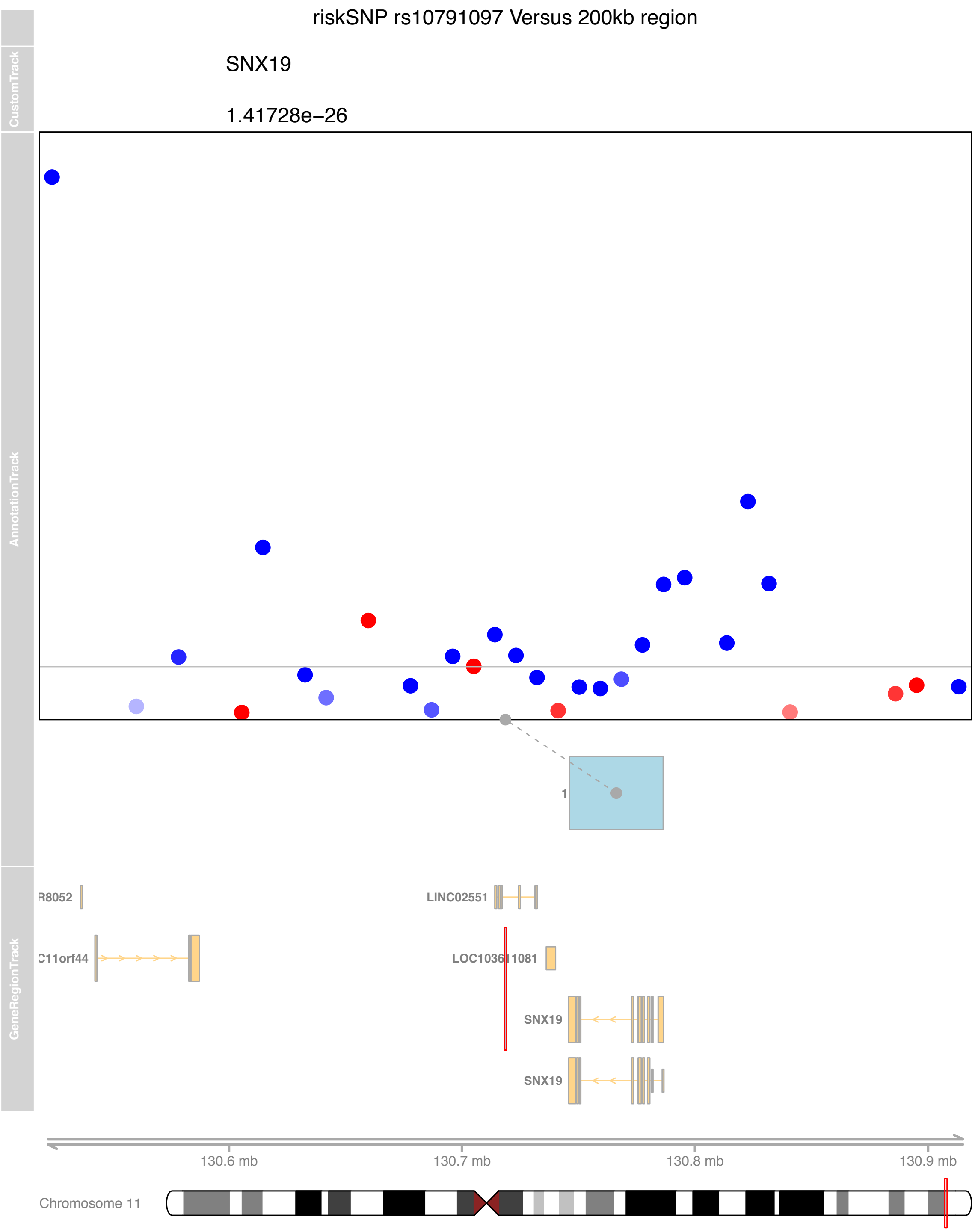

Figure S3-13. riskSNP-centric plot for DLPFC similar to figure 3 in the main manuscript.

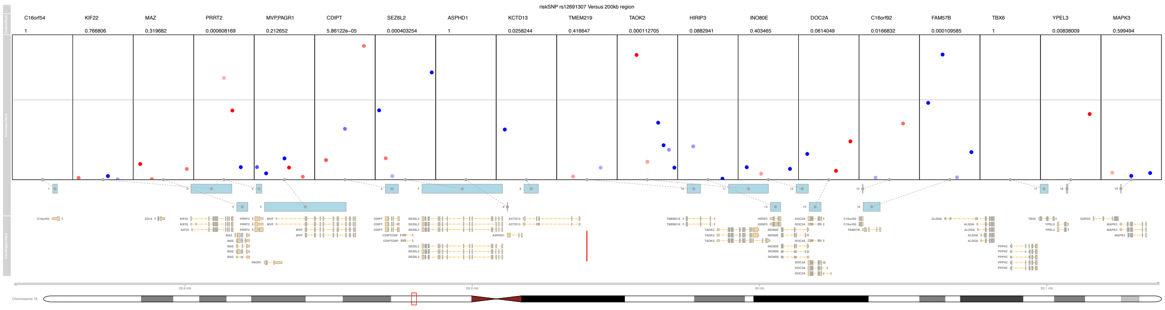

Figure S3-14. riskSNP-centric plot for DLPFC similar to figure 3 in the main manuscript.

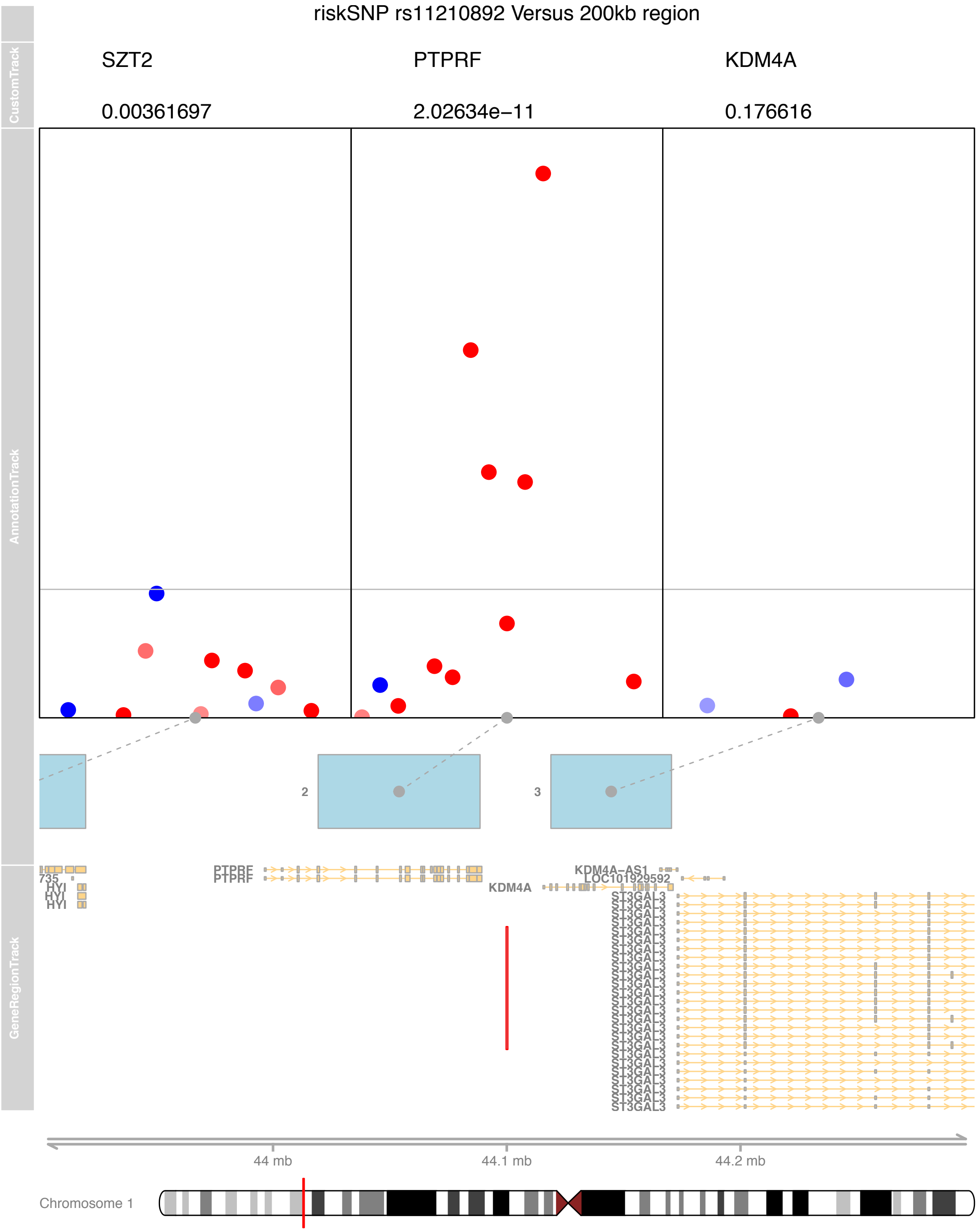

Figure S3-15. riskSNP-centric plot for DLPFC similar to figure 3 in the main manuscript.

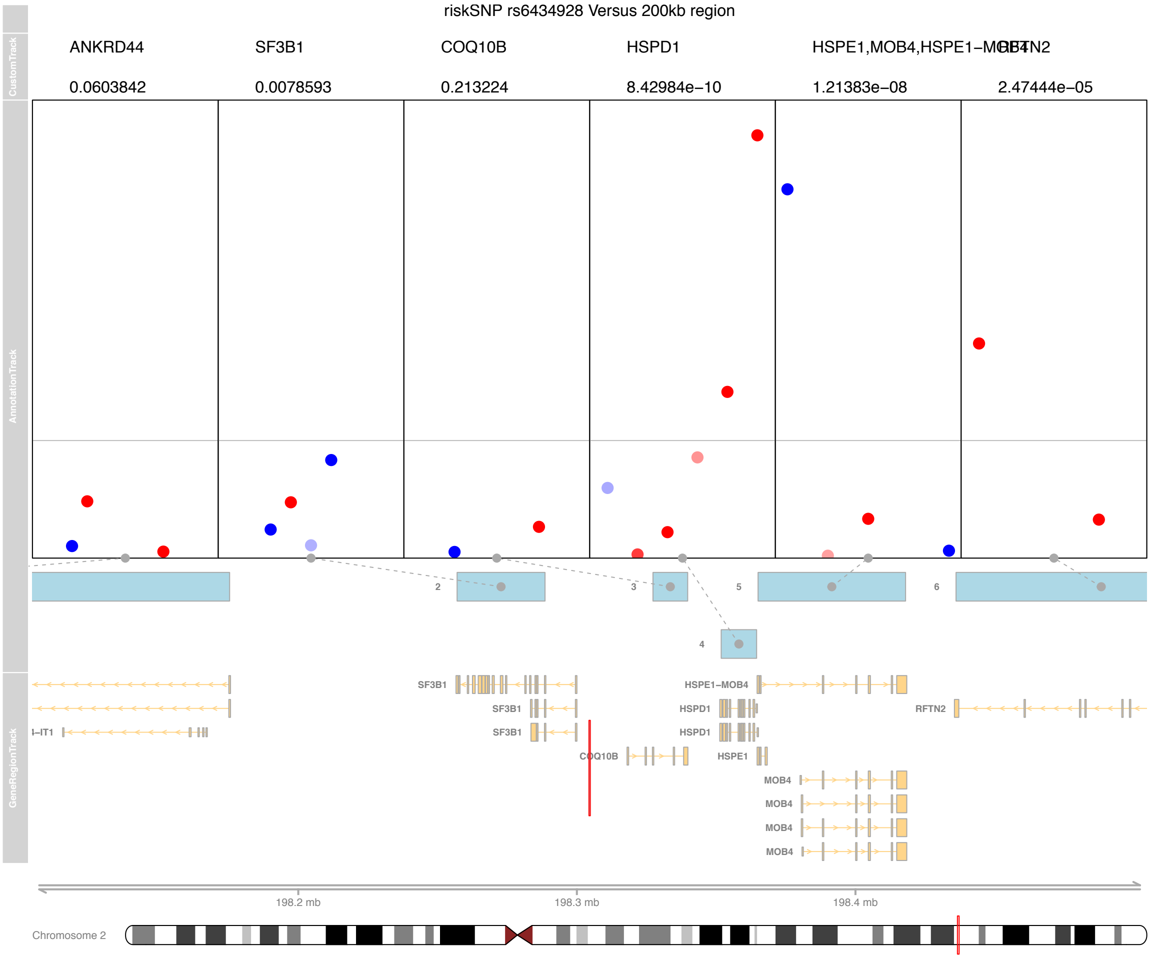

Figure S3-16. riskSNP-centric plot for DLPFC similar to figure 3 in the main manuscript.

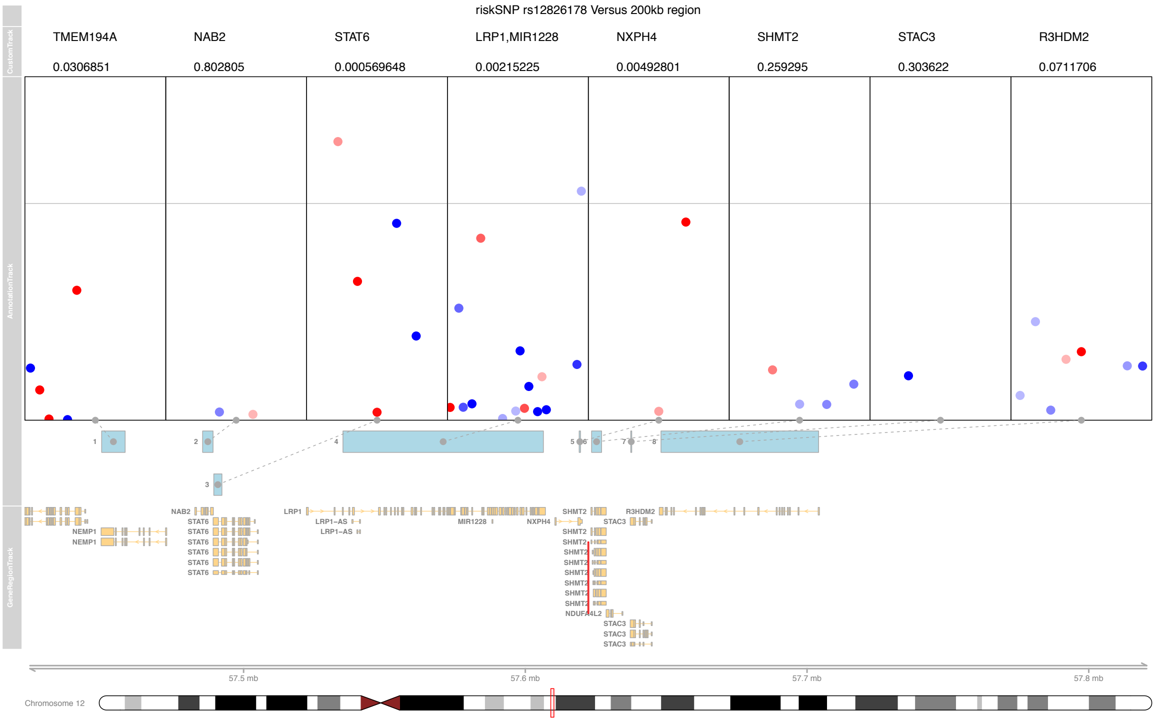

Figure S3-17. riskSNP-centric plot for DLPFC similar to figure 3 in the main manuscript.

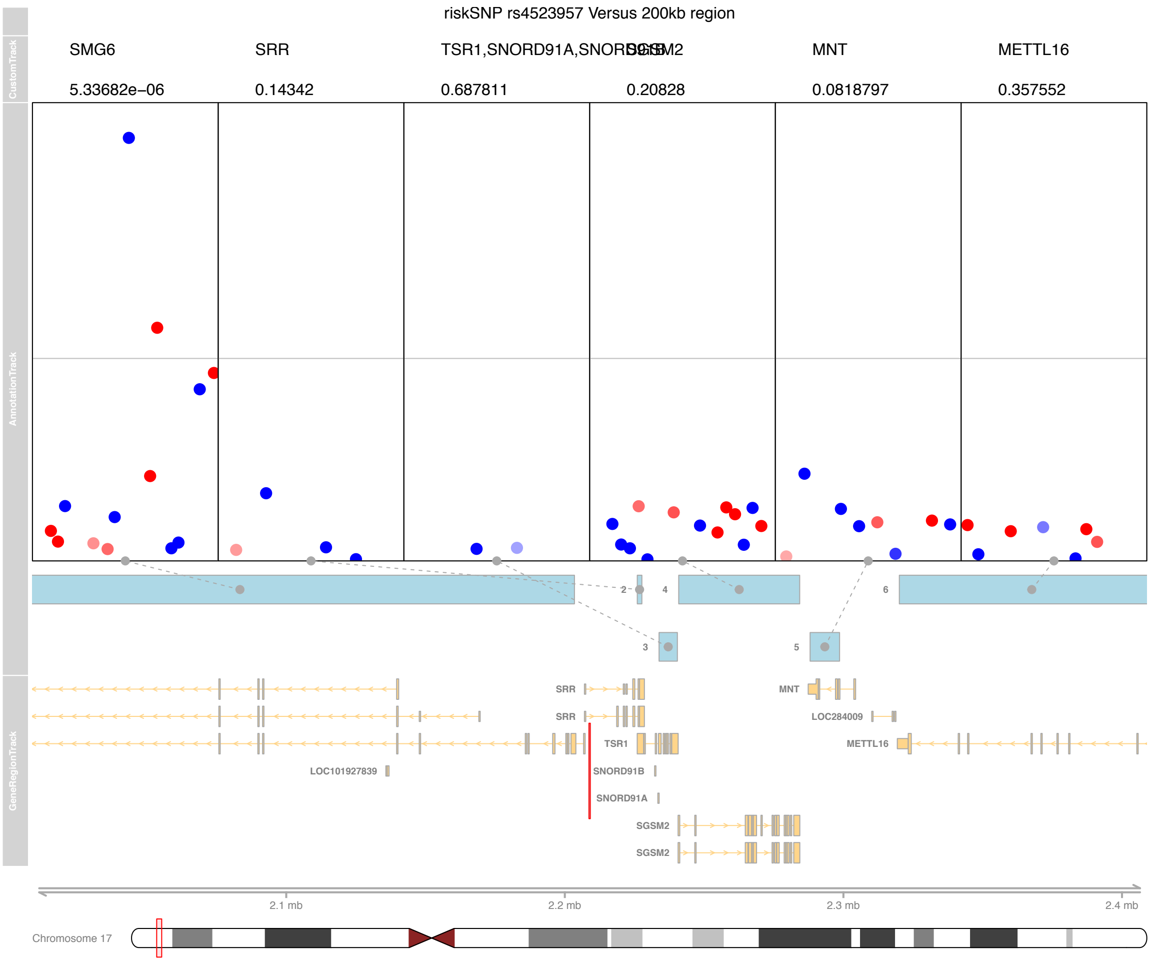

Figure S3-18. riskSNP-centric plot for DLPFC similar to figure 3 in the main manuscript.

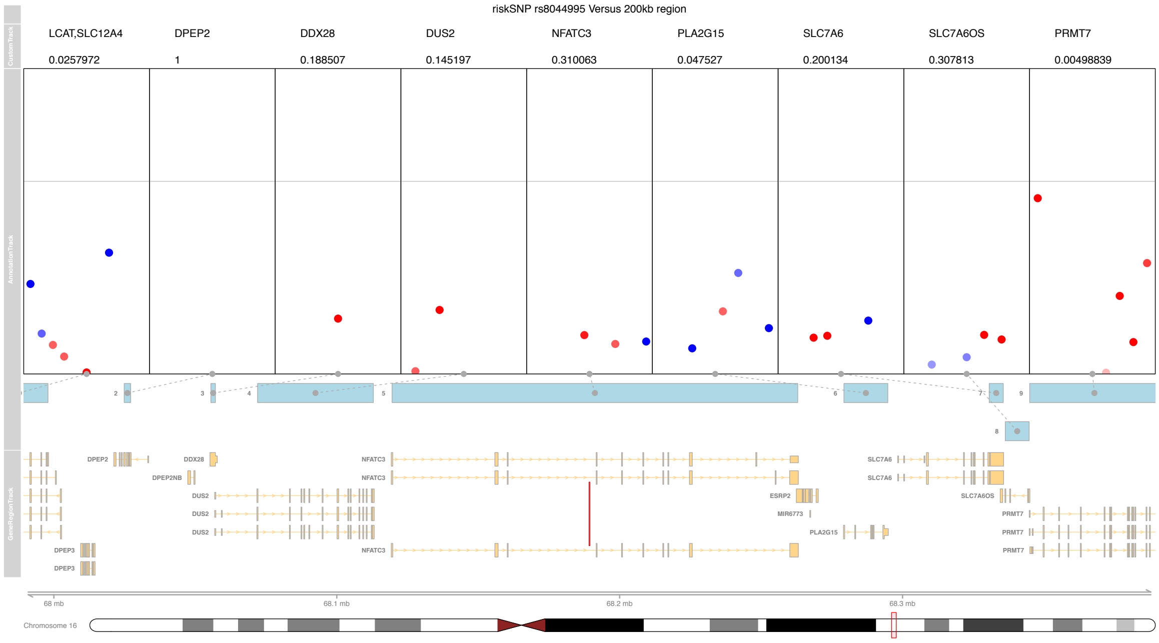

Figure S3-19. riskSNP-centric plot for DLPFC similar to figure 3 in the main manuscript.

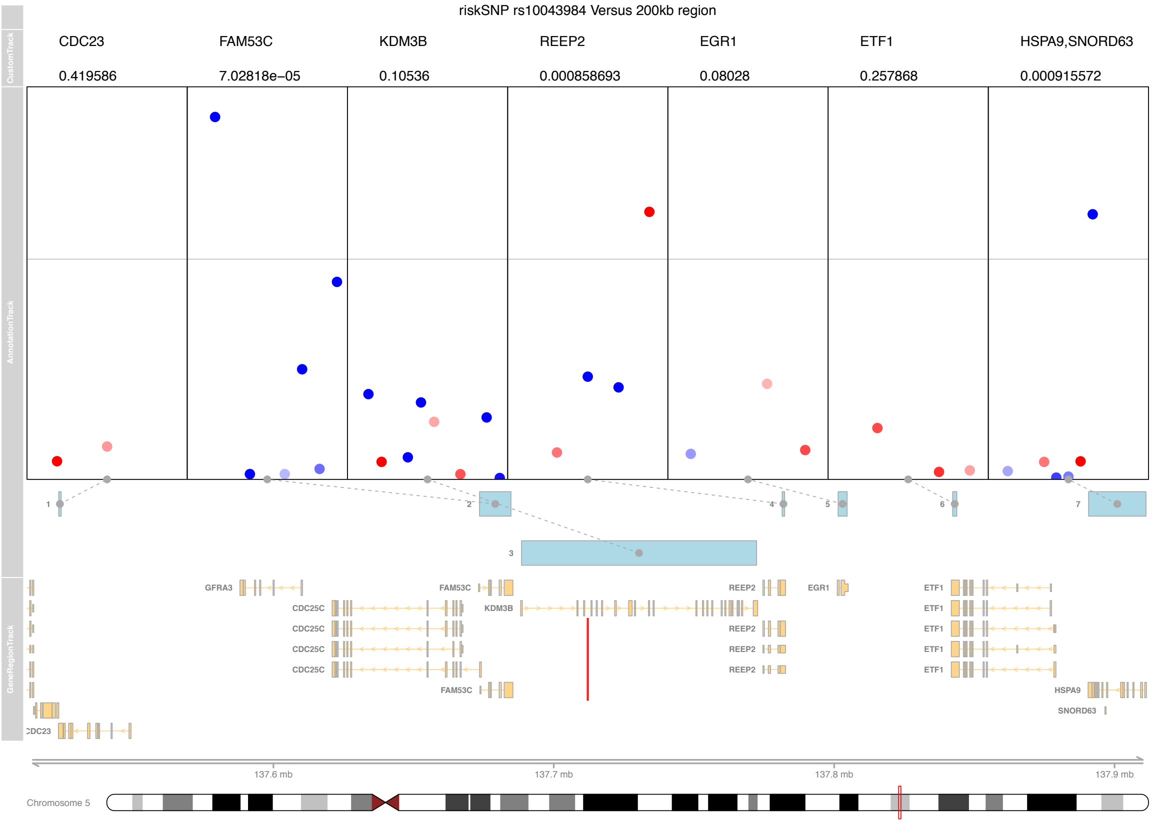

Figure S3-20. riskSNP-centric plot for DLPFC similar to figure 3 in the main manuscript.

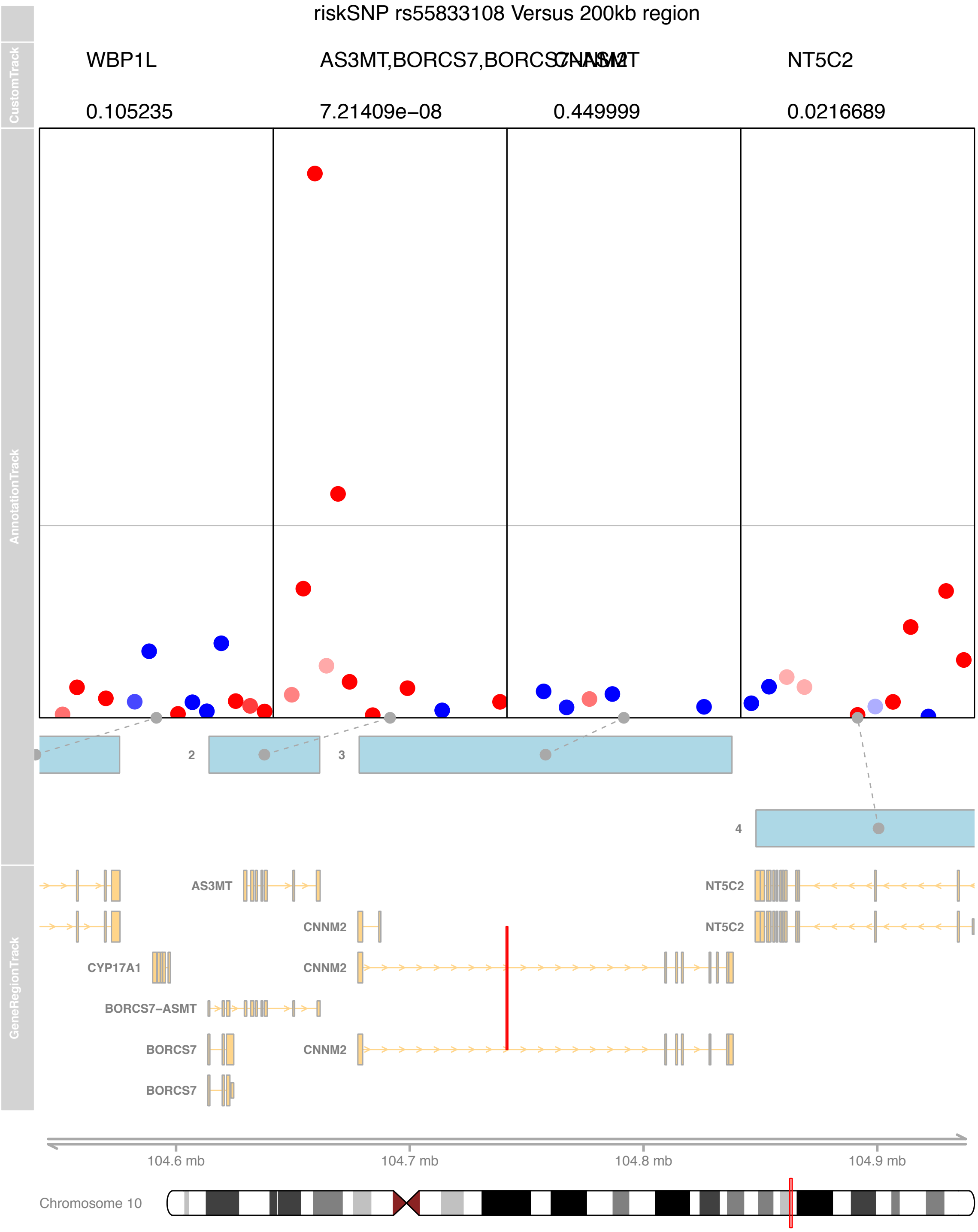

Figure S3-21. riskSNP-centric plot for DLPFC similar to figure 3 in the main manuscript.

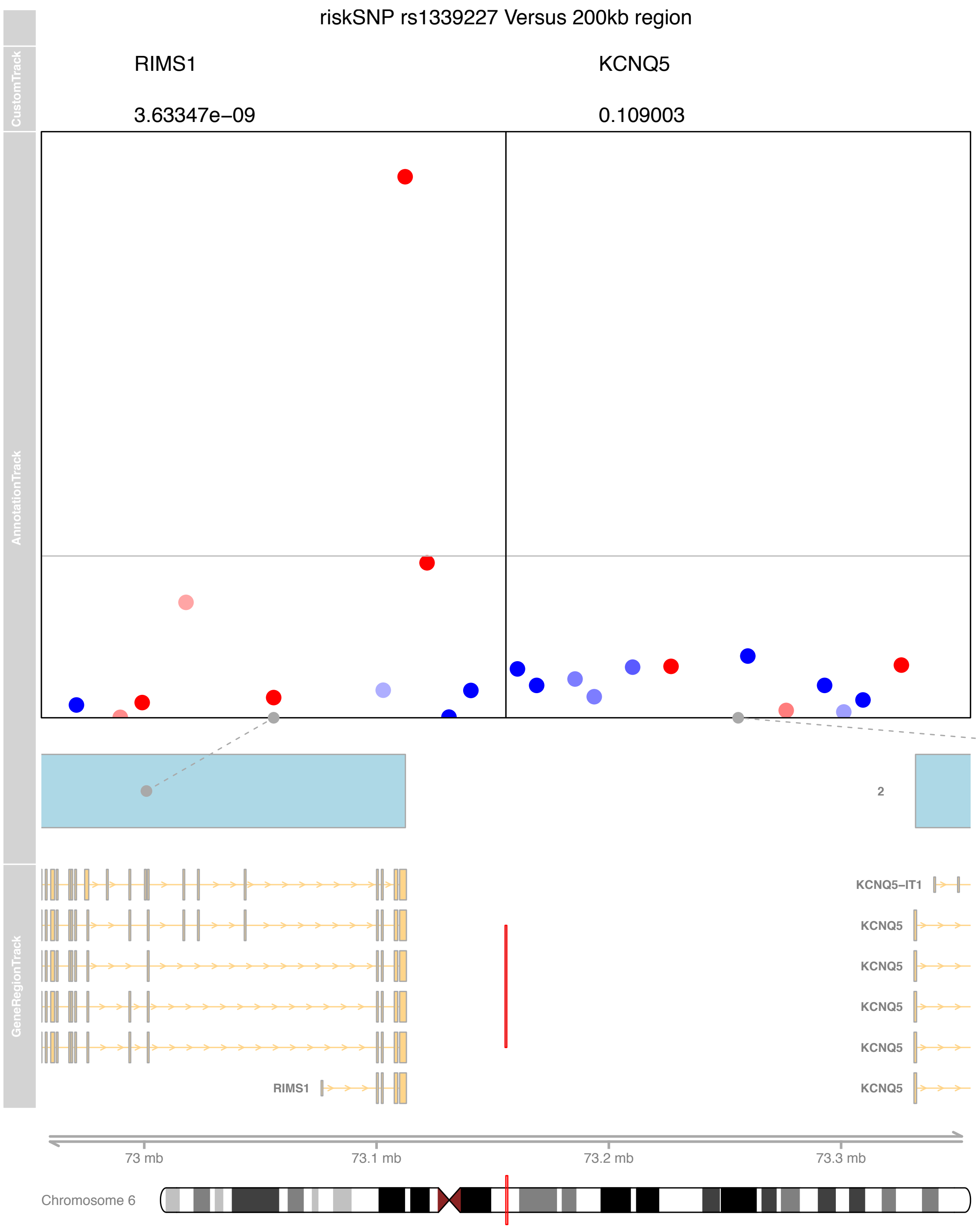

Figure S3-22. riskSNP-centric plot for DLPFC similar to figure 3 in the main manuscript.

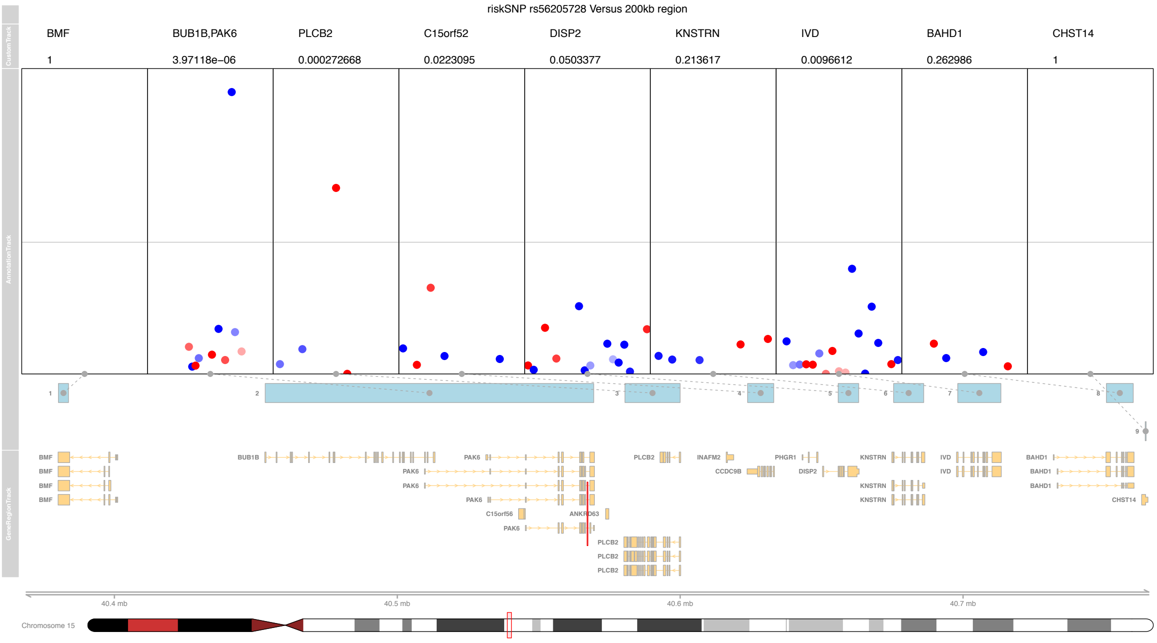

Figure S3-23. riskSNP-centric plot for DLPFC similar to figure 3 in the main manuscript.

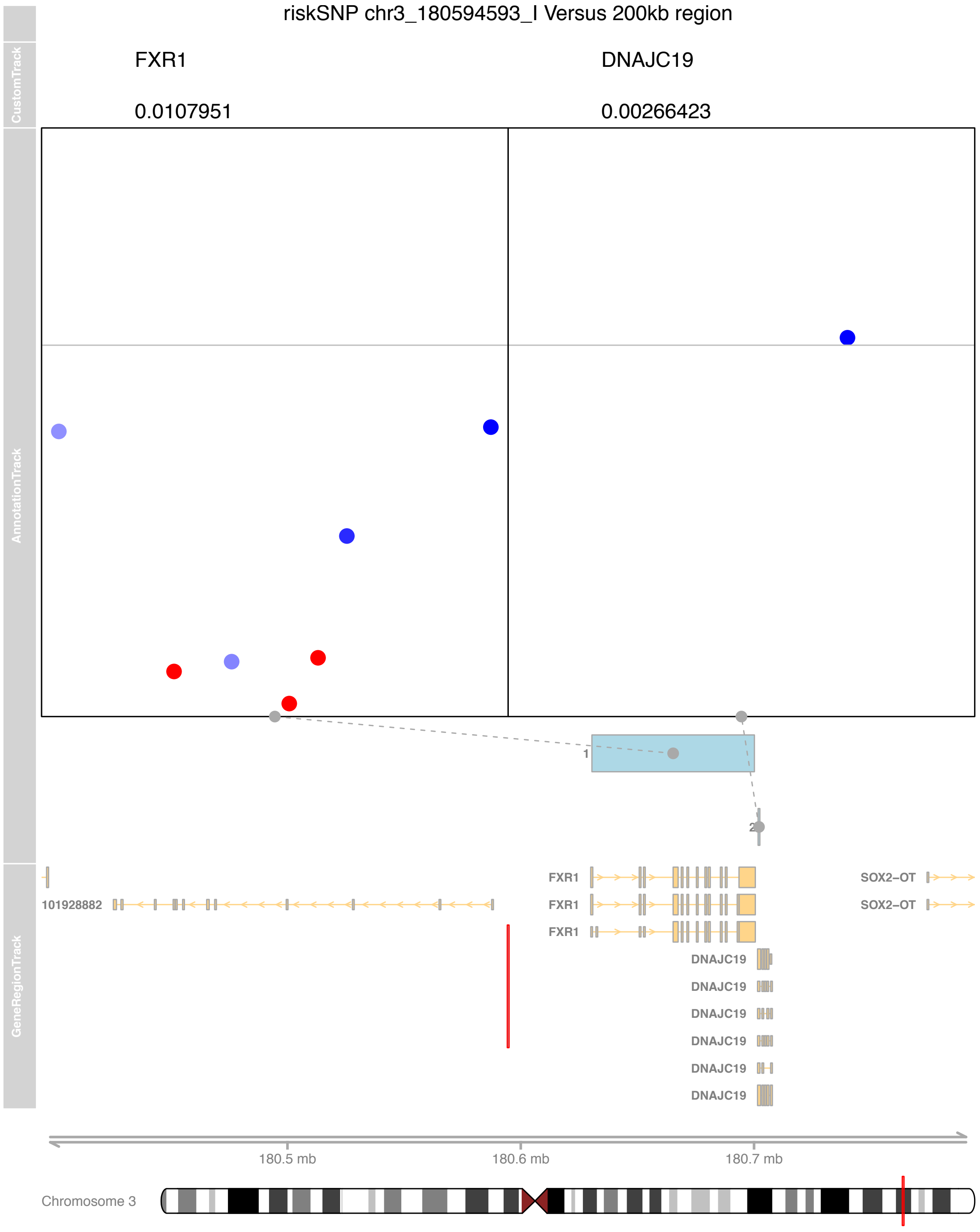

Figure S3-24. riskSNP-centric plot for DLPFC similar to figure 3 in the main manuscript.

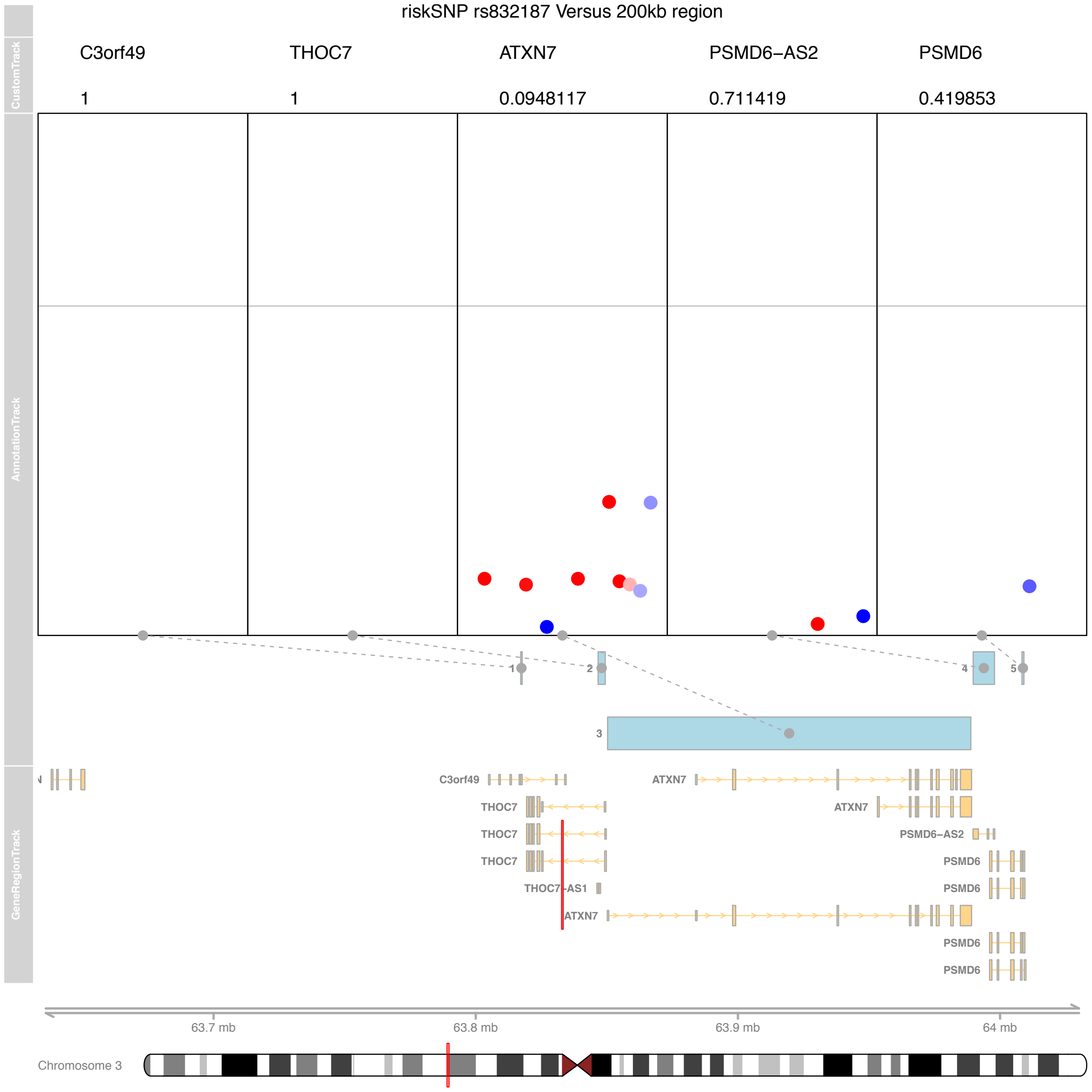

Figure S3-25. riskSNP-centric plot for DLPFC similar to figure 3 in the main manuscript.

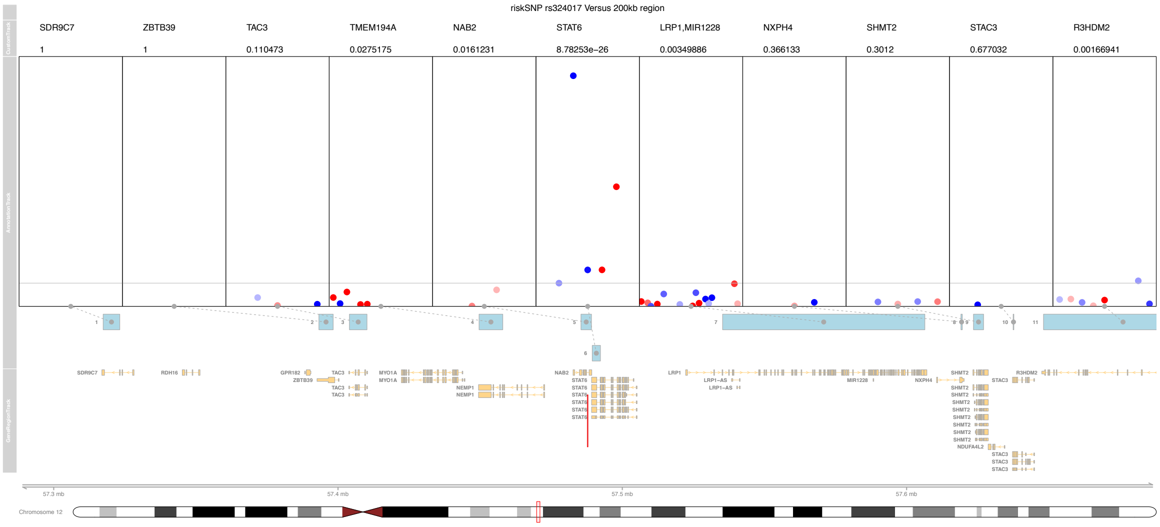

Figure S3-26. riskSNP-centric plot for DLPFC similar to figure 3 in the main manuscript.

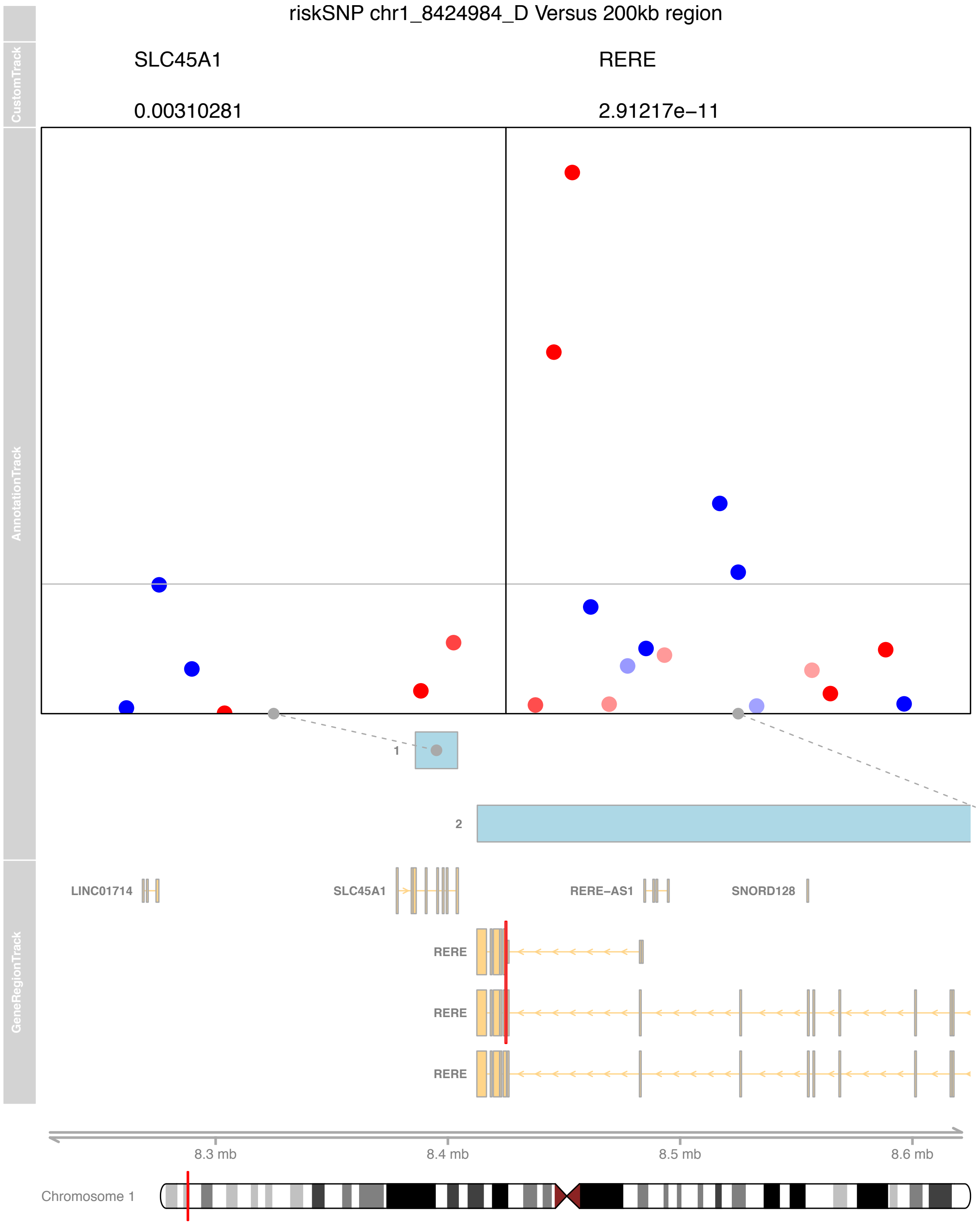

Figure S3-27. riskSNP-centric plot for DLPFC similar to figure 3 in the main manuscript.

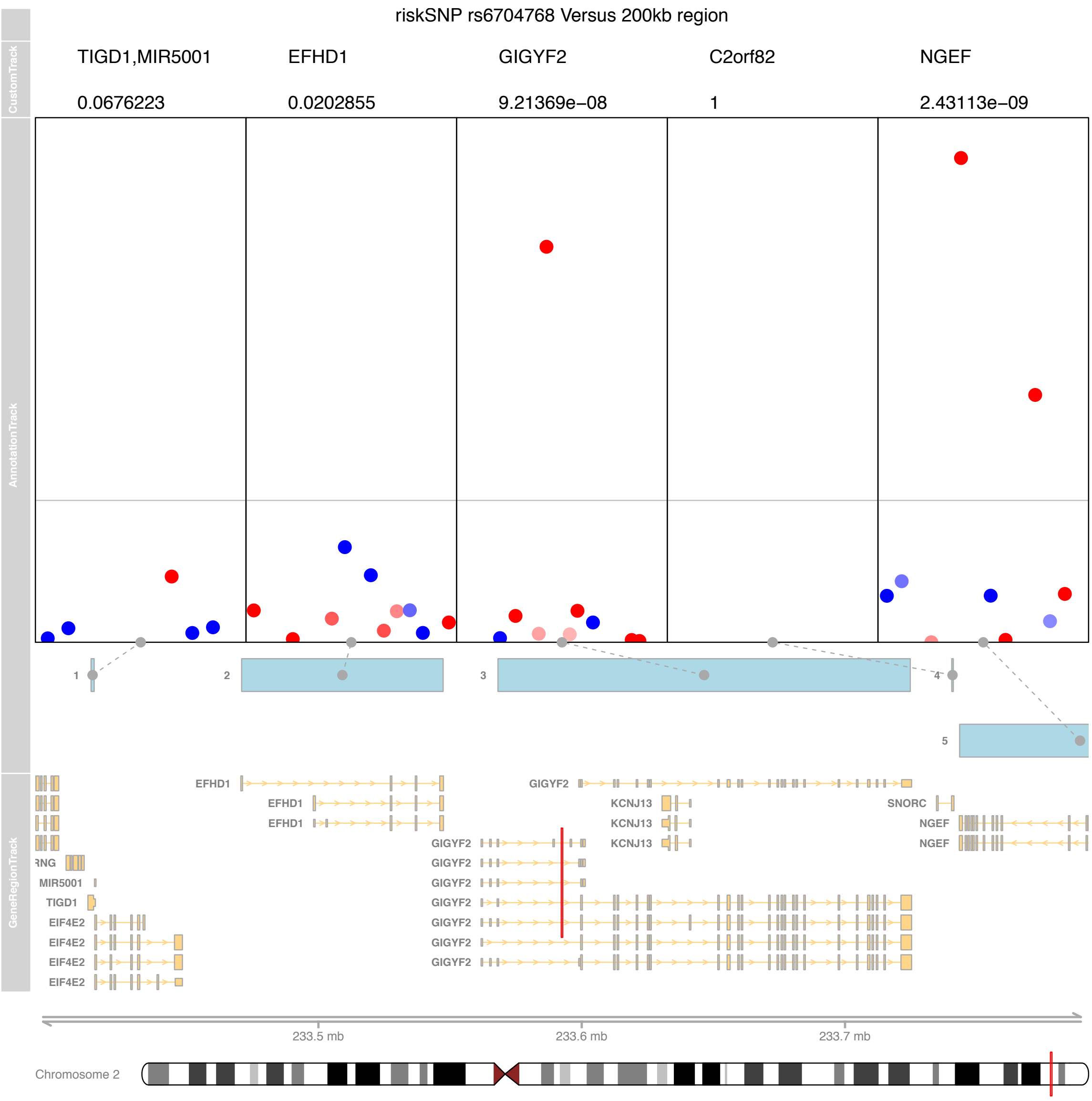

Figure S3-28. riskSNP-centric plot for DLPFC similar to figure 3 in the main manuscript.

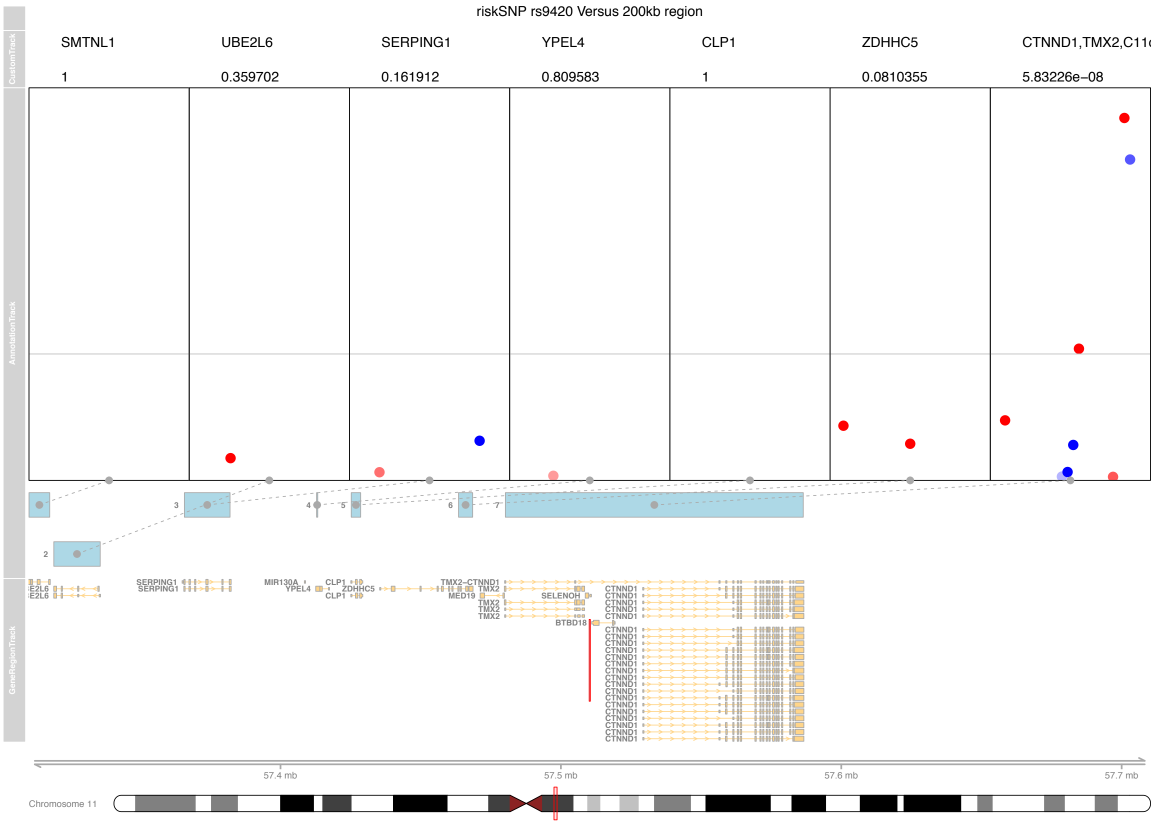

Figure S3-29. riskSNP-centric plot for DLPFC similar to figure 3 in the main manuscript.

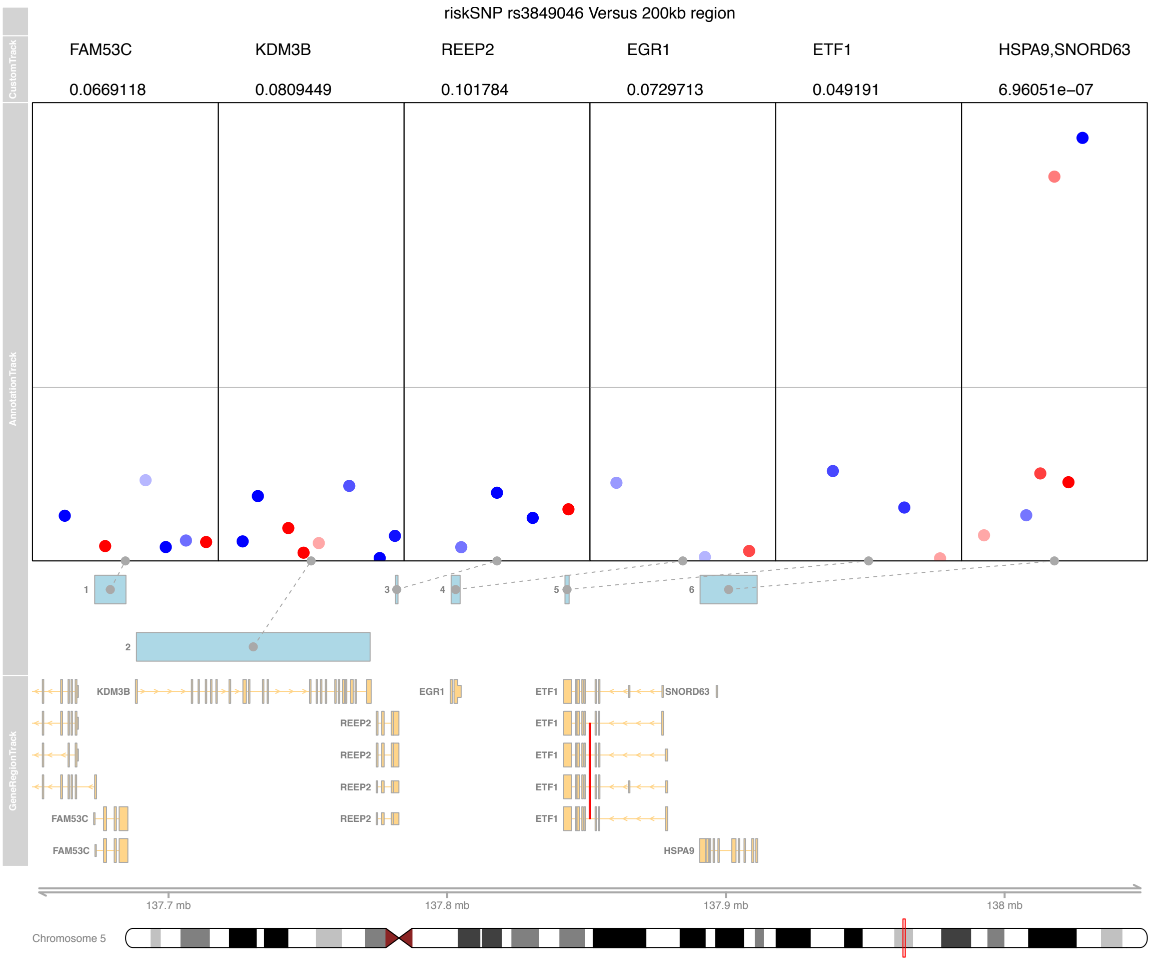

Figure S3-30. riskSNP-centric plot for DLPFC similar to figure 3 in the main manuscript.

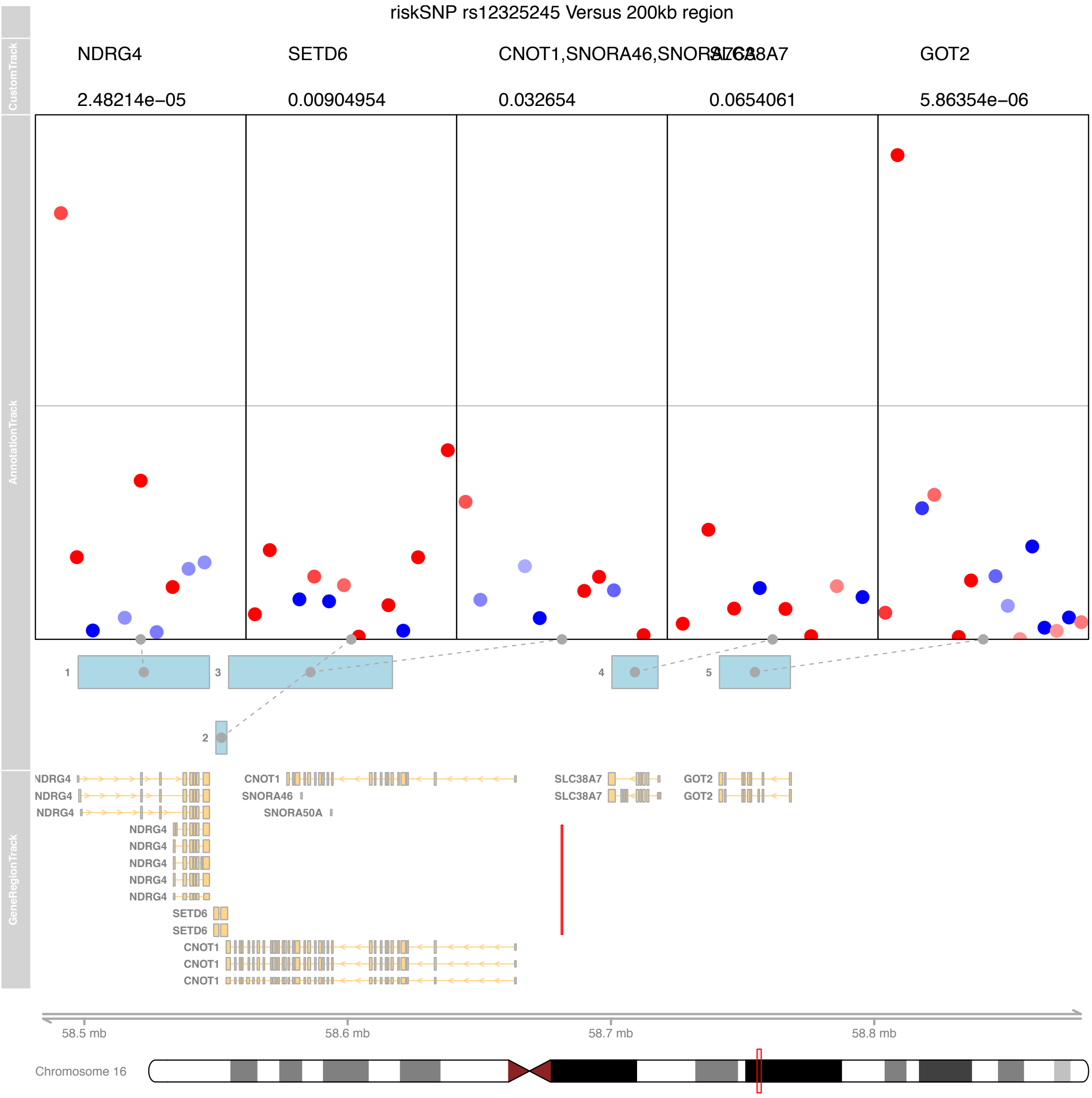

Figure S3-31. riskSNP-centric plot for DLPFC similar to figure 3 in the main manuscript.

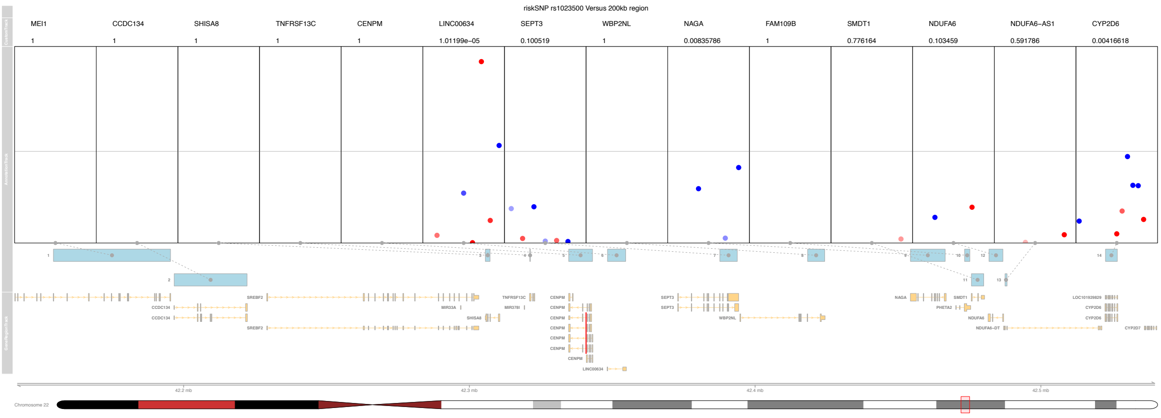

Figure S3-32. riskSNP-centric plot for DLPFC similar to figure 3 in the main manuscript.

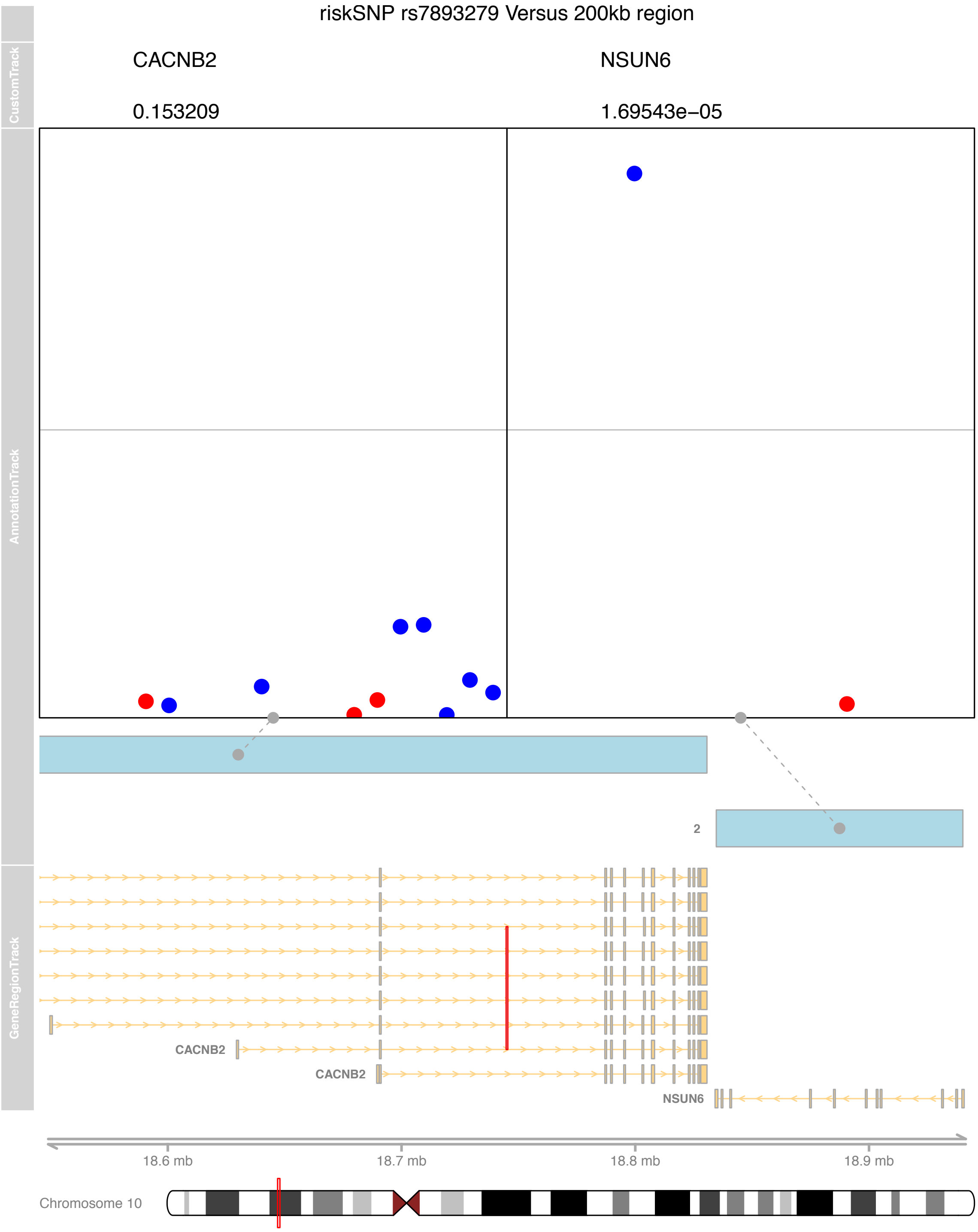

Figure S3-33. riskSNP-centric plot for DLPFC similar to figure 3 in the main manuscript.

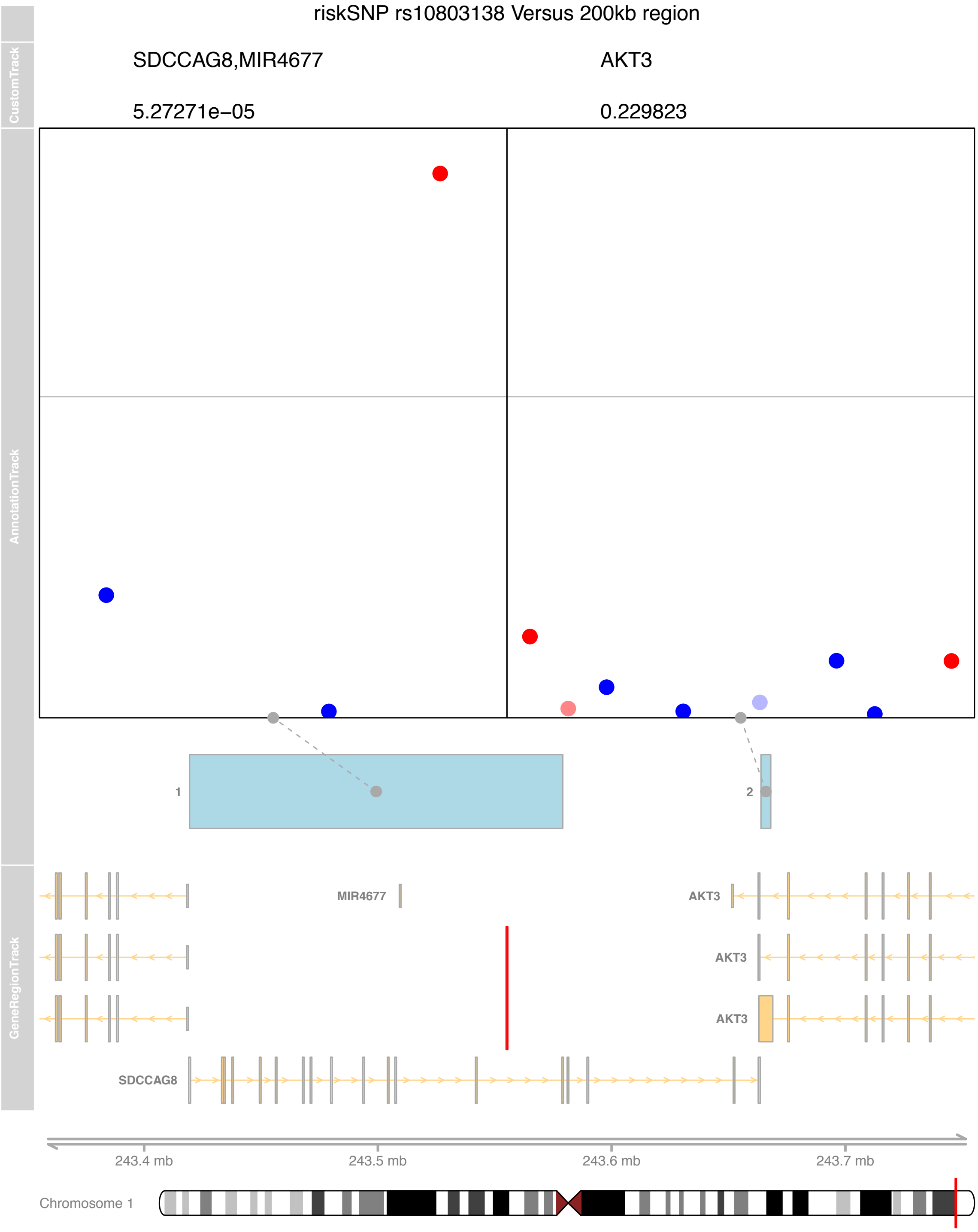

Figure S3-34. riskSNP-centric plot for DLPFC similar to figure 3 in the main manuscript.

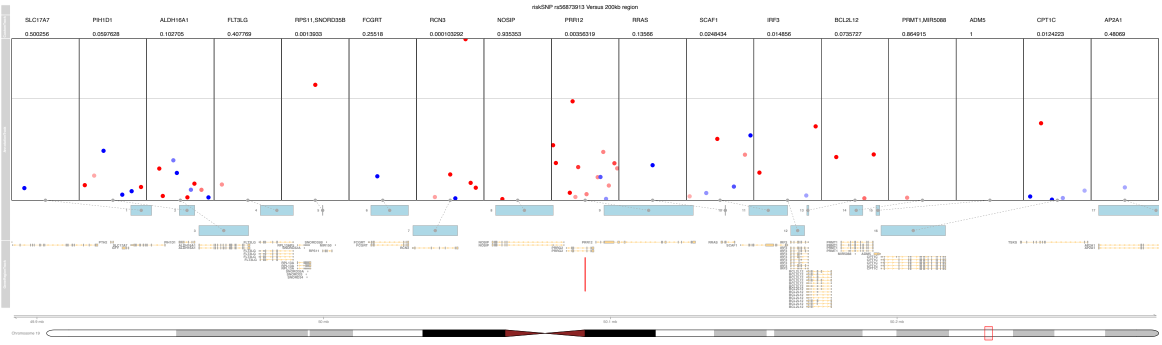

Figure S3-35. riskSNP-centric plot for DLPFC similar to figure 3 in the main manuscript.

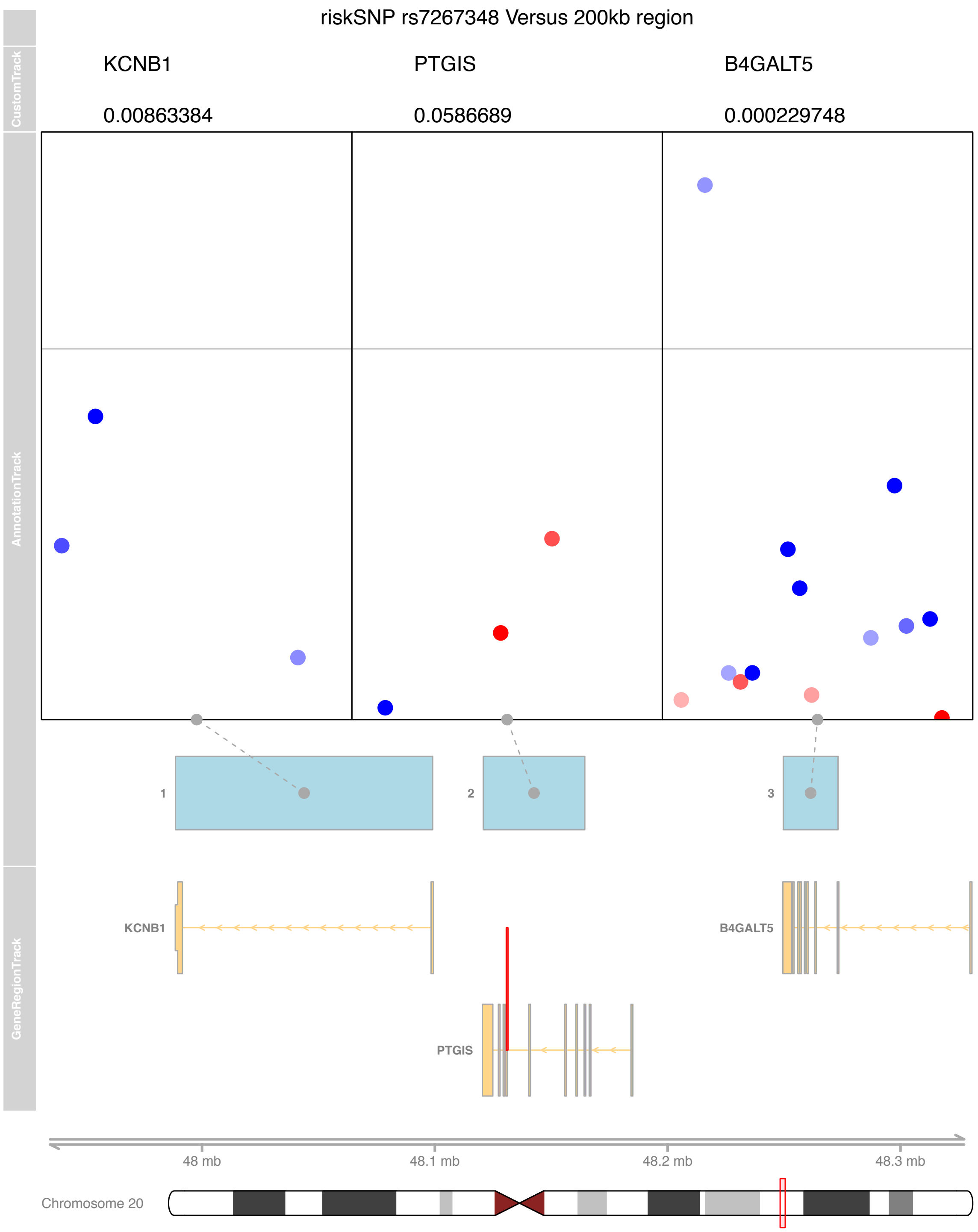

Figure S3-36. riskSNP-centric plot for DLPFC similar to figure 3 in the main manuscript.

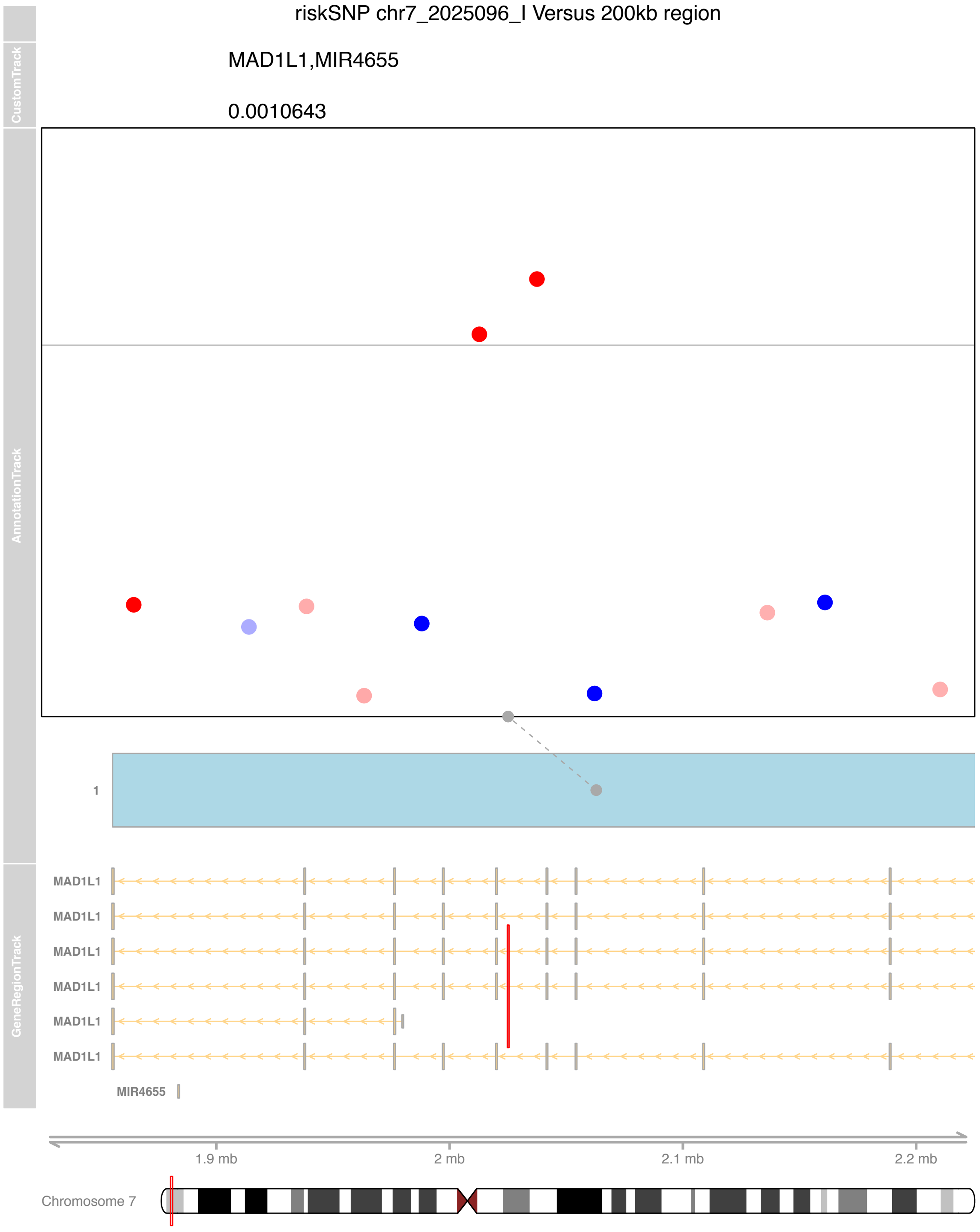

Figure S3-37. riskSNP-centric plot for DLPFC similar to figure 3 in the main manuscript.

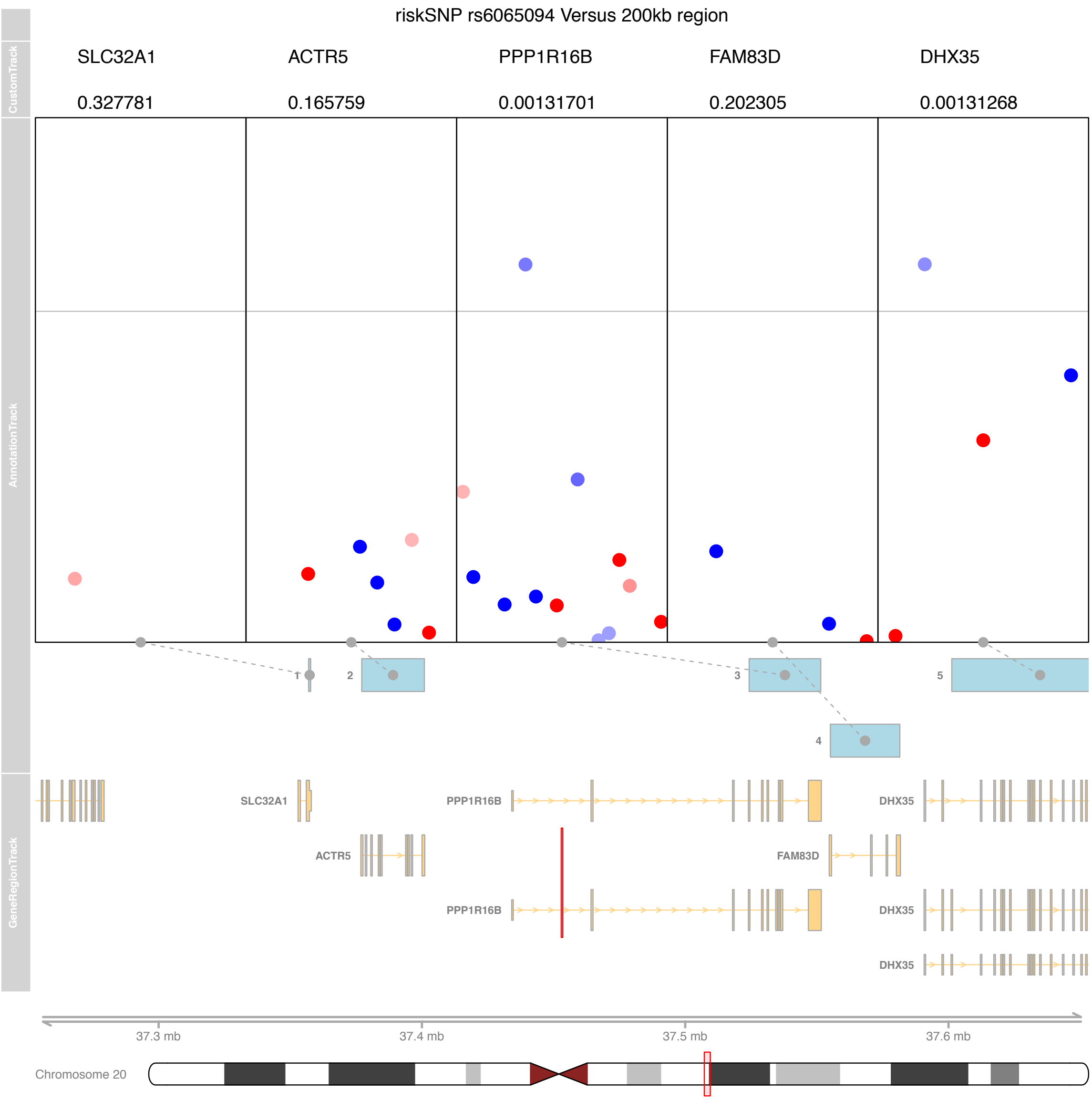

Figure S3-38. riskSNP-centric plot for DLPFC similar to figure 3 in the main manuscript.

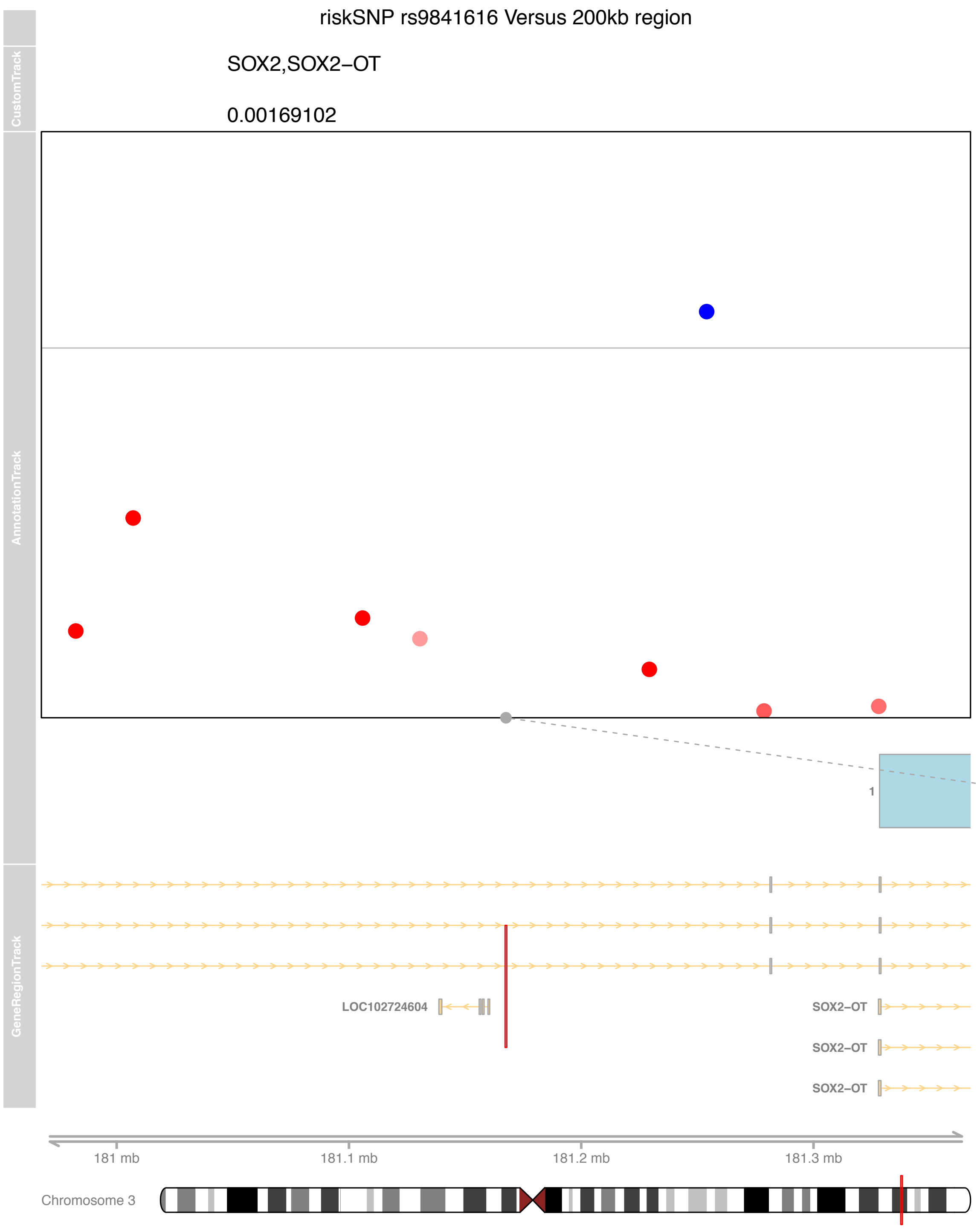

Figure S3-39. riskSNP-centric plot for DLPFC similar to figure 3 in the main manuscript.

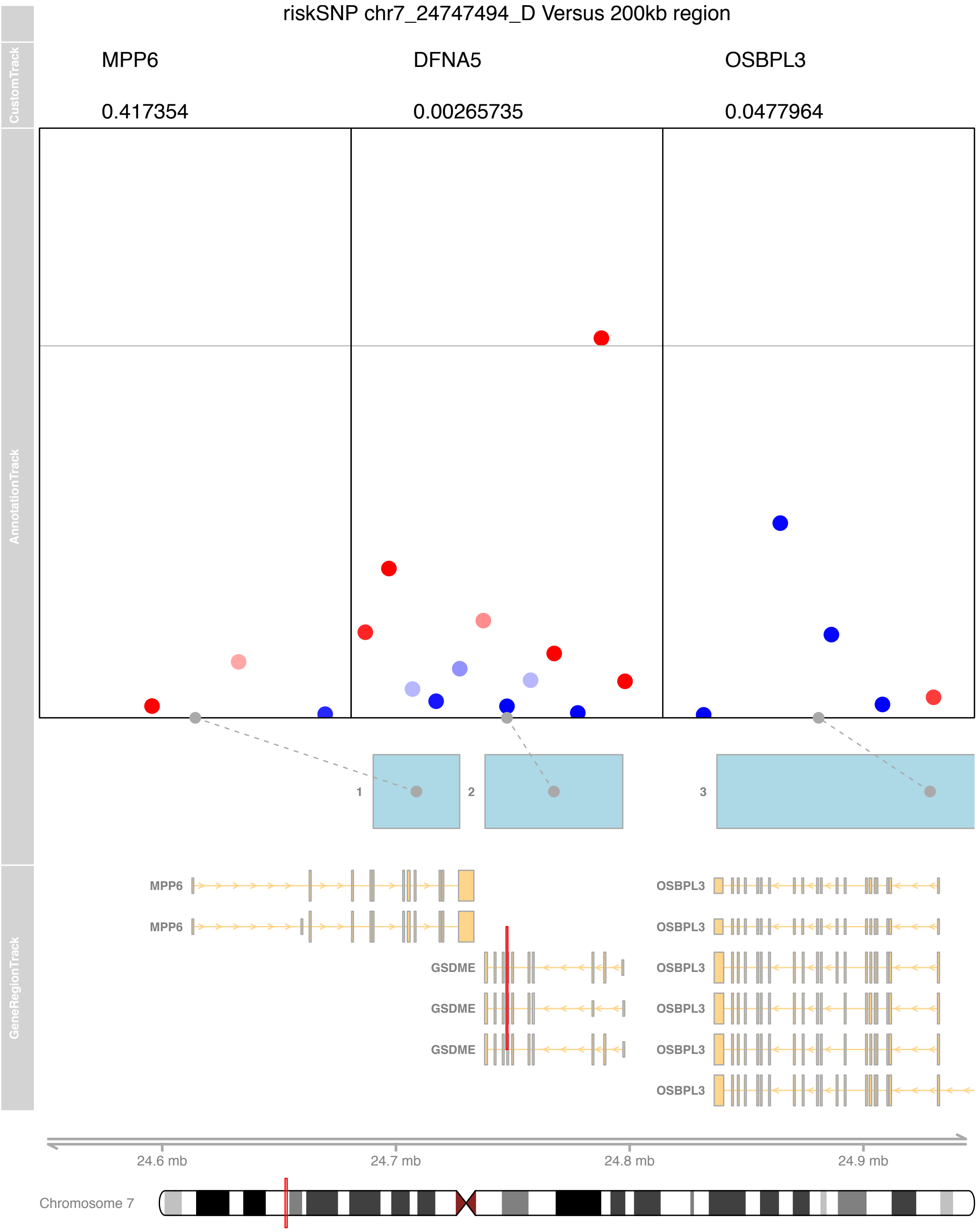

Figure S3-40. riskSNP-centric plot for DLPFC similar to figure 3 in the main manuscript.

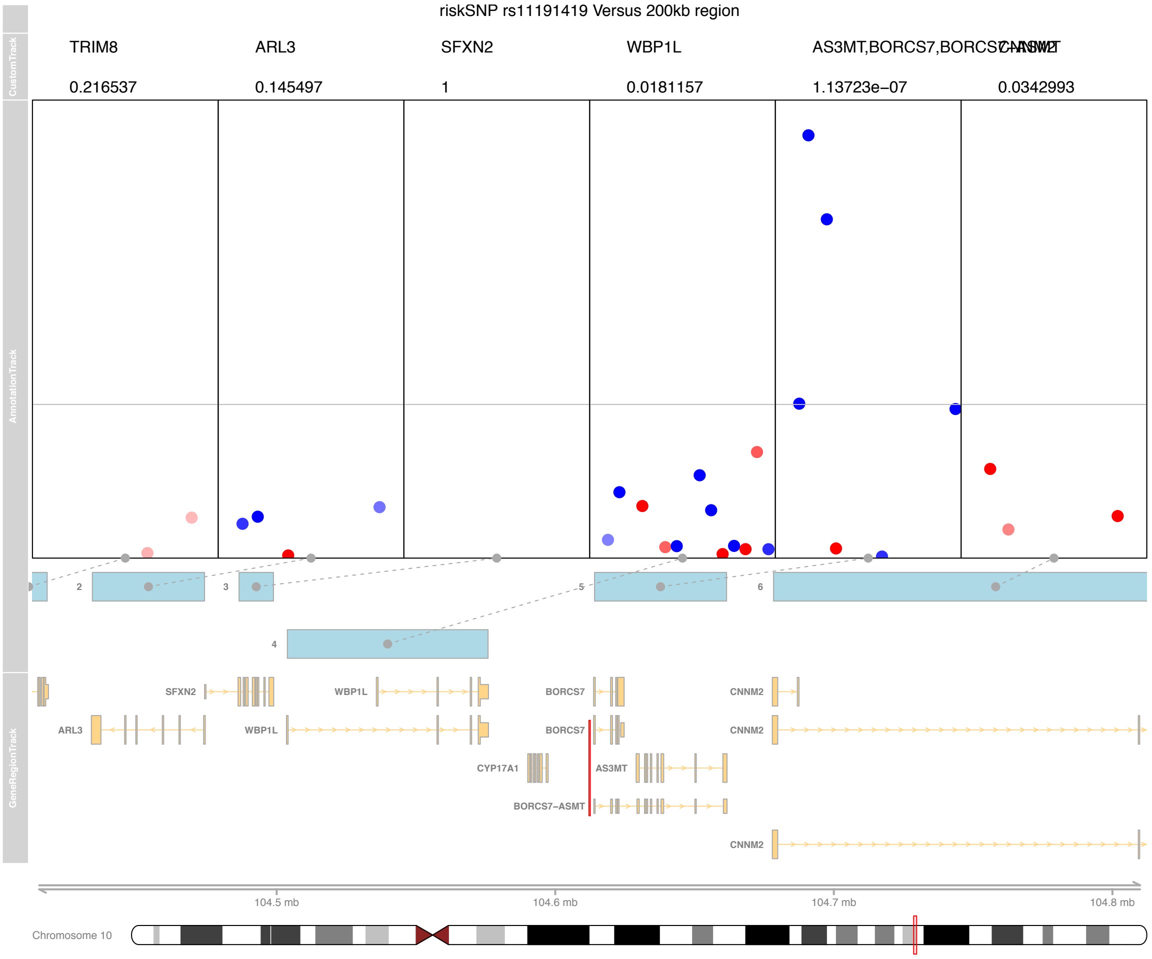

Figure S3-41. riskSNP-centric plot for Hippocampus similar to figure 3 in the main manuscript.

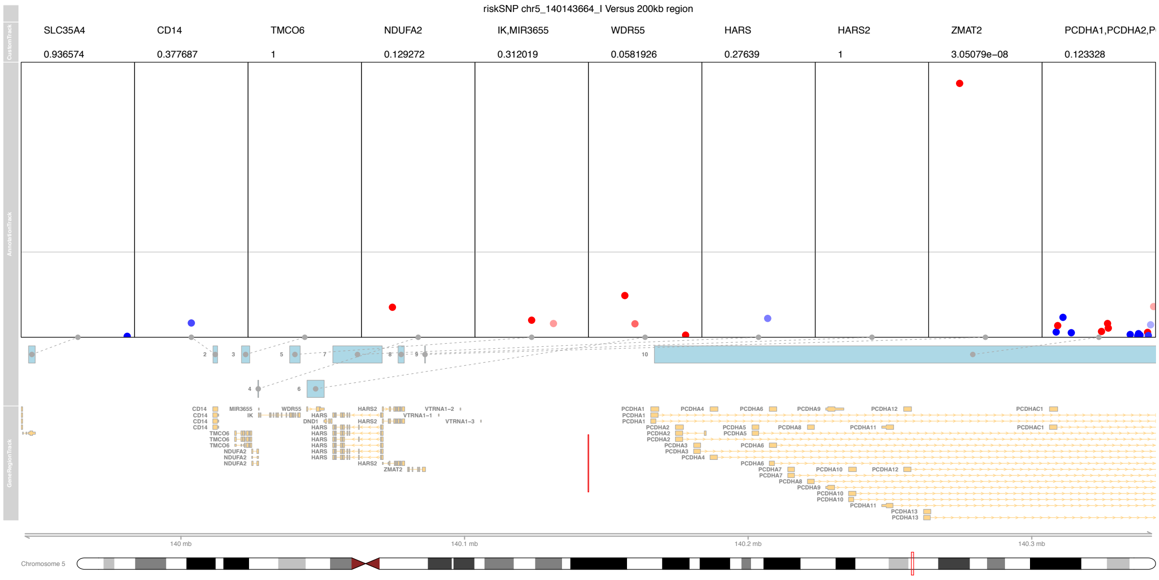

Figure S3-42. riskSNP-centric plot for Hippocampus similar to figure 3 in the main manuscript.

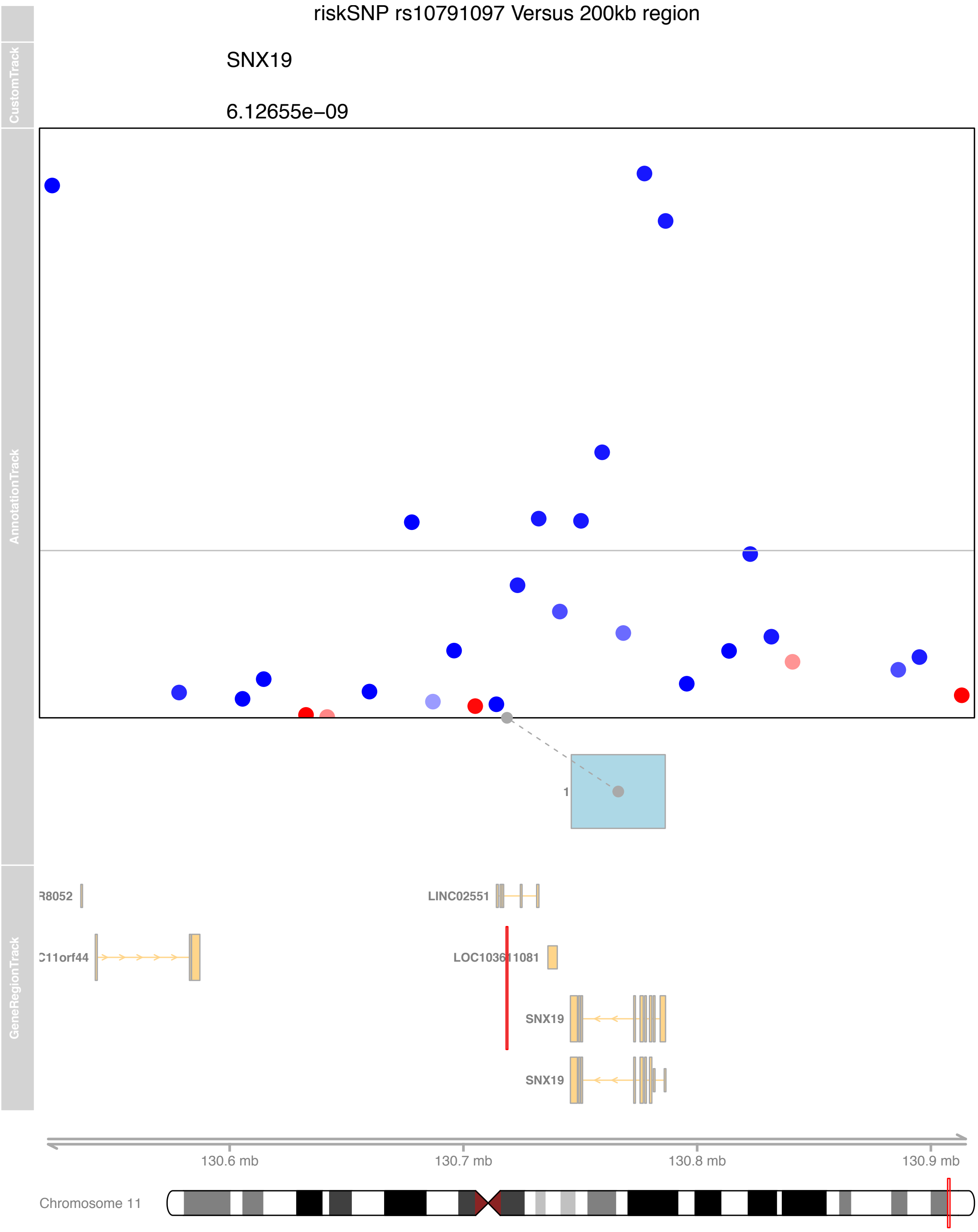

Figure S3-43. riskSNP-centric plot for Hippocampus similar to figure 3 in the main manuscript.

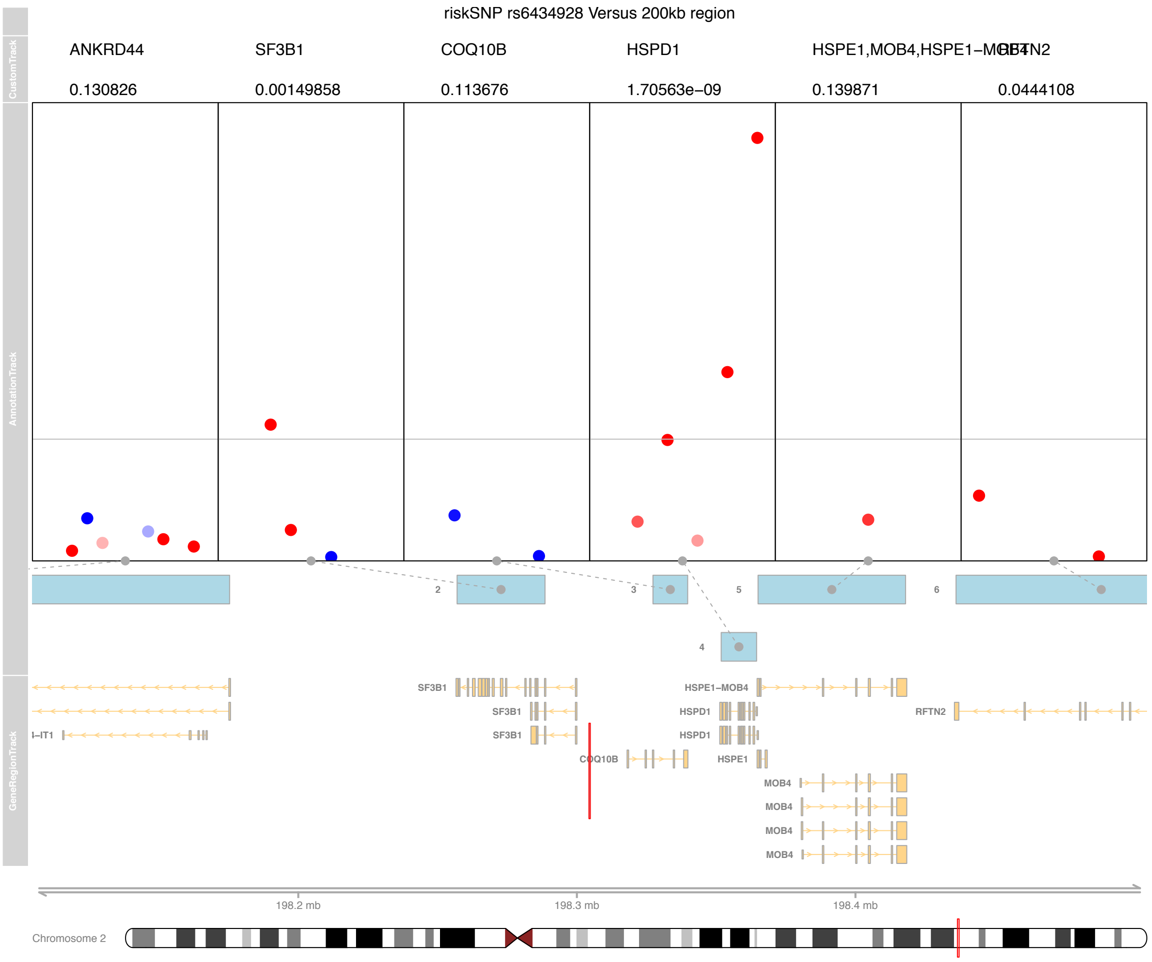

Figure S3-44. riskSNP-centric plot for Hippocampus similar to figure 3 in the main manuscript.

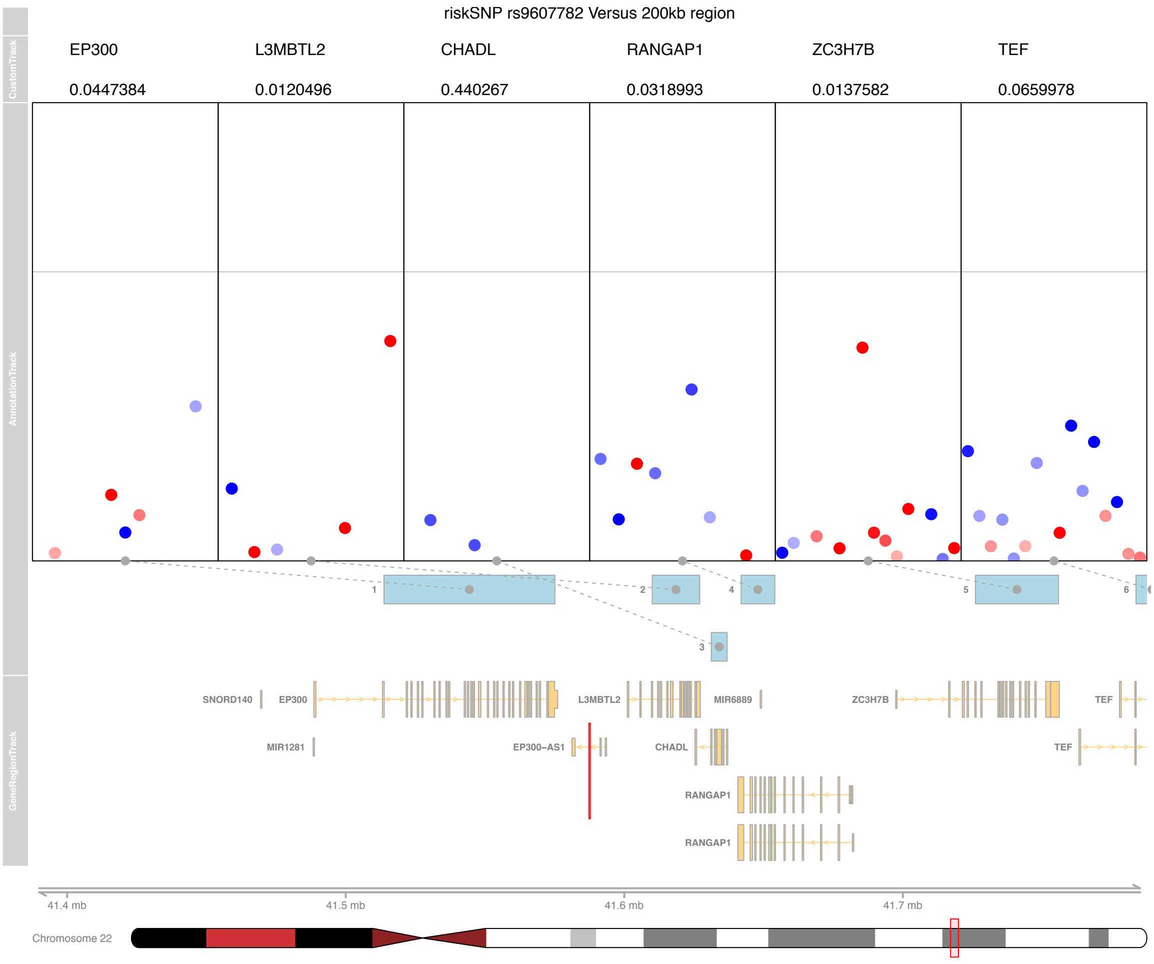

Figure S3-45. riskSNP-centric plot for Hippocampus similar to figure 3 in the main manuscript.

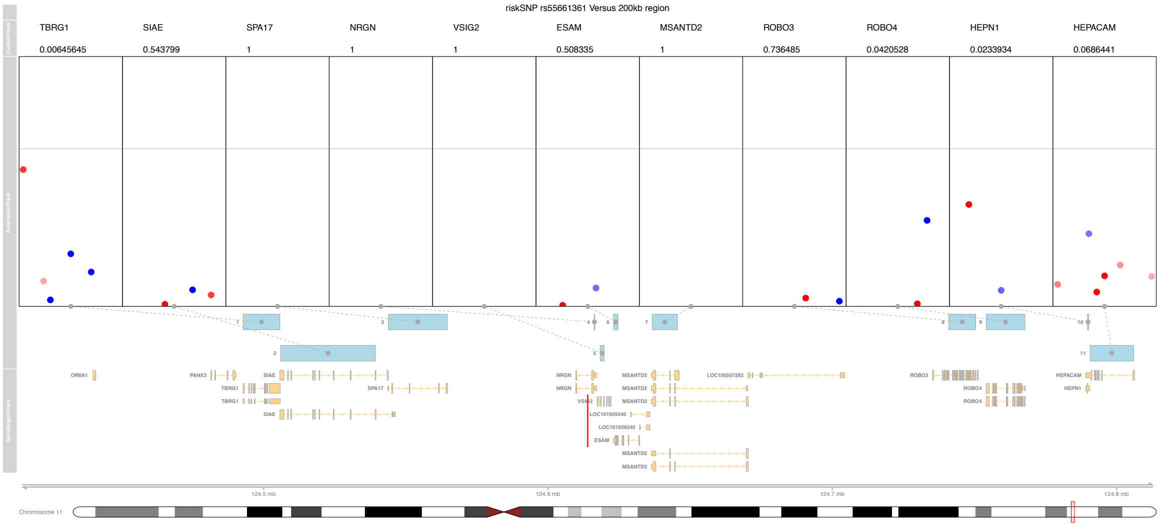

Figure S3-46. riskSNP-centric plot for Hippocampus similar to figure 3 in the main manuscript.

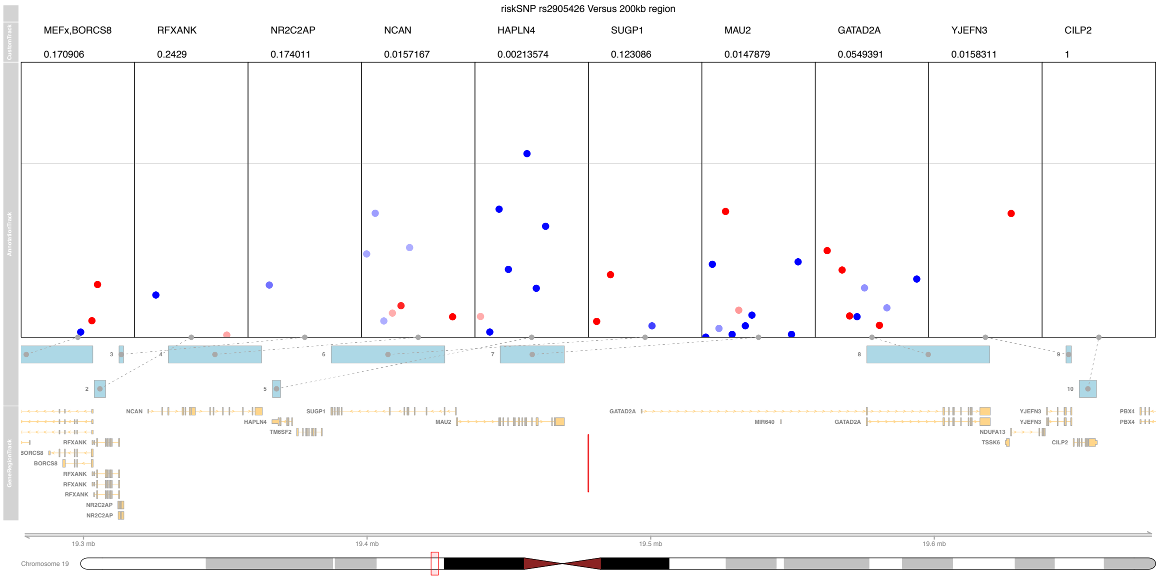

Figure S3-47. riskSNP-centric plot for Hippocampus similar to figure 3 in the main manuscript.

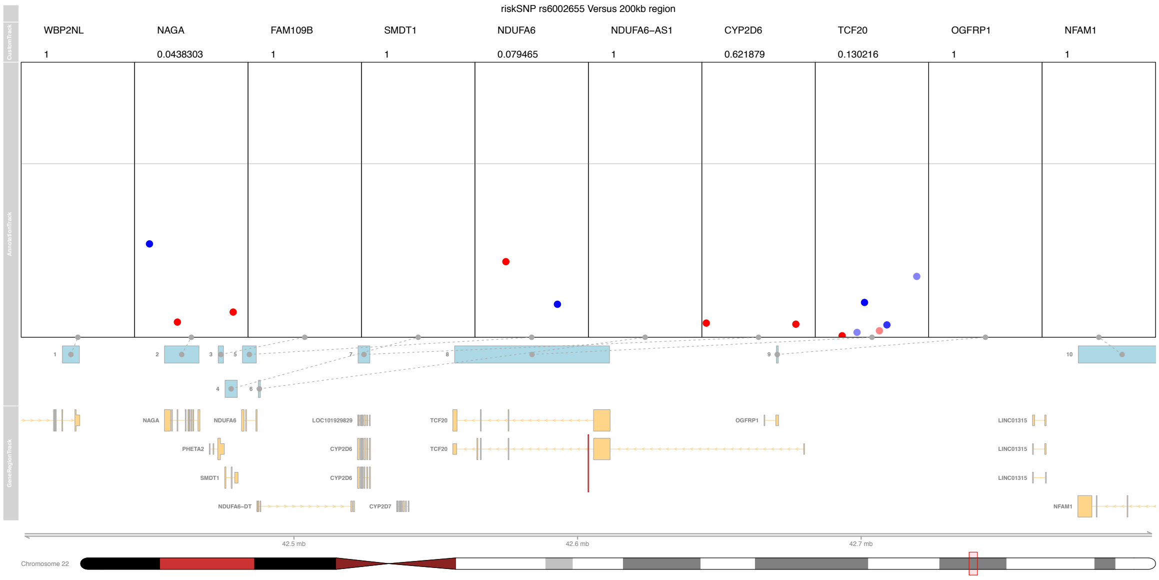

Figure S3-48. riskSNP-centric plot for Hippocampus similar to figure 3 in the main manuscript.

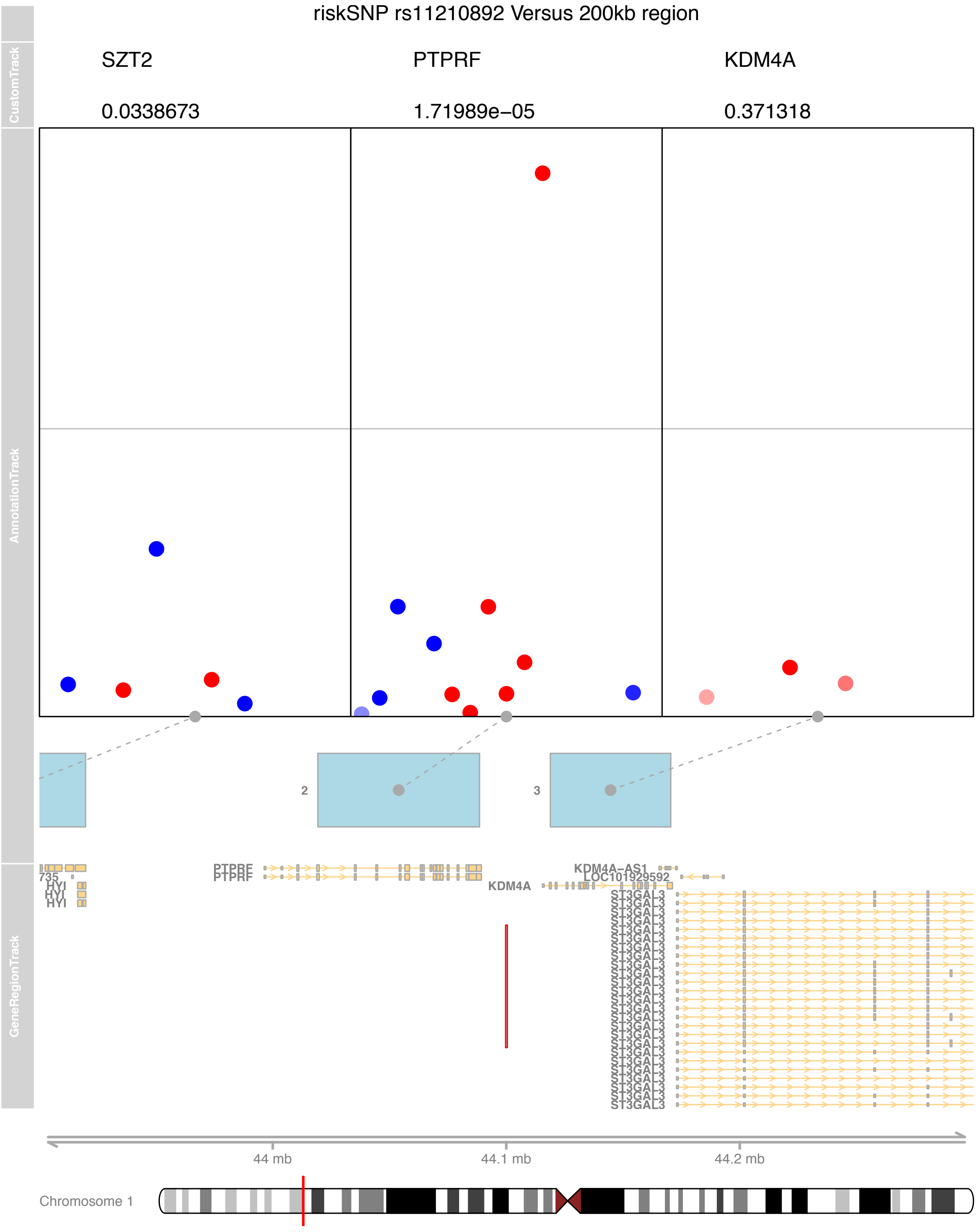

Figure S3-49. riskSNP-centric plot for Hippocampus similar to figure 3 in the main manuscript.

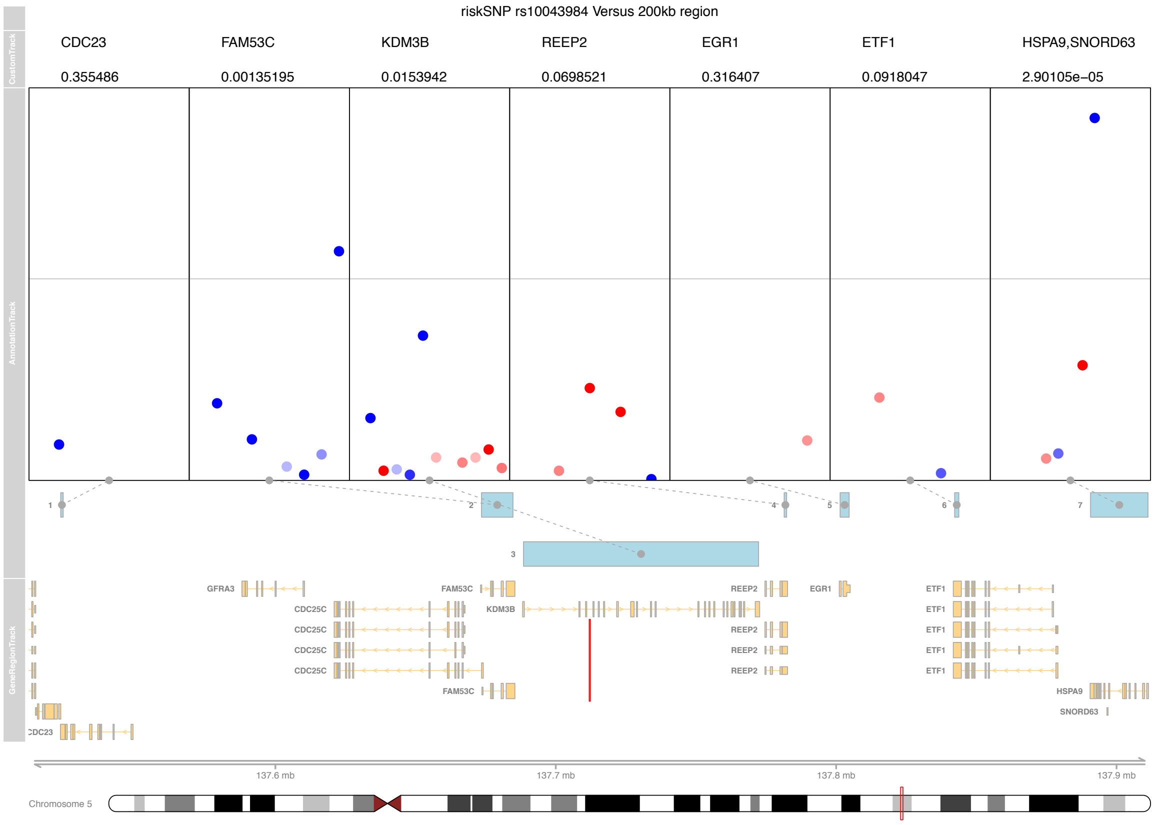

Figure S3-50. riskSNP-centric plot for Hippocampus similar to figure 3 in the main manuscript.

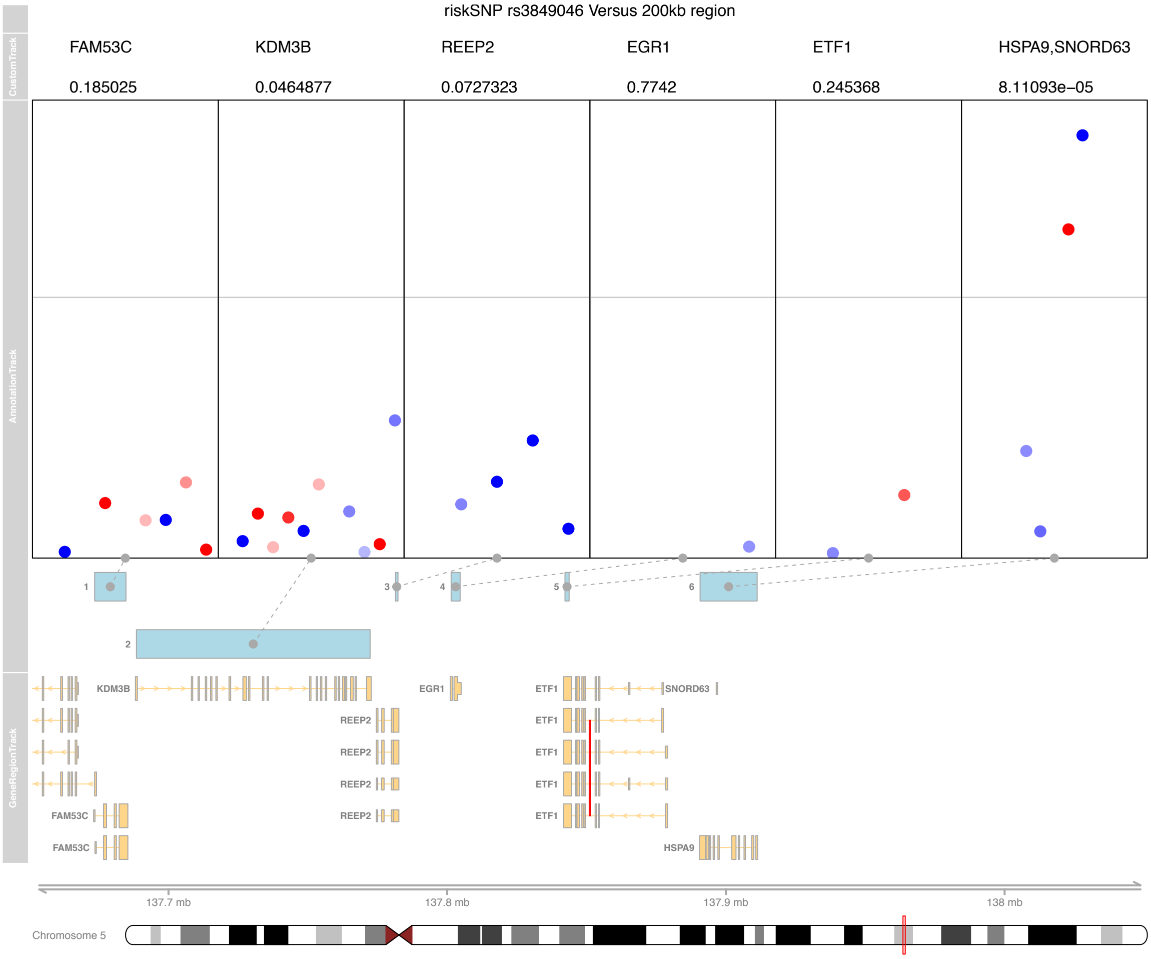

Figure S3-51. riskSNP-centric plot for Hippocampus similar to figure 3 in the main manuscript.

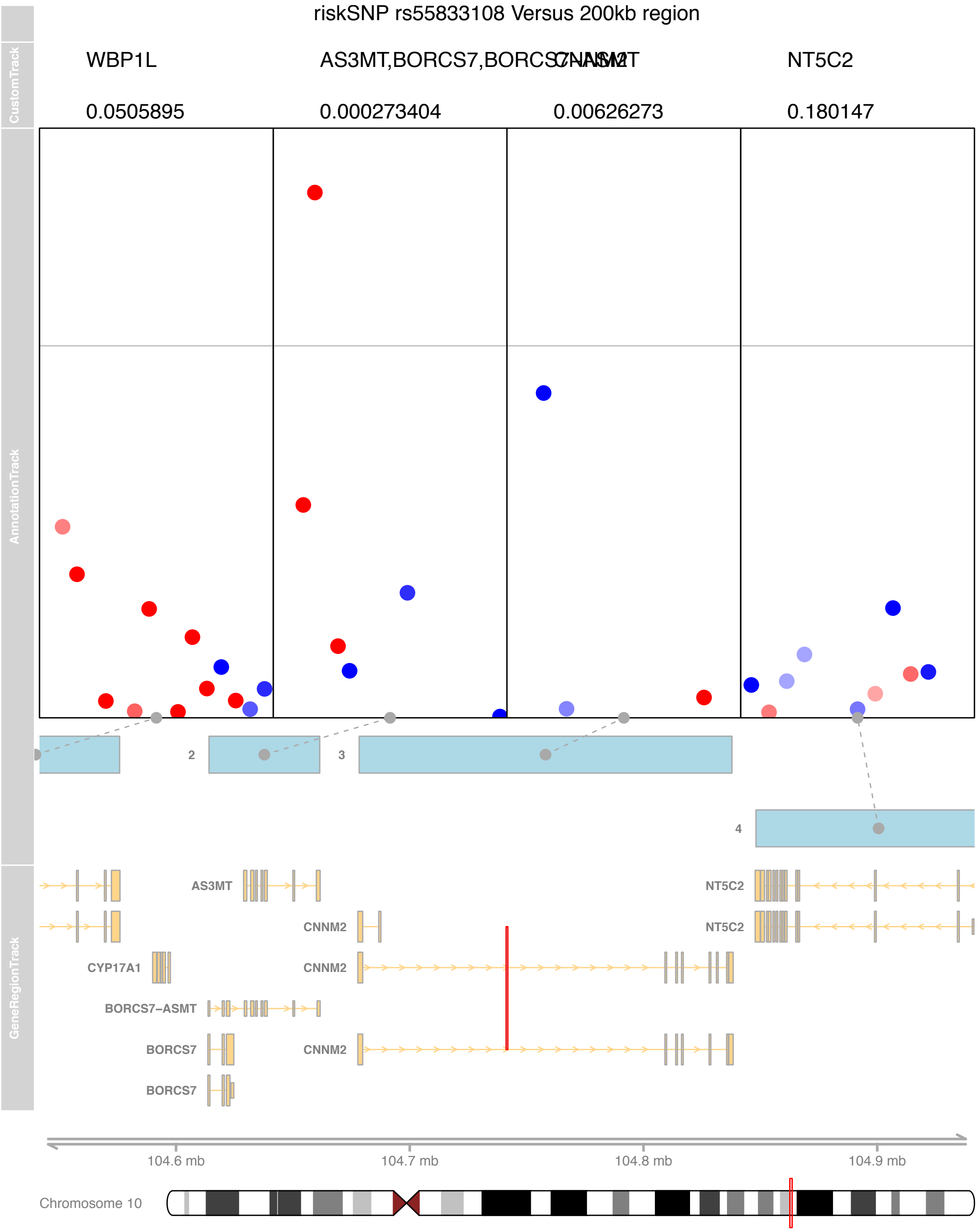

Figure S3-52. riskSNP-centric plot for Hippocampus similar to figure 3 in the main manuscript.

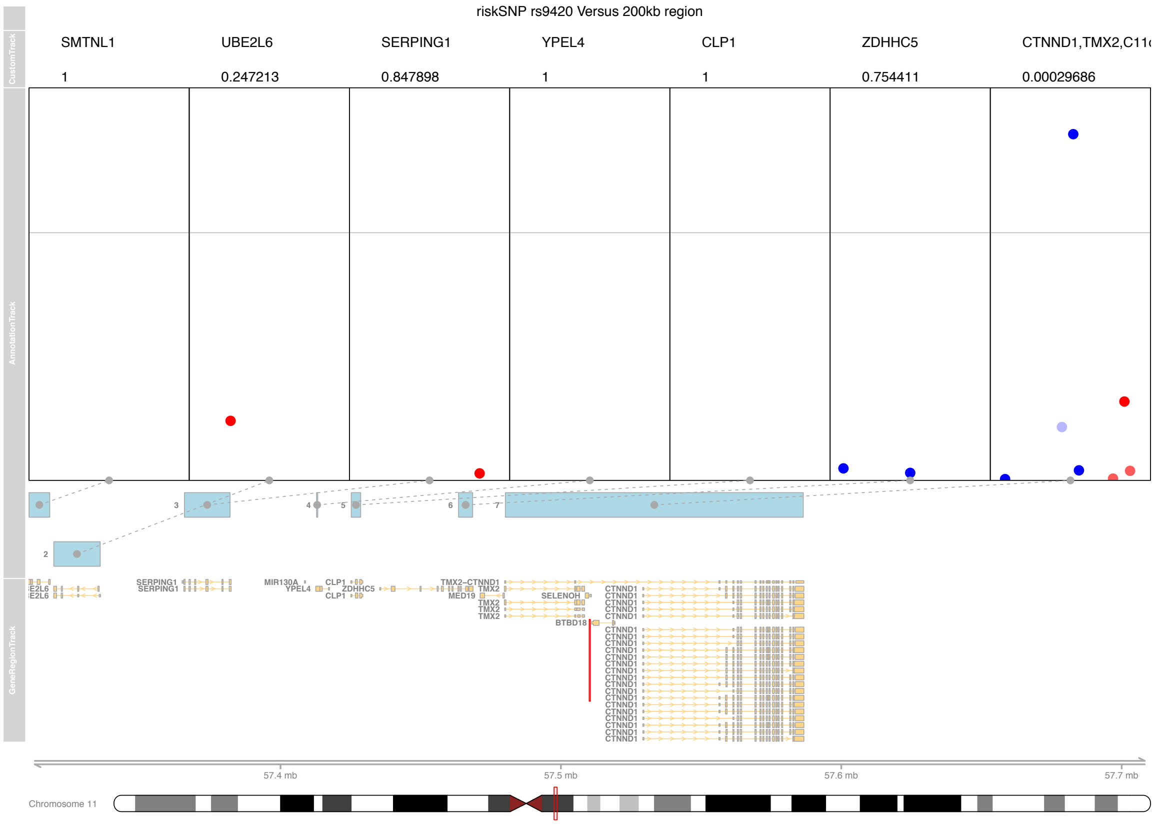

Figure S3-53. riskSNP-centric plot for Hippocampus similar to figure 3 in the main manuscript.

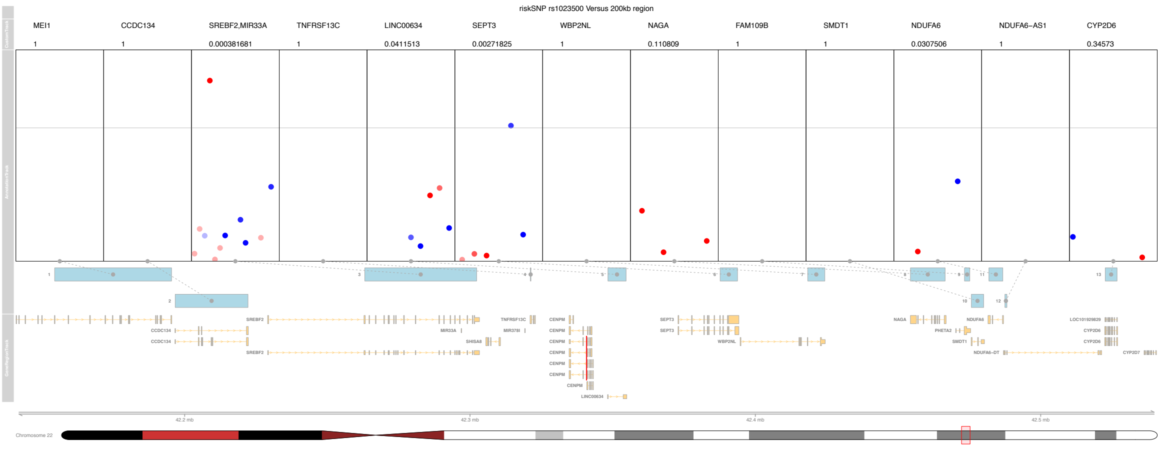

Figure S3-54. riskSNP-centric plot for Hippocampus similar to figure 3 in the main manuscript.

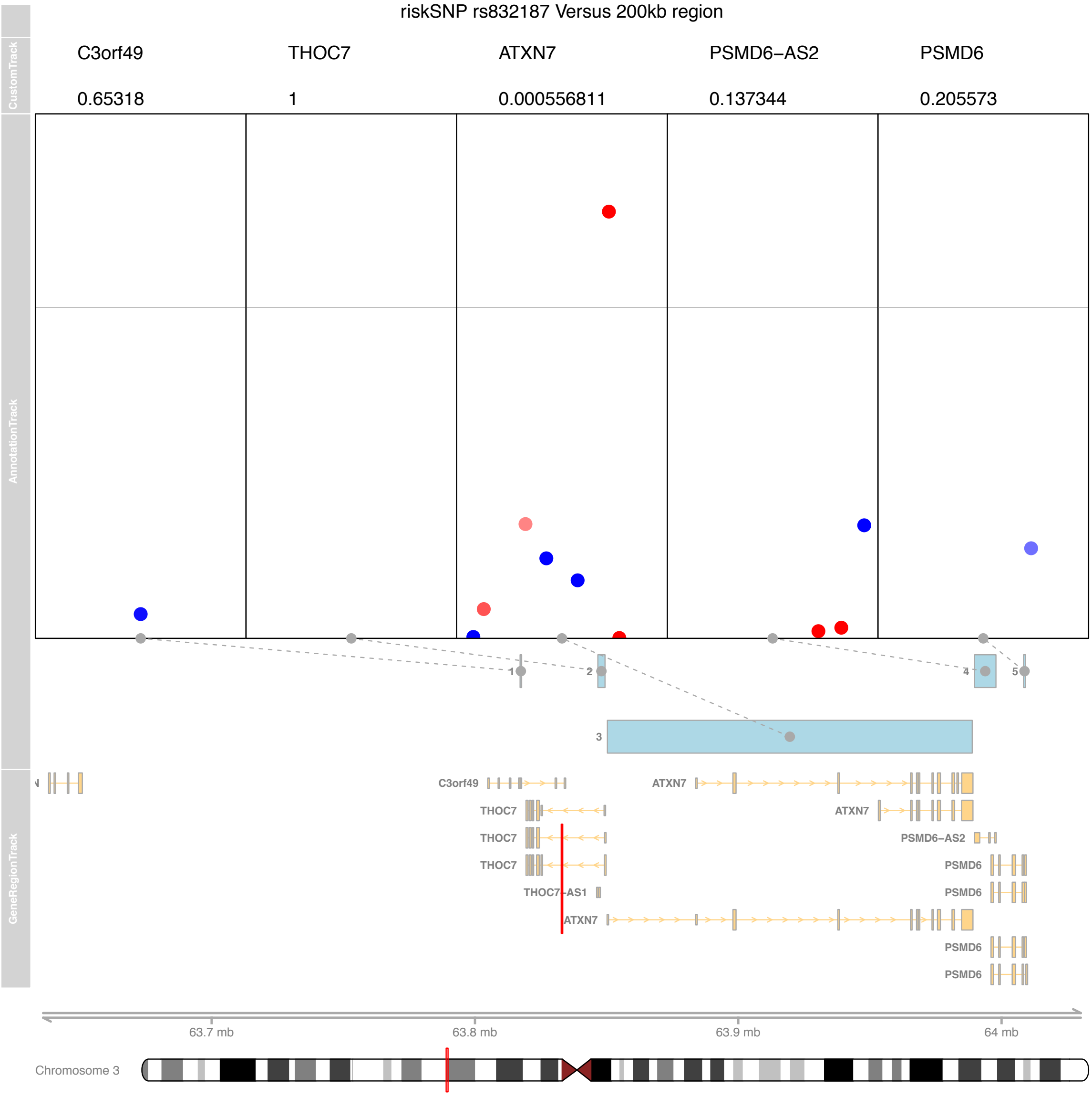

Figure S3-55. riskSNP-centric plot for Hippocampus similar to figure 3 in the main manuscript.

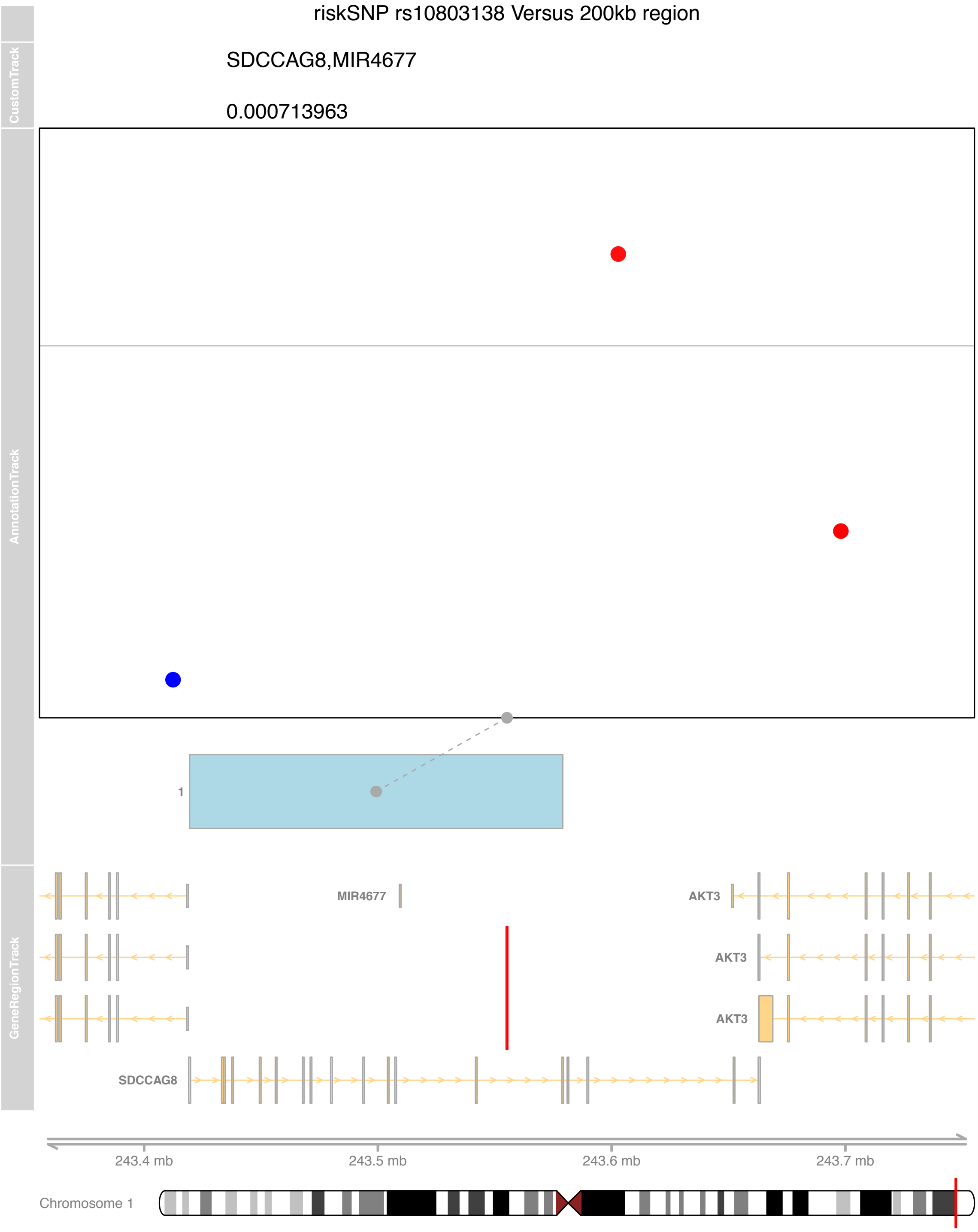

Figure S3-56. riskSNP-centric plot for Hippocampus similar to figure 3 in the main manuscript.

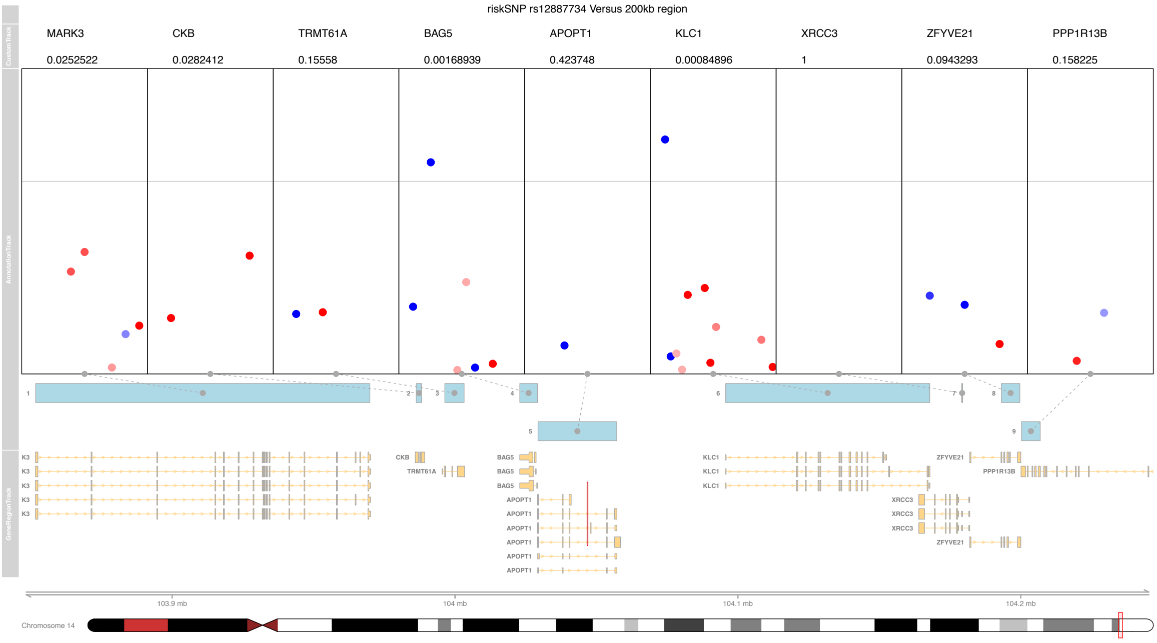

Figure S3-57. riskSNP-centric plot for Hippocampus similar to figure 3 in the main manuscript.

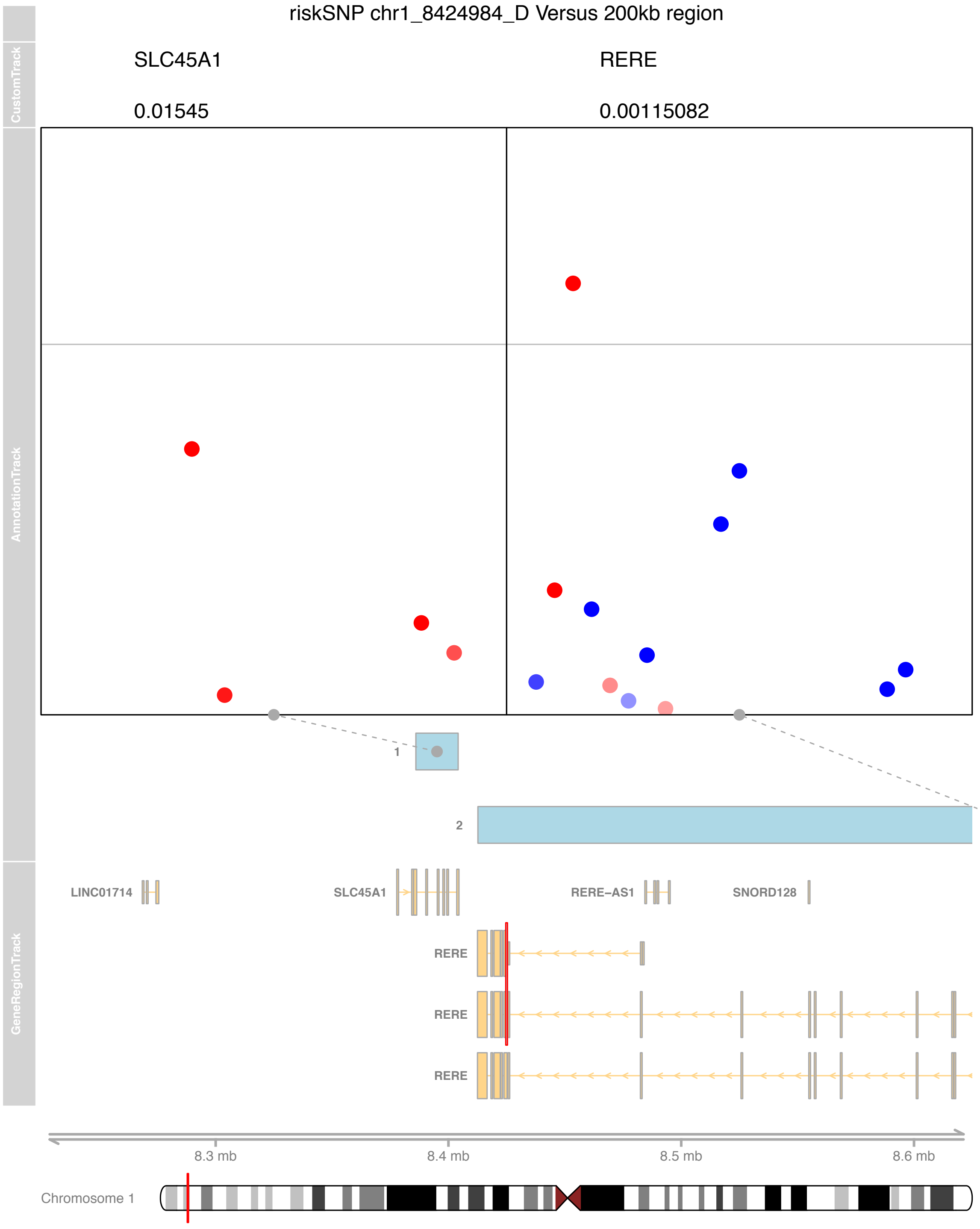

Figure S3-58. riskSNP-centric plot for Hippocampus similar to figure 3 in the main manuscript.

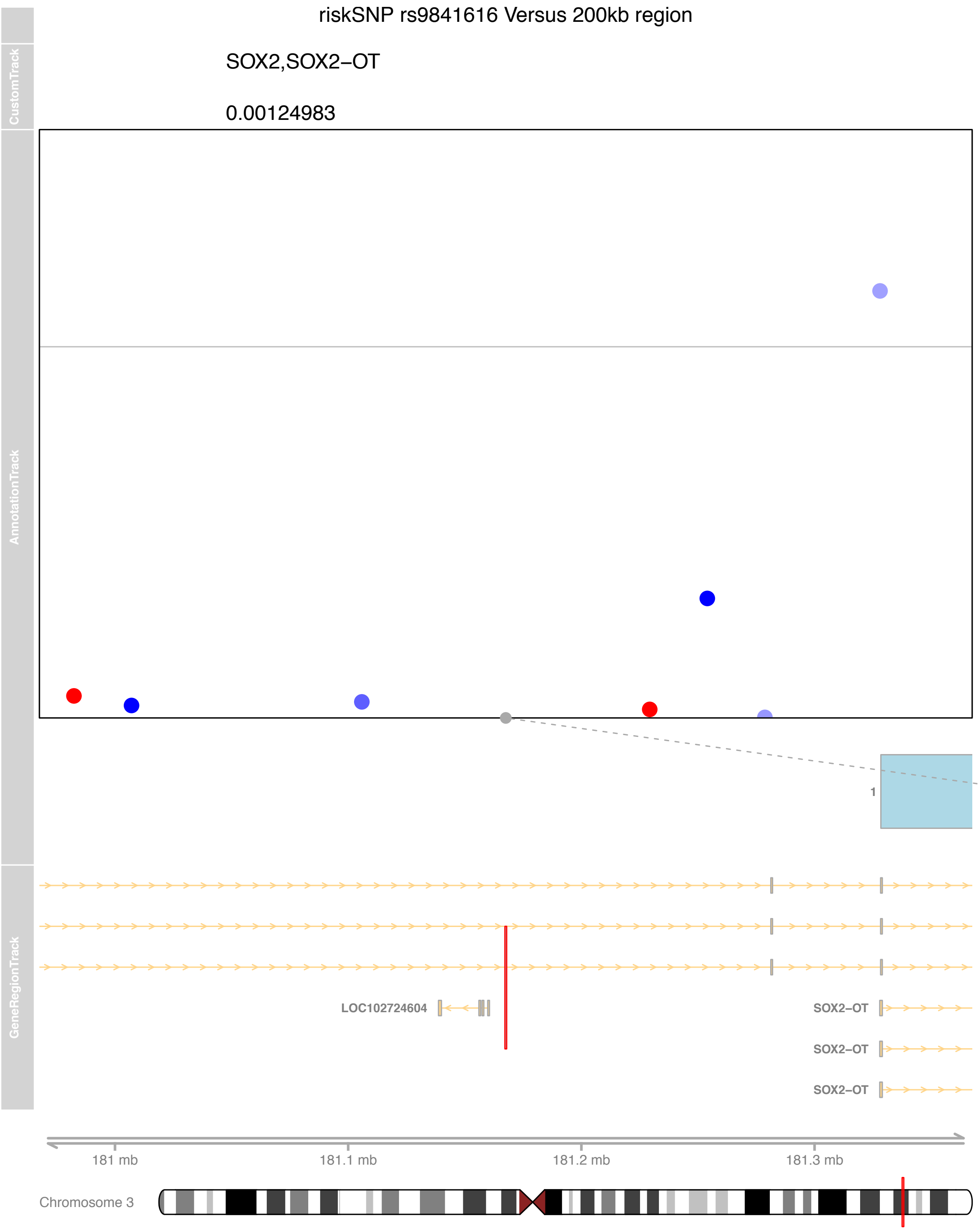

Figure S3-59. riskSNP-centric plot for Hippocampus similar to figure 3 in the main manuscript.

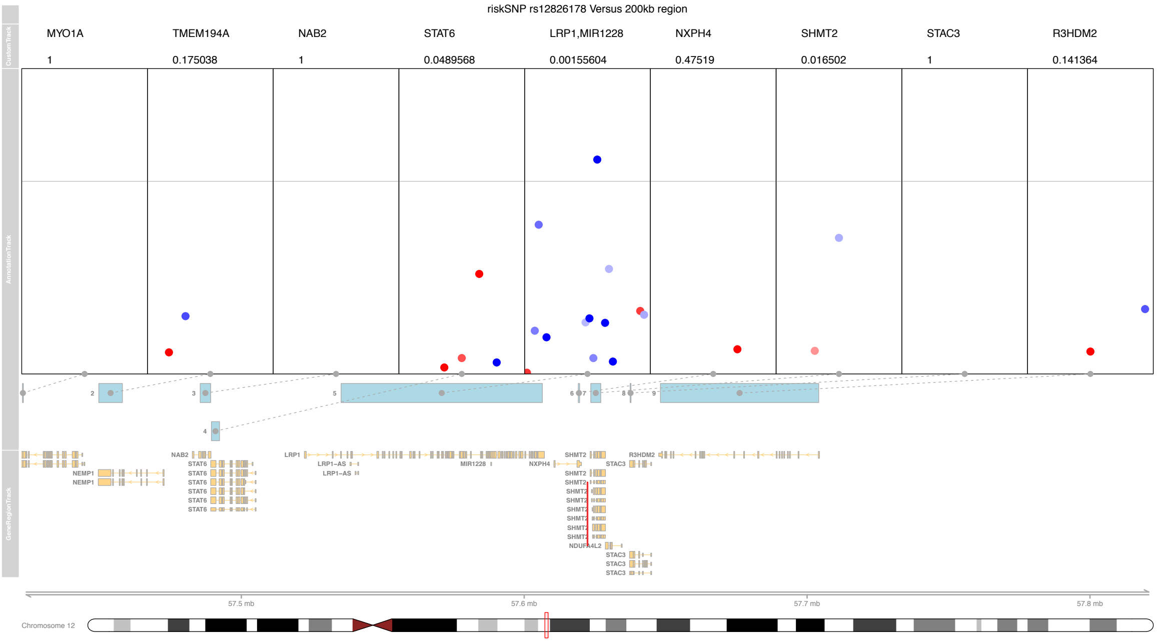

Figure S3-60. riskSNP-centric plot for Hippocampus similar to figure 3 in the main manuscript.

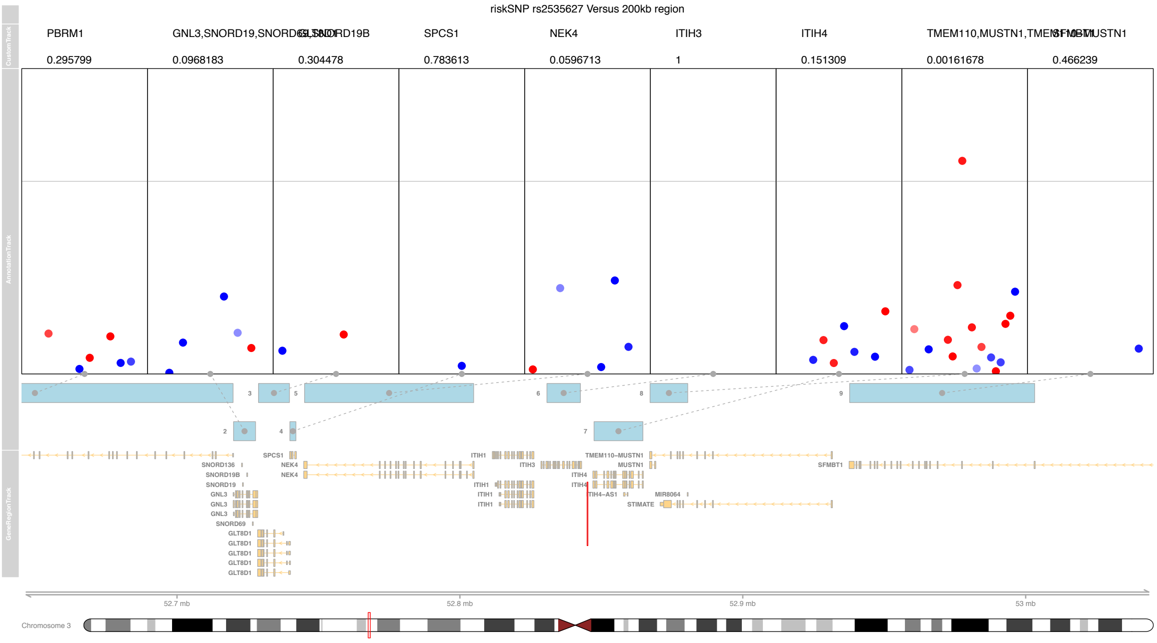

Figure S3-61. riskSNP-centric plot for Hippocampus similar to figure 3 in the main manuscript.

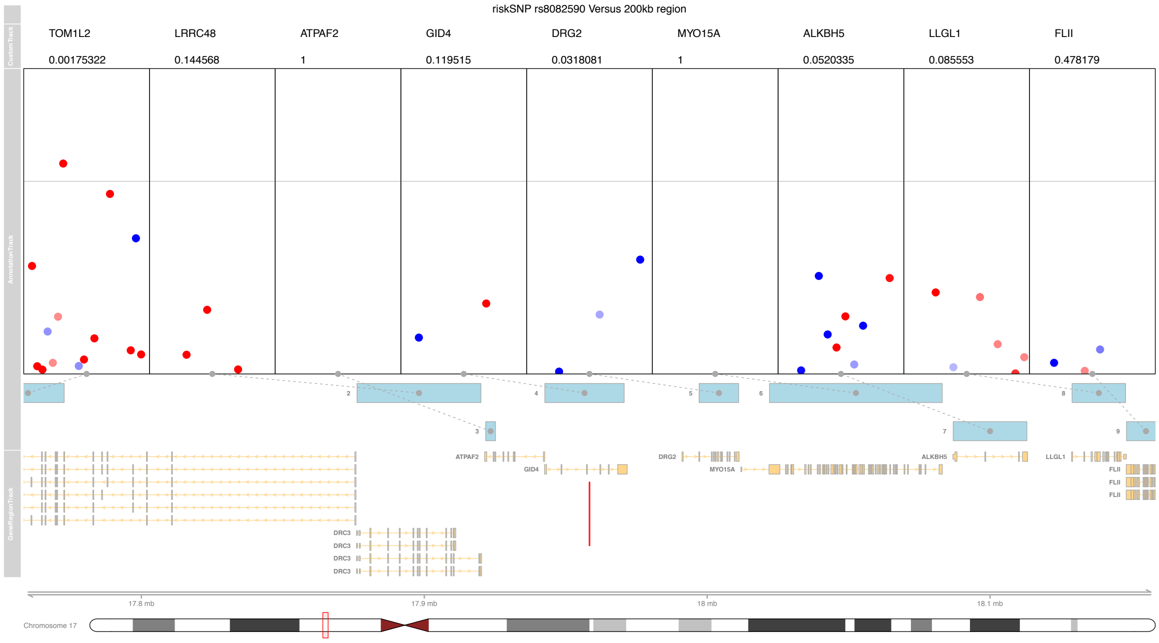

Figure S3-62. riskSNP-centric plot for Hippocampus similar to figure 3 in the main manuscript.

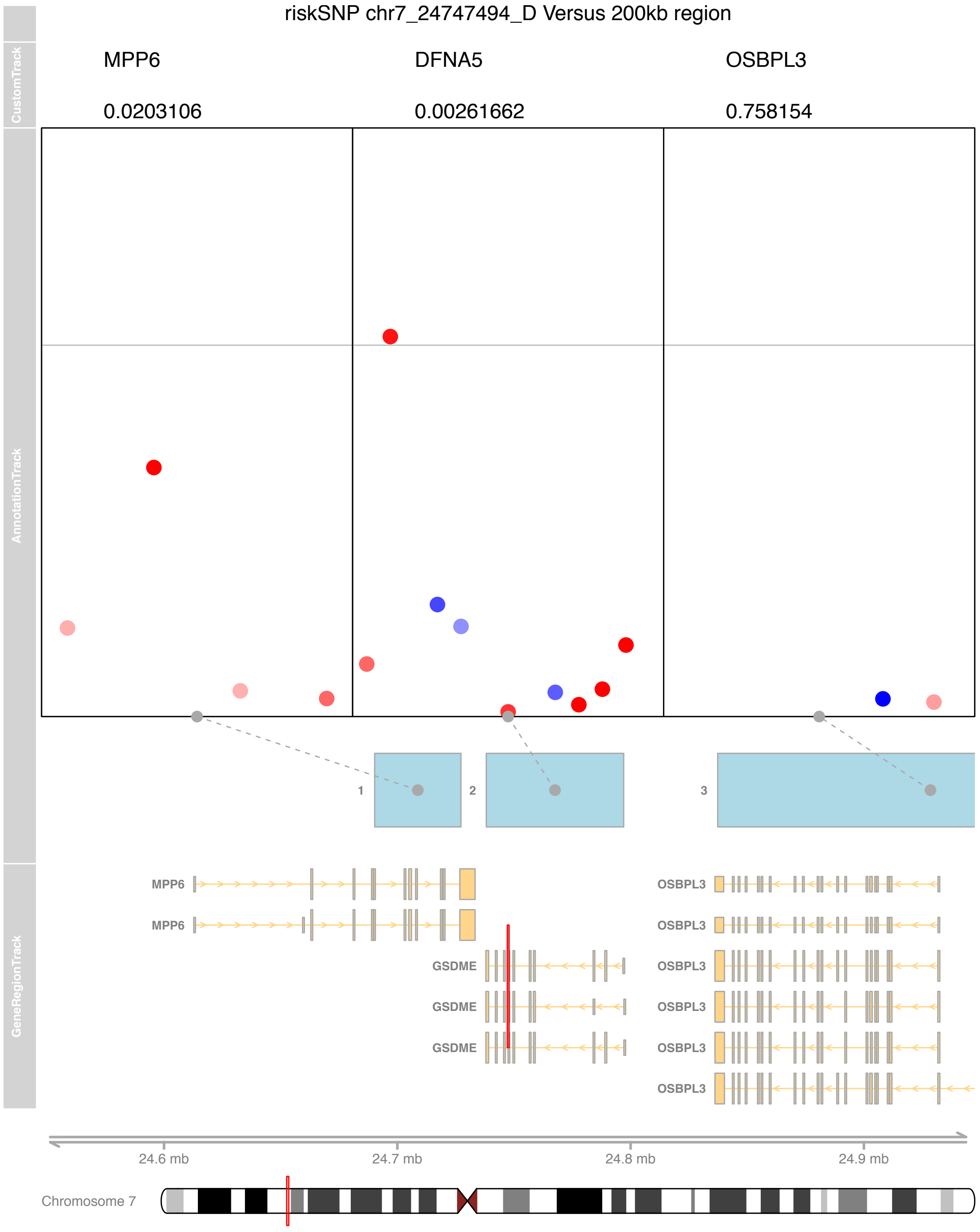

Figure S3-63. riskSNP-centric plot for Hippocampus similar to figure 3 in the main manuscript.

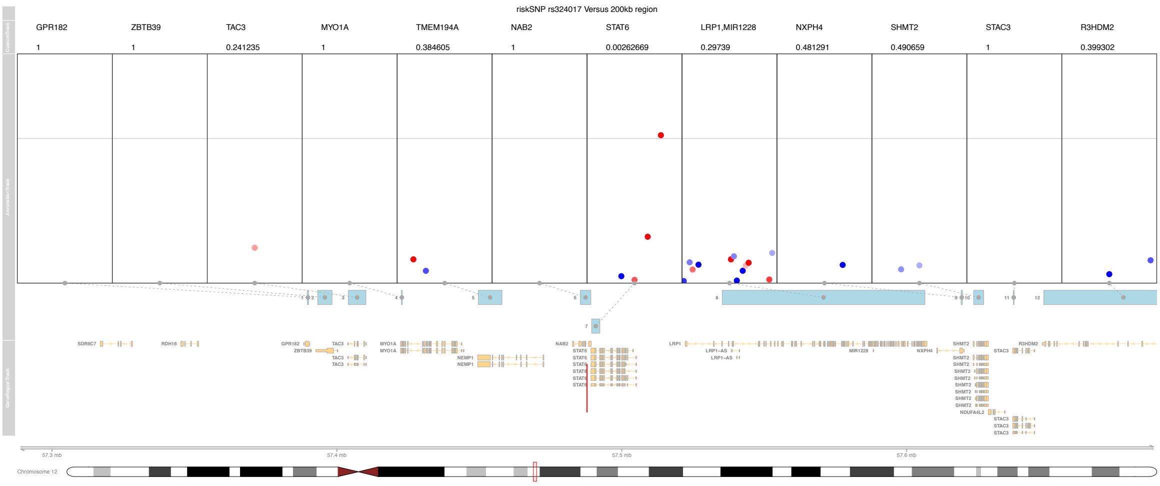

Figure S3-64. riskSNP-centric plot for Hippocampus similar to figure 3 in the main manuscript.
