# Supplementary material for: Comparison of quantitative trait loci methods: Total expression and allelic imbalance method in brain RNA-seq
Source: PLoS One. 2019 Jun 17;14(6):e0217765. doi: 10.1371/journal.pone.0217765 (PMC6576752; doi:10.1371/journal.pone.0217765)
Supplement: S4 File — A more detailed description of the pre-processing and analysis workflow. (PDF) [file pone.0217765.s004.pdf]

Allelic Imbalance in SZ (Supplemental Material 4)

This is a document containing supplemental fgures and tables for the “Comparison of Quantitative Trait Loci methods: Total Expression and Allelic Imbalance Method in Brain RNA-seq" manuscript.

General

- The GRCh37/hg19 assembly was used throughout the whole project
- The main workf ow was based on post-natal samples (Age > 0). But in the supplementary analysis we present a table similar to the main manuscript table1, including fetal samples.

Data input

- Three Genotype arrays (Illumina Human1M-Duo v3.0, illumina 650K and omniX+ microarrays).
- Two RNA sequencing FASTQ-format data sets from hippocampus and dorsolateral prefrontal cortex (DLPFC) tissues.
- The article by Ripke et al, describing 108 Risk Loci.
- Clinical phenotypes, including age and sex.

Pre-processing 1 - Initial summarizations and calculations

Genotypes

1) Genotypes were phased and imputed using shapeit(v2.r790) and impute2(version 2.3.2). As reference panel the 1000 Genomes phase III was used.
2) The phased and imputed genotype f les from each of the three microarray types were merged into one large VCF-f le including all samples (also fetal samples).
3) A PCA was performed on the VCF f le, and exported the resulting score as a matrix in R.

RNA-seq data

4) The RNA-seq data was mapped to two versions of the reference genome: hg19-default and an N-masked dbSNP version, using the STAR aligner on default settings (version 2.4.2a). This N-masked dbSNP had the location of all common SNPs (MAF>1%) replaced with N.

RNA-seq data (total expression)

5) From the mapped RNA-seq data we summarized read counts using TMM-normalization, this was exported as a matrix in R.
6) PCA on the summarized read counts. (one version for no-fetal samples and one for with-fetal samples)

RNA-seq data (allele specif c expression)

7) From the mapped RNA-seq data we summarized the allele specif c expression, notated txSNPs and exported the information for each gene to R, as multiple ASEsets(class of objects in the AllelicImbalance package).

Risk Loci information

8) An *AllelicImbalance* R-object containing the risk-SNPs in the 108 Risk Loci was prepared, including information such as location for the SNPs, clinical phenotypes, genotype PCs and expression PCs for the samples.

Preprocessing 2 - Filtering

SNP Filtering

9) The VCF phase information was added to the risk-SNP and tx-SNP data.
10) Risk-SNPs and tx-SNPs were filtered out if there was no phase information, had duplicates, or were multiallelic.
11) Risk-SNPs and tx-SNPs were filtered out if there were duplicates.

Sample filtering

12) Samples without a mapping key between RNA-seq and genotypes were excluded.
13) Filtered out fetal samples from the main analysis (kept in a supplemental material version of table1).

Annotation filtering

14) Kept only the tx-SNPs within the exons of the genes (using refseq annotation).

Read count filtering

15) Kept only txSNPs for which heterozygote samples existed.
16) Kept only txSNPs were each allele had at least 10 read counts per allele.
17) Kept only txSNPs were the fraction was more than 0.1 for the least expressed allele.

Main analysis

Regression

For both eQTL and aeQTL we were using the covariates age, sex, three first genotype PCs and the 10 first expression PCs. Because of major developmental gene expression differences at different age-groups, age was used also as a binary variable separating at 13 years for the with-fetal samples (supplemental materials).

18) Summarization of fraction values for each gene region.
19) For the regression analysis we selected only the gene-snp pairs which had at least 5 samples for at least two genotype groups.
20) Linear regression for each gene region fraction and every samples phased risk variant in the +/-200kb region.
21) Linear regression for each txSNP fraction and every samples phased risk variant in the +/-200kb region.
